# Living supramolecular polymerization of fluorinated cyclohexanes

**DOI:** 10.1038/s41467-021-23370-y

**Published:** 2021-05-25

**Authors:** Oleksandr Shyshov, Shyamkumar Vadakket Haridas, Luca Pesce, Haoyuan Qi, Andrea Gardin, Davide Bochicchio, Ute Kaiser, Giovanni M. Pavan, Max von Delius

**Affiliations:** 1 Institute of Organic Chemistry, University of Ulm, Ulm, Germany; 2 Department of Innovative Technologies, University of Applied Sciences and Arts of Southern Switzerland, Lugano-Viganello, Switzerland; 3 Central Facility of Electron Microscopy, Electron Microscopy Group of Materials Science, University of Ulm, Ulm, Germany; 4 Center for Advancing Electronics Dresden (cfaed) and Faculty of Chemistry and Food Chemistry, Technical University of Dresden, Dresden, Germany; 5 Department of Applied Science and Technology, Politecnico di Torino, Torino, Italy; 6 Department of Physics, Università degli studi di Genova, Genova, Italy

**Keywords:** Self-assembly, Supramolecular polymers

## Abstract

The development of powerful methods for living covalent polymerization has been a key driver of progress in organic materials science. While there have been remarkable reports on living supramolecular polymerization recently, the scope of monomers is still narrow and a simple solution to the problem is elusive. Here we report a minimalistic molecular platform for living supramolecular polymerization that is based on the unique structure of all-cis 1,2,3,4,5,6-hexafluorocyclohexane, the most polar aliphatic compound reported to date. We use this large dipole moment (6.2 Debye) not only to thermodynamically drive the self-assembly of supramolecular polymers, but also to generate kinetically trapped monomeric states. Upon addition of well-defined seeds, we observed that the dormant monomers engage in a kinetically controlled supramolecular polymerization. The obtained nanofibers have an unusual double helical structure and their length can be controlled by the ratio between seeds and monomers. The successful preparation of supramolecular block copolymers demonstrates the versatility of the approach.

## Abstract (web summary)

Living supramolecular polymerization can produce precise covalent polymers, but the scope of monomers is still narrow. Here the authors show a molecular platform for living supramolecular polymerization that is based on the unique structure of all-cis 1,2,3,4,5,6- 22 hexafluorocyclohexane, the most polar aliphatic compound reported to date.

## Introduction

Controlled polymerization methods such as atom transfer radical polymerization^1^ have revolutionized polymer chemistry by endowing artificial macromolecules with a degree of structural precision that is only surpassed by biopolymers^2^. By providing facile access to homo- and block copolymers of various composition and topology, living polymerization (LP)^3^ has paved the way toward diverse applications in solar cell manufacturing^4^, nanophotonics^5^ and biomedicine^6^. A similar transformation toward precision materials is currently underway in supramolecular polymer chemistry, a field that is only three decades old^7–9^ and increasingly finds applications^10–12^. In 2014, Takeuchi and Sugiyasu reported the first example of living supramolecular polymerization (LSP) based on the aggregation of a porphyrin derivative into metastable nanoparticles that could be converted into thermodynamically more stable nanofibers of relatively precise length by the addition of nanofiber seeds and subsequent kinetically controlled chain-growth^13^. Many recent examples of LSP are based on a similar use of off-pathway aggregates^14^, which can be self-assembled from diverse building blocks such as rylene dyes^15–21^, (aza)-BODIPY dyes^22,23^, N-heteroangulenes^24^ and amphiphilic Pt^II^ complexes^25^. Further examples make use of the counter-anion modulated aggregation of Pt^II^ and Pd^II^ pincer-type complexes^26^, the coupling of SP with a chemical fuel or light^27–30^, the trapping of an active monomer using “dummy” monomers incapable of 1D supramolecular polymerization^31^ and the amplification of macrocycles from dynamic combinatorial libraries^32^. Moreover, supramolecular precision materials with remarkable complexity have been obtained via living epitaxial growth^33–44^.

However, for LSP to become a general and versatile synthetic method, a simple and minimalistic molecular platform should be developed that is easily functionalized and allows a predictable fine-tuning of LSP characteristics. Arguably, the most important step in this direction would be to avoid off-pathway aggregates and use instead the predictable folding of a monomeric core fragment into a metastable state^45–49^. A breakthrough to this end was recently reported by Aida et al. who have designed a sophisticated *C*_5_-symmetric corannulene derivative that is prevented from spontaneous polymerization by the formation of a network of intramolecular hydrogen bonds within the amide groups of the five side chains^50^. By addition of a molecularly dissolved, *N*-methylated corannulene derivative, the authors were able to initiate LSP and demonstrate control over the length of the formed fibers^51^. When analyzing the design aspects behind this groundbreaking study, one cannot help but notice that the crucial role of the concave corannulene motif, namely to impart directionality to the growing fibers, could perhaps be realized based on a simpler molecular platform. Specifically, it occurred to us that all-cis 1,2,3,4,5,6-hexafluorocyclohexane (C_6_H_6_F_6_) with its enormous dipole moment^52^, straightforward synthesis^53^ and emerging potential as a supramolecular host^54^ would be an excellent candidate. Furthermore, theoretical studies predict a large and cooperative enhancement of the dipole moment during aggregation of all-cis C_6_H_6_F_6_ into one-dimensional stacks^55^. We wondered whether this cooperativity could be used to drive the formation of thermodynamically stable supramolecular polymers, while the large dipole moment of all-cis C_6_H_6_F_6_ (6.2 Debye) could lead to folded, metastable states that would allow us to perform LSP.

**Fig. 1:**
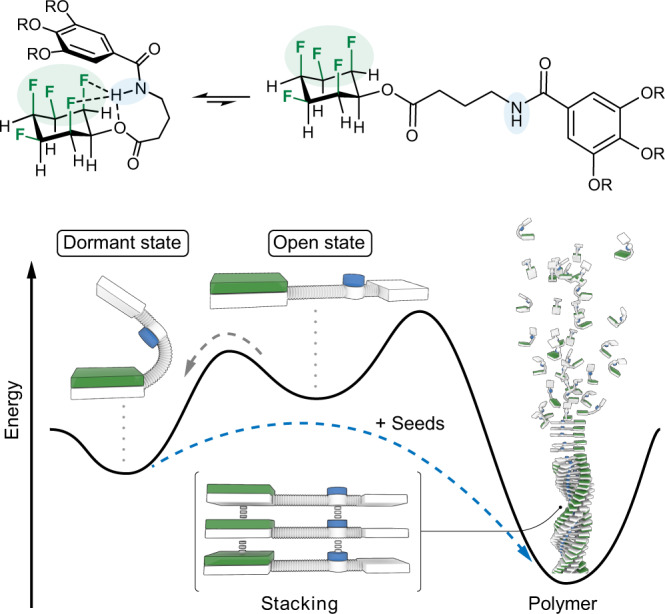
General structure of monomers and energy landscape of supramolecular polymerization. Top: general molecular design of the key monomer in a representative folded and the unfolded state. Bottom: schematic illustration of the partial energy profile of LSP. Folding of a monomer into dormant states inhibits spontaneous aggregation, whereas seeds can initiate LSP.

In this work we demonstrate that simple derivatives of all-cis 1,2,3,4,5,6-hexafluorocyclohexane (Fig. 1) indeed give rise to LSP and that supramolecular block copolymers can be prepared using this approach. We show that the key monomer is kinetically trapped due to folding and that well-defined seeds initiate the growth of fibers, whose average length can be controlled by the ratio of seeds to monomer (Fig. 1).

## Results and discussion

### Molecular design and synthesis

**Fig. 2:**
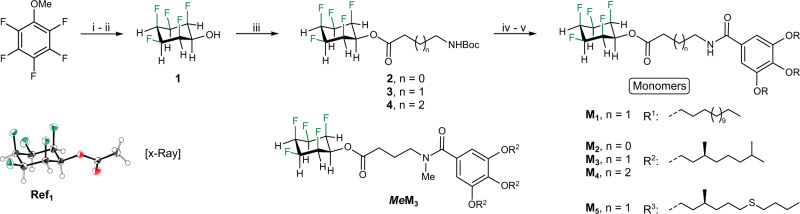
Synthesis of compounds used in this study. Conditions: (i) Rh(CAAC)(COD)Cl, SiO_2_, H_2_ (80 atm.), n-hexane, r.t., 24 h;^53^ (ii) n-BuSH, AlCl_3_, CH_2_Cl_2_, r.t., 12 h, 83%; (iii) **S1**–**S3**, DMF, Et_3_N, 120 °C, 2 h, 92–100%; (iv) TFA, CH_2_Cl_2_, r.t., 2 h; (v) **2**–**4**, DMF, Et_3_N, room temperature, 12 h, 23–87%. Color code: green: F; red: O; gray: C.

Our monomer design is based on all-cis 2,3,4,5,6-pentafluorocyclohexan-1-ol, which is linked via an ester bond, a short alkyl chain and an amide bond to an alkylated derivative of gallic acid that imparts solubility (Fig. 2). Monomer **M**_**1**_ is achiral, whereas monomers **M**_**2**_–**M**_**5**_ bear stereogenic centers (for their synthesis and characterization, see Supplementary Methods), allowing the use of circular dichroism (CD) spectroscopy to study the kinetics of self-assembly.

The synthesis of monomers starts from commercially available pentafluoroanisol, which was hydrogenated according to a procedure reported by Glorius^53^ and deprotected to furnish all-cis 2,3,4,5,6-pentafluorocyclohexan-1-ol (Fig. 2). Esterification furnished compounds **2**–**4** quantitatively by reaction with pentafluorophenol-based active esters. The single-crystal X-ray structure of all-cis 2,3,4,5,6-pentafluorocyclohexyl acetate (**Ref**_**1**_) confirms the equatorial position of the ester group and the all-cis arrangement of the fluorine atoms in compounds of this general structure. Straightforward deprotection and coupling steps gave the desired monomers in good to excellent yields. All reactions were found to be scalable and reproducible, allowing gram-scale syntheses of all monomers reported herein.

During column chromatography of **M**_**1**_, we observed the formation of thick crystalline fibers on the liquid/air interface (Supplementary Figs. 5–7) inside a test tube. Propagation from the interface into the bulk solution led to complete gelation within 10–15 min (Supplementary Figs. 5, 8), while fiber formation appeared to be initially absent in the bulk solution. This unusual gelation behavior of achiral monomer **M**_**1**_ was a promising lead result that convinced us to prepare its more soluble, chiral analogue **M**_**3**_ to study its self-assembly in greater detail.

### Monomer folding inhibits spontaneous supramolecular polymerization

**Fig. 3:**
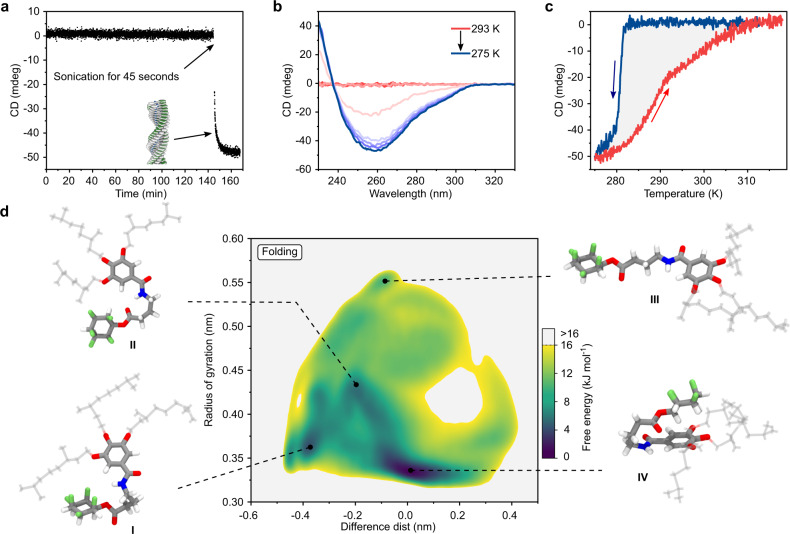
Studies on monomer folding. **a** CD monitoring (260 nm) of **M**_**3**_ (1.2 mM) in cyclohexane/chloroform (84:16 v/v) at 293 K, indicating remarkable kinetic stability. **b** Temperature-dependent CD spectra of **M**_**3**_ (1.2 mM) in cyclohexane/chloroform (84:16 v/v) upon cooling from 293 K to 275 K (0.5 K min^−1^). **c** CD monitoring (260 nm) of cooling (blue) and heating (red) of a 1.2 mM solution of **M**_**3**_ in cyclohexane/chloroform (84:16 v/v) at a rate of 0.5 K min^−1^. Once nucleation starts in a concentrated solution at temperatures for which ΔG is already negative, polymerization proceeds very rapidly since the concentration of free monomer in solution is high, leading to a very sharp transition during cooling (blue line). **d** Free energy surface (FES) estimated from WT-MetaD simulations, showing monomer conformations and their relative free energies as a function of collective variables CV1 (*x*-axis: difference between the distance between pentafluorocyclohexyl-Hs and the amide oxygen and the distance between the pentafluorocyclohexyl-Fs and the amide nitrogen) and CV2 (*y*-axis: radius of gyration of **M**_**3**_ monomer core). Blue colored areas represent the low free energy domains while the gray colored background is high in free energy or not accessible. Four representative monomer conformations (I, II, III, IV) taken from the FES are reported (alkyl chains are shown in transparency for clarity). Color code: green: F; red: O; blue: N; gray: C.

Our initial studies on supramolecular polymerization were carried out in a mixture of cyclohexane and chloroform (84:16 v/v, standard solvent) because time-dependent CD measurements of a 1.2 mM solution of **M**_**3**_ at 293K, revealed that in this medium, **M**_**3**_ is remarkably kinetically persistent and remains molecularly dissolved for hours. However, ultrasonication for 45 s induces polymerization (Fig. 3a), forming a clear viscous solution. Slow cooling (0.5 K min^−1^) of a 1.2 mM solution of **M**_**3**_ resulted in a pronounced negative Cotton effect with an absorption maximum at 260 nm, indicating the formation of chiral supramolecular aggregates (Fig. 3b). The transition from the dissolved to the polymerized state occurs abruptly with an elongation temperate (T_e_) of 282K. The AFM height image of a cooled (275K) solution of **M**_**3**_ spin-coated on a silicon wafer reveals a dense fibrous network (Supplementary Fig. 7). No evidence for supramolecular aggregates was observed when an analogous study was carried out with reference compounds **Ref**_**2**_ (conventional cyclohexane) and ***Me*M**_**3**_ (methylated amide), indicating that both the all-cis C_6_H_6_F_5_ motif and the amide bond are crucial for supramolecular polymerization (Supplementary Fig. 9).

Variable temperature CD measurements of **M**_**3**_ at slow cooling and heating rates (0.5 K min^−1^) revealed pronounced hysteresis (Fig. 3c). For monomer **M**_**2**_, in which only two methylene groups separate all-cis C_6_H_6_F_5_ and the amide bond, hysteresis was also observed but the T_e_ during cooling was 11 K higher (Supplementary Fig. 10), while for monomer **M**_**4**_ hysteresis was negligible and oligomeric species were observed already at 328 K (Supplementary Fig. 10). The observation of hysteresis and the distinct differences observed for **M**_**2**_, **M**_**3**,_ and **M**_**4**_ can be explained by the varying ability of these monomers to form intramolecular interactions such as hydrogen bonds^56^, whose strength should heavily depend on the length of the spacer.

We therefore proceeded to gather experimental and computational evidence for monomer folding. First, we investigated whether the kinetic stability of **M**_**3**_ (Fig. 3a) may be a result of intramolecular interactions by studying the kinetics of the polymerization at different concentrations at a constant temperature of 293 K. At all concentrations studied, we observed sigmoidal kinetic curves with lag times decreasing upon increasing concentration of **M**_**3**_ (Supplementary Fig. 11). DLS analyses performed on 1.2 mM solution of **M**_**3**_ in the solvent mixture used for kinetic studies (cyclohexane/chloroform 84:16 v/v) displayed a unimodal scattering peak with a hydrodynamic diameter of ~ 1.1 nm (Supplementary Fig. 12). The diffusion-ordered spectroscopy (DOSY) of **M**_**3**_ (1.2 mM in C_6_D_12_/CDCl_3_ 84:16 v/v at 295 K) provided a diffusion coefficient similar to non-assembling and non-folding reference compound **Ref**_**2**_ (Supplementary Figs. 13, 14). While these results clearly indicate that no off-pathway aggregates are involved, we also attempted to study folding more directly by variable temperature ^1^H NMR spectroscopy. To this end, a 1.2 mM solution of **M**_**3**_ in C_6_D_12_/CDCl_3_ (84:16 v/v) was cooled from 333 to 283 K, revealing a strong temperature-dependence of the chemical shift of the amide and aromatic protons, which is in agreement with the expected temperature-dependence of the equilibrium between unfolded and folded states (Supplementary Figs. 15, 16). In a ^19^F/^1^H-HOESY NMR experiment on **M**_**1**_ in 30 mM solution (CDCl_3_), we observed weak ^19^F/^1^H NOE of two fluorine atoms in all-cis C_6_H_6_F_5_ with both amide and aromatic protons (Supplementary Fig. 18). Fourier-transform infrared spectral measurement (FT-IR) performed with 1.2 mM solution of **M**_**3**_ C_6_H_12_/CHCl_3_ (84:16 v/v) revealed no presence of stretching frequency corresponding to hydrogen-bonded amide hydrogens but small shifts of amide I band which could be attributed to the formation of CH···π interactions between all-cis C_6_H_6_F_5_ and aromatic ring (Supplementary Figs. 23, 24). Combined HOESY and FTIR results confirm that folding is indeed present but likely not limited to a single structure and conventional C=O···H–N intramolecular hydrogen bond.

To gain a deeper understanding of monomer folding, we carried out all-atom well-tempered metadynamics (WT-MetaD)^57^ simulations of **M**_**3**_ in explicit solvent molecules (C_6_H_12_/CHCl_3_ in the ratio of 84:16 v/v; see Supplementary Methods for more details). These simulations allowed us to compute the free energy surface (FES) of the monomer folding/unfolding (Fig. 3d). The conformations accessible to the monomer are identified in the FES as a function of the two collective variables (CV1 and CV2) used during the WT-MetaD: CV1 represents the orientation of pentafluorocyclohexyl with respect to the amide-carbonyl group (Supplementary Fig. 25c), and CV2 is indicative of the opening of the monomer “core” (Supplementary Fig. 25b). The FES shows that the monomer **M**_**3**_ tends to assume a folded conformation (Fig. 3d: dark blue region characterized by low CV2 values), in which the hydrogen atoms of pentafluorocyclohexyl are close to the benzene moiety (Fig. 3d: IV conformer)^58^. Other (less) energetically accessible conformations (within ~5 kJ/mol from the global free energy minimum) are favored either by the interaction of the fluorine atoms with the amide-H (Fig. 3d: I conformer) or by the formation of a hydrogen bond with the carbonyl group (II conformer). Similar analyses were performed on **M**_**2**_, **M**_**4**_ and ***Me*M**_**3**_ (Supplementary Fig. 26), showing that for **M**_**2**_ and ***Me*M**_**3**_ the opening of the monomer is much more likely than for **M**_**3**_ and **M**_**4**_.

### Living supramolecular polymerization

**Fig. 4:**
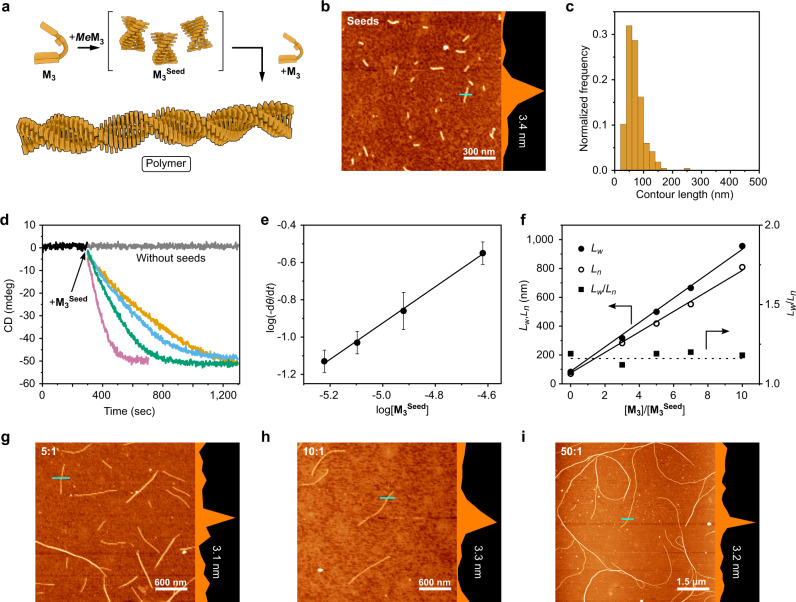
Living supramolecular polymerization. **a** Schematic illustration of seeded living supramolecular polymerization. **b** AFM height image of **M**_**3**_^**Seed**^ spin-coated on a silicon wafer. Cross-section was measured along the blue line. **c** Histogram of the length distribution of **M**_**3**_^**Seed**^. **d** Time course of supramolecular polymerization of **M**_**3**_ initiated by addition of different amounts of **M**_**3**_^**Seed**^ ([**M**_**3**_]/[**M**_**3**_^**Seed**^] = 50:1 (pink curve), 100:1 (green curve), 150:1 (blue curve), 200:1 (yellow curve) v/v). **e** Log-log plot of the rate of polymerization as a function of **M**_**3**_^**Seed**^ concentration. Error bars correspond to one standard deviation of a triplicate measurement. Solid line – linear fit with slope 0.97 (correlation coefficient 0.998). **f** Weight-average length (*L*_*w*_), number-average length (*L*_*n*_) and PDI (*L*_*w*_/*L*_*n*_) obtained by measuring AFM length of supramolecular polymers obtained with different [**M**_**3**_]/[**M**_**3**_^**Seed**^] ratios (3:1, 5:1, 7:1, 10:1 v/v) plotted against [**M**_**3**_]/[**M**_**3**_^**Seed**^] v/v. Solid lines represent linear fit (correlation coefficients 0.995) indicating linear dependence of the size of supramolecular polymers on the amount of **M**_**3**_^**Seed**^ added. Dashed line serves as guide for the eye. **g**–**i** Representative AFM height images of supramolecular polymers obtained with different [**M**_**3**_]/[**M**_**3**_^**Seed**^] ratios ((**g**) 5:1, (**h**) 10:1, (**i**) 50:1 v/v) spin-coated on silicon wafer.

Having gained an understanding of the folding of monomer **M**_**3**_ into kinetically trapped states, we proceeded to test whether we could use this molecular property to facilitate LSP. Initially, we attempted to use the *N*-methylated analogue ***Me*M**_**3**_ as a molecular initiator based on the assumption that it is less prone to folding, but could interact with **M**_**3**_ in a way that triggers unidirectional polymerization^51^. While this initiation mode was not successful, we were able to develop a protocol that makes use of well-defined seeds to trigger LSP. **M**_**3**_^**Seed**^ was prepared by sonication of a mixture of **M**_**3**_ and ***Me*M**_**3**_ (c_tot_ = 1.2 mM, **M**_**3**_/***Me*M**_**3**_ = 3:1 mol/mol) in standard solvent at 273 K for 20 min (Fig. 4b). In the absence of ***Me*M**_**3**_, we obtained longer, non-uniform fibers (Supplementary Fig. 27) that have a higher tendency to bundle and which induce polymerization with decreased reproducibility and higher polydispersity (Supplementary Fig. 30). Based on the effect that the **M**_**3**_/***Me*M**_**3**_ ratio has on the size of seeds (Supplementary Figs. 27, 28) we propose that ***Me*M**_**3**_ acts as a sequestrator^59^ that competitively interacts with **M**_**3**_ and therefore reduces the available amount of **M**_**3**_ that can be incorporated into polymer chains. The sequestrator ***Me*M**_**3**_ thus lowers the degree of polymerization, which results in a more effective size control during seed formation (Supplementary Discussion). This optimized procedure allowed us to prepare **M**_**3**_^**Seed**^ fibers with a weight-averaged length (*L*_*w*_) of 83 nm and a polydispersity index (PDI) of ca. 1.2 (Fig. 4b, c). When we added different volumes of the **M**_**3**_^**Seed**^ solution ([**M**_**3**_]/[**M**_**3**_^**Seed**^]: 50:1, 100:1, 150:1 and 200:1 volume ratios) to a solution of **M**_**3**_ (1.2 mM, 293 K), polymerization started without lag time and was complete within 2–15 min (Fig. 4d). The crucial plot shown in Fig. 4e confirms that the logarithm of the apparent polymerization rate (log(*-dθ*/*dt*)) is directly proportional to the logarithmic amount of **M**_**3**_^**Seed**^ initiator, which is expected for a well-behaved chain-growth polymerization.

The size distributions of supramolecular polymers prepared by seeded LSP were determined by atomic force microscopy (AFM) under carefully optimized conditions (Fig. 4f). The obtained AFM images revealed individual, uniform, separate fibers, alongside bundles, which could however clearly be distinguished based on image contrast and AFM height profile (Fig. 3g). Weight-averaged lengths (*L*_w_) of 955, 665, 499, and 317 nm, could be determined for individual fibers prepared from [**M**_**3**_]/[**M**_**3**_^**Seed**^] ratios of 3:1, 5:1, 7:1 and 10:1 (v/v), respectively (Fig. 4g, h and Supplementary Fig. 33). The linearity of the plot of fiber length versus [**M**_**3**_]/[**M**_**3**_^**Seed**^] (Fig. 4f) provides further evidence for the living nature of the observed polymerization. When using a [**M**_**3**_]/[**M**_**3**_^**Seed**^] ratio of 20:1 we observed polymers with a length of ca. 1–3 µm, whose bundling precluded reliable statistical AFM analyses (Supplementary Fig. 34). Further increase of **M**_**3**_/**M**_**3**_^**Seed**^ to 50:1 led to the formation of even longer fibers (Fig. 4i). In all cases that allowed reliable statistical analyses, the PDI was below 1.2 (Supplementary Fig. 35 and Supplementary Table 1). Polymer chains remain active and continue to elongate upon addition of **M**_**3**_, as was demonstrated in multicycle dilution CD experiments.

### Supramolecular block copolymers

We wondered whether it would be possible to carry out kinetically controlled LSP of monomers with different side chains. This approach would be reminiscent of the living chain-growth polymerization of acrylates, where a molecule of interest is attached to an acrylate or acrylamide that undergoes living covalent polymerization. Instead of acrylates we would rely on all-cis 2,3,4,5,6-pentafluorocyclohexyl 4-amidobutanoates, which represent the core structure facilitating LSP, as was demonstrated above.

While there has been remarkable progress recently with preparing supramolecular block copolymers under thermodynamic control^60–62^ or by slow cooling of monomer mixtures^63–65^, a growing number of such precision polymers has been prepared by LSP, which can provide superior structural and sequence control. However to date, these LSP approaches either rely on the modification of the polymerizable core, e.g., substituents are attached to the core of rylene dyes^16,66^ or the complexation of different metal ions to identical ligands^67,68^. A modular approach based on the variation of the side chain, as pioneered by Otto, Faul, Manners and Sugiyasu^19,32,69^, would be complementary to these methods and could offer distinct advantages for applications in organic materials science. For obtaining a proof of principle, we decided to introduce a structural change to the aliphatic side chains in **M**_**3**_. Keeping the crucial design elements for LSP as well as the aromatic core and the stereogenic centres in place should allow us to use the same analytical techniques and correlate the observed data with **M**_**3**_^**Polymer**^. Based on this reasoning, we prepared monomer **M**_**5**_, which contains three thiobutyl group instead of isopropyl groups (Fig. 2). When performing LSP with this monomer, we were delighted to find that this considerable structural change (insertion of a heteroatom, side chain elongation and removal of a branching point) did not significantly affect the polymerization behavior. In general, **M**_**5**_ behaved similarly to **M**_**3**_, while it is kinetically more stable, such that a solution of **M**_**5**_ (1.2 mM, C_6_H_12_/CHCl_3_ 84:16 v/v) starts to polymerize only after prolonged ultrasonication (>7 min at RT). Therefore, we decided to decrease the polarity of our solvent mixture for the following block copolymer syntheses by increasing the volume fraction of cyclohexane to 93% (Supplementary Fig. 37).

**Fig. 5:**
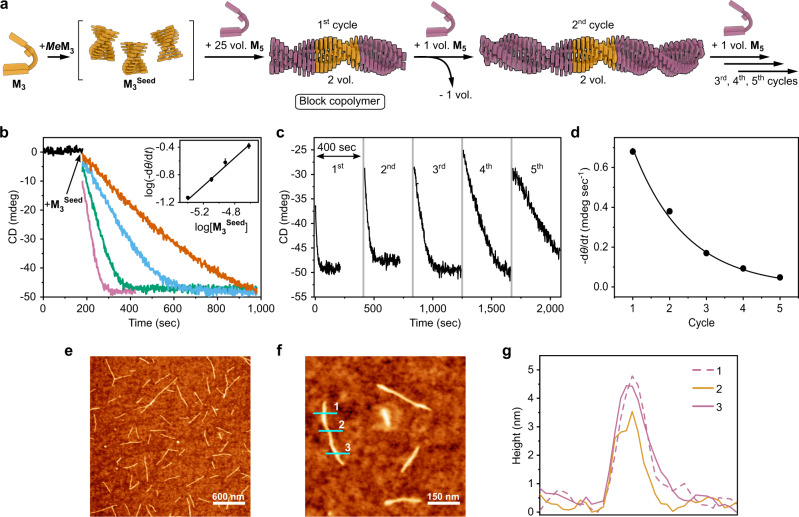
Supramolecular block copolymers. **a** Schematic illustration of the synthesis of block copolymers by seeded LSP in a multicycle kinetic experiment. **b** Time course of supramolecular polymerization of **M**_**5**_ initiated by addition of different amounts of **M**_**3**_^**Seed**^: [**M**_**5**_]/[**M**_**3**_^**Seed**^] = 50:1 (pink curve), 100:1 (green curve), 150/1 (blue curve), 300:1 (orange curve) v/v. Inset shows log-log plot of the rate of polymerization as a function of **M**_**3**_^**Seed**^ concentration. Error bars correspond to one standard deviation of a triplicate measurement. Solid line corresponds to linear fit with slope 1.02 (correlation coefficient 0.987). **c** Time course of change of the CD signal at 260 nm, while **M**_**5**_**-M**_**3**_**-M**_**5**_ supramolecular block copolymer obtained using [**M**_**5**_]/[**M**_**3**_^**Seed**^] = 25:1 ratio (1st cycle), was diluted with **M**_**5**_ over five cycles. Polymerization continues upon each addition of **M**_**5**_ but at increasingly slower rate. **d** Plot of initial slopes (sec^−1^) as a function of the cycle number. Solid line corresponds to exponential fit, demonstrating bisection of the slope during each cycle. **e** Representative AFM height image of **M**_**5**_**-M**_**3**_**-M**_**5**_ supramolecular block copolymers obtained using [**M**_**5**_]/[**M**_**3**_^**Seed**^] = 3:1 ratio, spin-coated on silicon wafer. **f**, **g** AFM height image and cross-section analyses along the blue lines demonstrating the presence of thicker termini, corresponding to **M**_**5**_ block, and thinner middle part, corresponding to **M**_**3**_ block.

To prepare an ABA type block copolymer we used **M**_**3**_^**Seed**^ polymer (golden, Fig. 5a) to initiate the supramolecular polymerization of the sulfur-containing monomer **M**_**5**_ (pink, Fig. 5a). CD spectroscopy revealed polymerization kinetics similar to those observed for LSP of **M**_**3**_ (Fig. 5b). Polymerization of **M**_**5**_ starts without lag time with rates proportional to the amount of **M**_**3**_^**Seed**^ added (Fig. 5b, inset), indicating that the chain-growth mechanism is preserved. The average length of fibers observed by AFM and the calculated PDI of **M**_**5**_**-M**_**3**_**-M**_**5**_ supramolecular polymers obtained with 3:1 and 5:1 [**M**_**5**_]/[**M**_**3**_^**Seed**^] ratios perfectly matched the results observed for the corresponding **M**_**3**_ homopolymer (Supplementary Fig. 35).

To demonstrate that chain fragmentation does not occur during copolymerization, which is a prerequisite for sequence control in the block copolymer, we performed a multicycle kinetic experiment (Fig. 5c)^70^. First, we prepared an **M**_**5**_**-M**_**3**_**-M**_**5**_ block copolymer using a [**M**_**5**_]/[**M**_**3**_^**Seed**^] ratio of 25:1 and followed the time-course of polymerization by CD spectroscopy. We then took half of the obtained solution and added an identical volume of **M**_**5**_ (1.2 mM, C_6_H_12_/CHCl_3_ 93:7 v/v) (Fig. 5a). As expected, the “living” supramolecular block copolymer resumed elongation after the addition of fresh monomer. To be able to draw conclusions from the reaction kinetics, we repeated this combined monomer addition and dilution experiment for three more cycles. We found that after each dilution cycle the rate of polymerization was reduced by half, which is consistent with the fact that the initial concentration of active termini **M**_**3**_^**Seed**^ was decreased by a factor of two after each dilution cycle. The initial slopes of the polymerization kinetics could be fitted according to the equation *y* = 0.7(1/2)^n−1^, where n is the number of cycles (Fig. 4d), providing a solid confirmation for the absence of fragmentation during LSP. Importantly, AFM analyses revealed elongation of **M**_**5**_**-M**_**3**_**-M**_**5**_ block copolymer upon addition of fresh feed of **M**_**3**_ further corroborating the “living” nature of supramolecular polymerization (Supplementary Figs. 40, 41). Finally, we were able to directly visualize supramolecular block copolymers by AFM. Height profiles along the fibers of **M**_**5**_**-M**_**3**_**-M**_**5**_ supramolecular copolymers revealed the presence of thinner middle parts with z-displacement of ca. 3.0–3.4 nm (Fig. 5f, g), which is in excellent agreement for the observed fiber width observed for the **M**_**3**_ homopolymer. The thicker termini, on the other hand, displayed a z-displacement of ca. 4.1–4.5 nm, which corresponds to the average fiber width observed for the **M**_**5**_ homopolymer (Supplementary Figs. 42, 43).

### Structure of the fibers

Having demonstrated how LSP allows us to control the length of well-defined fibers, we set out to understand the structure of these self-assemblies. In AFM images of **M**_**3**_^**Polymer**^ (Fig. 4b, g, h, i) we observed individual fibers with a height profile of ca. 3.1 nm. The diameter of the fibers is therefore significantly larger than the calculated length of monomer **M**_**3**_ (ca. 2.3 nm), suggesting that several one-dimensional filaments combine to form the final structure. In the ATR-IR spectrum of **M**_**3**_^**Polymer**^, we observed characteristic stretching frequencies for hydrogen-bonded amides at 3302 cm^−1^ and 1633 cm^−1^ as well as an intense C − F stretching band at 1018 cm^−1^, that is not present in non-polymerizable **MeM**_**3**_ (Supplementary Figs. 44, 45). Analysis of the crystal structure of precursor compound **2** revealed that in the solid state this compound class stacks via dipole-dipole interactions between all-cis C_6_H_6_F_5_ groups, and hydrogen bonding between carbamates (Supplementary Fig. 57). Most importantly, the crystal packing features pairs of neighboring stacks, which are arranged in an antiparallel fashion, which presumably serves to cancel the large macrodipoles. Taken together, we conclude from this data that the polymer contains separate stacks of the self-complementary all-cis C_6_H_6_F_5_ (dipole-dipole) and amide (H bonding) motifs.

**Fig. 6:**
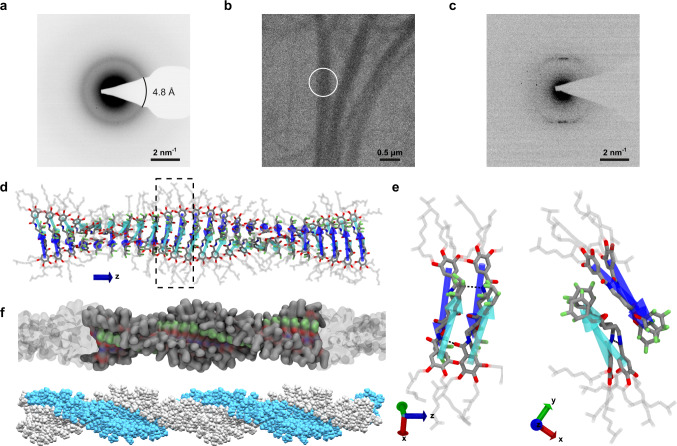
Structure of the fibers. **a** Electron diffraction pattern of the organic fibers under low-dose conditions (electron dose: 0.45 e^−^/Å^2^). The diameter of the illuminated area was c.a. 5 μm. The diffraction ring is located at 2.1 nm^−1^, corresponding to a lattice spacing of 4.8 Å. **b** Bright-field TEM image showing several bundles of the organic fibers. The circle indicates the position of the selected-area aperture with a diameter of 0.7 μm. **c** Selected-area electron diffraction pattern acquired from the circular region in (**b**) with an electron dose of 0.12 e^−^/Å^2^. **d** Atomistic detail of the monomer arrangement in the helix structure—blue and cyan arrows connecting the benzene (tail) to the pentafluorocyclohexyl groups (head). Color code: green: F; red: O; blue: N; gray: C. **e** Details of the side (left) and the top (right) views of the arrangement of four **M**_**3**_ in the helix (monomers within the black rectangle in **d**). H-bonds between the monomers are shown as dashed black lines. **f** Top: equilibrated snapshot of a **M**_**3**_ pre-assembled fiber after 1 μs of MD simulation. The system corresponds to a full helical turn with a pitch of ~12 nm (opaque), forming an infinite helicoidal fiber through the periodic boundaries (periodic images shown in transparency). Bottom: space-filling model of **M**_**3**_ polymer demonstrating relative arrangement of two monomer stacks (gray and blue).

To probe the crystallinity of the organic fibers, we performed electron diffraction using transmission electron microscopy (TEM) on a circular region with a diameter of ca. 5 μm. A diffraction ring is clearly visible (Fig. 6a), unambiguously demonstrating the long-range order within the organic fibers. The radial-integrated intensity profile of the diffraction pattern (Supplementary Fig. 46) reveals a lattice distance of 4.8 Å. By correlating the fiber morphology (Fig. 6b) with the selected-area electron diffraction pattern (Fig. 6c), we found that the direction of the Bragg reflections coincides with the long axis of the organic fibers; the molecular stacking direction is parallel to the fiber elongation direction, leading to the significant size anisotropy of the organic fibers. The too high sensitivity toward electron irradiation precluded further structural elucidation via high-resolution TEM imaging (Supplementary Fig. 47).

As experimentally observed, the formation of fibers is a complex process, which is challenging to investigate in silico at the molecular level. By running MD simulations of a molecular system containing 150 initially disassembled **M**_**3**_ monomers, we could observe that the free energy barrier for **M**_**3**_ folding-unfolding can be effectively crossed (unfolding), while this event may be facilitated when several monomers interact non-covalently. This indicates that the monomers can efficiently explore the previously discussed FES (Fig. 3d) during self-assembly (Supplementary Fig. 48a). Although the formation of persistent ordered assemblies could not be observed during classical MD simulations, we could obtain important qualitative insights into the key monomer-monomer interactions during the polymerization process. Especially, the inter-monomer H-bonding between amide-carbonyl groups and the stacking of the pentafluorocyclohexanes emerge as dominant interactions (Supplementary Fig. 48b). In order to study the assembly of (unfolded) **M**_**3**_ into fibers, we tested two possible self-assembled configurations, where the **M**_**3**_ monomers are respectively arranged into stacks in parallel or antiparallel fashion. We then compared these fibers’ structures from the point of view of their dynamic stability. A parallel axial **M**_**3**_ stacking is stabilized by the H-bonds between the monomers and the dipole-dipole interactions between the pentafluorocyclohexane dipoles. However, the solvophilic properties of such a parallel stack are not well balanced, because the resulting assembly has an implicit amphiphilic character, with all solvophilic tails oriented on one side and all solvophobic heads on the other side. An antiparallel axial **M**_**3**_ stacking still allows for a favorable hydrogen-bonding between the monomers while at the same time it guarantees a more uniform solvophilicity of the fiber. In both cases, single filaments were found unstable in the solvent during MD simulations. This suggested that higher scale self-assembly of these into hierarchical fibers composed of multiple filaments is a likely event, consistent with experimental evidence obtained by AFM (vide supra).

We investigated various possible hierarchical arrangements of **M**_**3**_ stacks. The configuration that demonstrated the highest stability is that reported in Fig. 6, where two antiparallel **M**_**3**_ stacks (blue and cyan arrows in Fig. 6d) combine to form a helical superstructure. This proposed structure for the assembly is stabilized via H-bonds between the amides and stacking of pentafluorocyclohexane dipoles (Fig. 6e), while at the same time providing an optimized interaction with the solvent (and conceptually the cancellation of the two macrodipole moments). Preformed antiparallel **M**_**3**_ stacks were seen to torque spontaneously during MD simulations in the solvent. Deeper modeling studies demonstrated that the most favorable tilting angle corresponds to a helical pitch of ~10–15 nm. Such **M**_**3**_ helices were found stable for over 1 μs of MD simulation (Supplementary Fig. 49b–d). The equilibrated helix structure has an average diameter consistent with that measured experimentally (Supplementary Fig. 50: ~3.1 nm). Within the equilibrated helix, the stacking distance between the antiparallel **M**_**3**_ dimers is ~5 Å, which is in very good agreement with the ~4.8 Å determined experimentally (Fig. 6a, c).

**Fig. 7:**
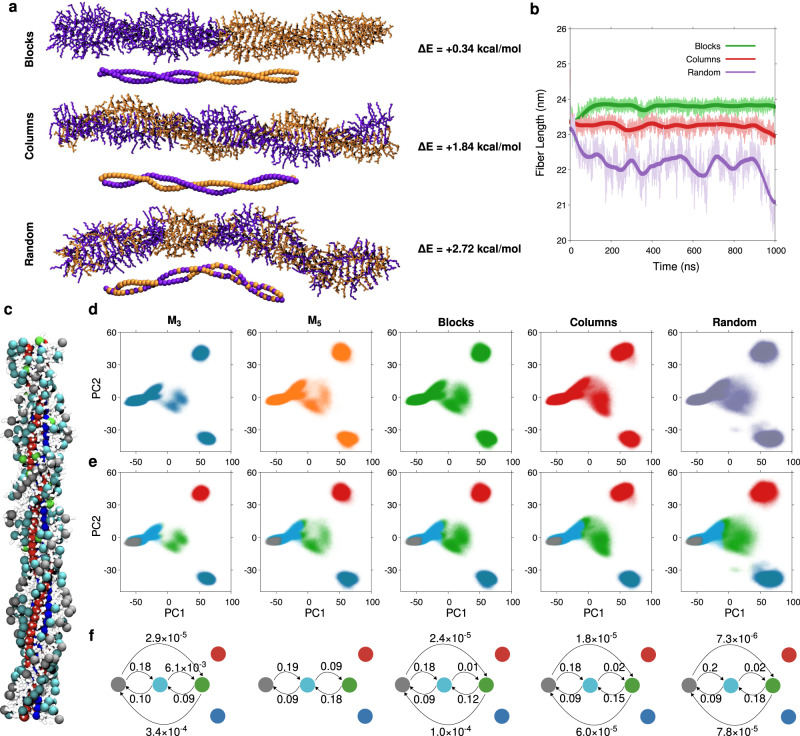
Structure and dynamics of supramolecular block copolymers. **a** Equilibrated MD snapshots of **M**_**3**_-**M**_**5**_ copolymers models where the **M**_**3**_ (in orange) and **M**_**5**_ monomers (violet) are arranged in blocks (top), into segregated **M**_**3**_ and **M**_**5**_ intertwining columns (middle), or randomly mixed in the fiber (bottom). The arrangements of the fluorinated cyclohexane centers are shown below the MD fibers’ snapshots. The energies of the different **M**_**3**_-**M**_**5**_ mixing schemes relative to completely segregated **M**_**3**_ and **M**_**5**_ homopolymers (∆E) are reported beside each equilibrated copolymer model. **b** Length of the Blocks (in green), Columns (red) and Random (violet) copolymer models as a function of MD simulation time. **c** Structure of the Blocks fiber colored based on the clusters (molecular motifs) identified by the SOAP-PAMM analysis: fluorinated cyclohexane and amide groups in blue and red, the alkyl side chains of the monomers in gray, green and cyan^66,67^. **d** PCAs of the SOAP vectors for **M**_**3**_ and **M**_**5**_ homopolymers, and for Blocks, Columns and Random copolymers (left-to-right). **e** Unsupervised PAMM clustering of the PCA data (**d**): different colored clusters indicate different local structural/dynamic motifs in the fibers. The closer and more interconnected are the colors (e.g., gray, cyan, green surface sites), the more dynamic is the interconnection between them (e.g., indicative of a dynamic surface). Separated clusters identify static, ordered, and persistent domains in the fiber (e.g., red and blue clusters, indicative of a relatively static fiber backbone). **f** Schematic representation of the dynamic transitions between the clusters estimated from the MD: the higher are the transition probabilities (numbers associated to the transition arrows), the more dynamic is the exchange between the clusters. No arrows indicate no exchange. The results show that these fibers have an intrinsic dynamic diversity: with a dynamic surface, and a static and ordered backbone.

Starting from our **M**_**3**_ fiber model, we conducted an analogous investigation on a **M**_**5**_ homopolymer model (Supplementary Fig. 51). Also in this case, the **M**_**5**_ fiber model showed high stability during 1 μs of MD simulation. The equilibrated **M**_**5**_ fiber model showed a helical pitch of ~10–15 nm, and overall similar structure to that of **M**_**3**_ fiber. Next, we built **M**_**3**_-**M**_**5**_ copolymer models that we compared between them, and with the **M**_**3**_ and **M**_**5**_ homopolymers. In particular, we compared three copolymer models starting from a helical conformation with 12 nm of pitch, where 48 monomers of **M**_**3**_ and 48 monomers of **M**_**5**_ are arranged into segregated Blocks (Fig. 7a, top), into segregated intertwining Columns (Fig. 7a, middle), or are distributed in Random fashion in the copolymer (Fig. 7a, bottom). All three copolymer models were pre-equilibrated and simulated via 1 μs of MD. We calculated the monomer-monomer interaction energies between the monomers in the copolymer structures, and compared these with the energy of the same number of **M**_**3**_ and **M**_**5**_ monomers separated into two **M**_**3**_ and **M**_**5**_ homopolymers. While ΔE > 0 values indicate that from an energetic point of view the intermixing of monomers is unfavored, such event is favored entropically in the real system. In particular, monomer mixing in Blocks configuration shows a very similar energy than two separated **M**_**3**_ and **M**_**5**_ homopolymers (ΔE = + 0.34 kcal/mol), while mixing them in Columns or Random fashion appears to be more energetically unfavorable (ΔE of + 1.84 and +2.72 kcal/mol respectively). However, it is worth noting that these ΔE values (expressed per **M**_**3**_-**M**_**5**_ couple) are quite small. This suggests that in this case the entropic tendency to **M**_**3**_-**M**_**5**_ mixing is dominant during self-assembly, and that in the real systems the monomers likely intermix in the fibers in a more/less blocky/random fashion. On the contrary, a complete segregation in separated **M**_**3**_ and **M**_**5**_ homopolymers, as well as a homogeneous 1:1 **M**_**3**_: **M**_**5**_ intermixing in the fibers appear to be unfavorable from the entropic point of view.

From a structural point of view, comparing the behavior of the fibers during the MD runs we observed that the Blocks model is more persistent than the Columns and Random models. Shown in Fig. 7b, the Blocks preserves the starting fiber length (green), while the random fiber evolves toward a more distorted/bent conformation (purple). To gain a deeper knowledge on the internal structural dynamics of these assemblies, we used a high-dimensional analysis based on the Smooth Overlap of Atomic Position (SOAP) vectors^71^, as agnostic fingerprints capable of capturing structural and dynamic differences/similarities in the atomic environments in supramolecular polymers^72,73^ and soft complex molecular systems^74^. We use a set of five SOAP centers in the **M**_**3**_ and **M**_**5**_ monomers in the fiber models (Supplementary Fig. 52), one in the center of the fluorinated ring, one in the amide, and three in the terminal alkyl groups of the monomers (details in Supplementary Information). The SOAP analysis allowed us to classify the local environments that surround these sites based on their local structural and dynamic features. We use a Principal Component Analysis (PCA) to reduce the dimensionality of the SOAP vectors (Fig. 7d: PCA projections along the first two PCA components, PC1 and PC2, for all simulated systems). In particular, the first three components of the PCA retain up to 86% of the system fluctuations/complexity, and were used for the analyses.). We used the Probabilistic Analysis of Molecular Motifs (PAMM)^72,75^ unsupervised clustering method to identify the dominant macroclusters in the systems: different colors in the PCAs of Fig. 7e indicate structurally/dynamically different domains/motifs within the assembly as identified by the PAMM-SOAP analysis. By coloring the beads accounted in the SOAP analysis based on the clusters to which these belong (Fig. 7e), we can observe that the red and blue colored clusters correspond to the cyclohexane ring and to the amide respectively (see also Fig. 7c). The other three clusters (Fig. 7e: gray, cyan and green) correspond to the tails of the monomers, and to the surface of the fibers (see Fig. 7c). Performing the SOAP-PAMM analysis on the second half of the MD trajectories sampled every 100 ps allows us monitor the transitions of the sites between different clusters (dynamic change of colors). From the frequency of these transitions, we can then estimate transition probabilities (Fig. 7f: which divided by the time-lapse between the analyzed MD snapshots, provide the transition rates), which are indicative of the dynamics of the different domains within the fibers. This analysis demonstrates how in the time explored by the MD simulations the core of the fibers is substantially static compared to the fibers’ surface. In fact, the absence of arrows between the red and blue macroclusters of Fig. 7f indicates that the domains of the stacked fluorinated cyclohexanes and of the amide groups is very persistent and quite static. On the other hand, the dynamic interconnection between gray, cyan and green clusters (Fig. 7f) shows that in the same time-lapse there is dynamics on the surface of these fibers. The rich dynamic nature of these fibers is reminiscent of the innate dynamic diversity seen for also in other supramolecular polymers in solution^72,76^. Also, the growing interconnection between gray, cyan and green clusters going from left-to-right in Fig. 7f indicate that the surface of the fibers becomes more dynamic and disordered going from homopolymers to increased **M**_**3**_- **M**_**5**_ mixing within the copolymers.

In conclusion, we describe a new type of building block for supramolecular polymerization. We were able to demonstrate that monomers comprising facially polarized fluorocyclohexanes engage in intramolecular hydrogen bonding, dipole-dipole and CH···π interactions, leading to dormant states, which enable seeded LSP. We were able to make use of this kinetically controlled process to prepare self-assembled nanofibers of controlled length as well as supramolecular A-B-A block copolymers. To the best of our knowledge, this is the first report on supramolecular block copolymers synthesized from kinetically trapped monomers rather than off-pathway aggregates. The monomers reported herein differ from the state-of-the art in LSP by their simplicity, the lack of extended π-systems or metal centres, and the facile variation of side chains. For this reason, we envisage that fluorinated cyclohexanes may play a vital role in progress on LSP. Future work will focus on the synthesis of more complex block copolymers and an exploration of ferroelectric properties^77^.

## Supplementary information

Supplementary Information

## Data Availability

All data generated and analyzed during this study are included in this article and its Supplementary Information files. Crystallographic data have been deposited at the Cambridge Crystallographic Data Centre (CCDC) as CCDC 2010482 and CCDC 2071404 and can be obtained free of charge from the CCDC via https://www.ccdc.cam.ac.uk/.
