# Supplementary material for: Living supramolecular polymerization of fluorinated cyclohexanes
Source: Nat Commun. 2021 May 25;12:3134. doi: 10.1038/s41467-021-23370-y (PMC8149861; doi:10.1038/s41467-021-23370-y)
Supplement: Supplementary file 1 — Supplementary Information [file 41467_2021_23370_MOESM1_ESM.pdf]

Supporting Information for

Living Supramolecular Polymerization of Fluorinated Cyclohexanes

Oleksandr Shyshov[1], Shyamkumar Vadakket Haridas[1], Luca Pesce[2], Haoyuan Qi[3,4], Andrea Gardin[6], Davide Bochicchio[2,5], Ute Kaiser[3], Giovanni M. Pavan[2,6]* & Max von Delius[1]*

[1]Institute of Organic Chemistry, University of Ulm, Albert-Einstein-Allee 11, 89081 Ulm, Germany. Email: max.vondelius@uni-ulm.de

[2]Department of Innovative Technologies, University of Applied Sciences and Arts of Southern Switzerland, Polo Universitario Lugano - Campus Est, Via la Santa 1, CH-6962 Lugano - Viganello, Switzerland

[3]Central Facility of Electron Microscopy, Electron Microscopy Group of Materials Science, University of Ulm, 89081 Ulm, Germany.

[4]Center for Advancing Electronics Dresden (cfaed) and Faculty of Chemistry and Food Chemistry, Technical University of Dresden, 01069 Dresden, Germany.

[5]Department of Physics, Università degli studi di Genova, Via Dodecaneso 33, 16100 Genova, Italy.

[6]Department of Applied Science and Techology, Politecnico di Torino, Corso Duca degli Abruzzi 24, 10129 Torino, Italy. Email: giovanni.pavan@polito.it

Supplementary Methods

General experimental methods

All commercially available reagents were purchased from Sigma Aldrich, Alfa Aesar, Acros Organics, ABCR or TCI and were used without further purification unless otherwise stated. Hydrogenation reactions were carried out in a 150mL Roth HighPressure Autoclave. NMR spectra were recorded on a BrukerAvance 400 (^{1}H: 400 MHz; ^{13}C: 101 MHz; ^{19}F: 377 MHz), a BrukerAvance 500 (^{1}H: 500 MHz; ^{13}C: 125 MHz) spectrometers at 295 K and referenced to the residual solvent peak (^{1}H: chloroform-*d*, 7.26 ppm; acetone-d_6, 2.05 ppm; DMSO-d_6, 2.5 ppm; cyclohexane-d_{12}, 1.38 ppm; ^{13}C: chloroform-*d*, 77.00 ppm; acetone-d_6, 206.68 ppm, DMSO-d_6, 39.52 ppm). Coupling constants (J) are denoted in Hz and chemical shifts (δ) in ppm. Multiplicities are denoted as follows: s = singlet, d = doublet, t = triplet, m = multiplet, br = broad. Mass spectra were obtained on a Bruker SolariX (HRMS-ESI^{+}, solvent: acetonitrile) instrument. CD spectra were measured in a 1 mm quartz cuvette on Jasco J-810 CD spectrometer equipped with Julabo F12 temperature controller. A Bandelin Sonorex was used as an “ultrasonic bath” (US bath). Single-crystal diffraction analysis data was collected at 150 K with a Rigaku/Oxford instruments Supernova. Atomic force microscopy (AFM) was performed on a NanoScope IIIa device (Veeco Instruments, Santa Barbara, CA) in tapping mode under air. Commercial antimony doped silicon tips (Type RTESPA-150) were used with a nominal tip radius of 8 nm and a spring constant of 6 N m^{-1} at a resonance frequency of 150 kHz. Transmission Fourier transform infrared spectroscopic analyses (FT-IR) was performed on a Bruker Tensor II IR spectrometer equipped with transmission compartment using a liquid cell with KBr windows and 1 mm path length. ATR-IR analyses was performed using Bruker Alpha II spectrometer. TEM experiments were performed on an image-side aberration-corrected FEI Titan 80-300 operated at 300kV. The microscope is equipped with a CEOS hexapole aberration-corrector , which corrects the geometrical axial aberrations up to the 3rd-order. Data acquisition was conducted on a Gatan UltraScan CCD camera. Electron diffraction was conducted under both nano-beam diffraction mode and selected-area electron diffraction mode. Due to the high beam sensitivity of the organic fibers, we applied low-dose conditions during data acquisition, and the electron dose was limited in the range of 0.1 - 0.5 e^{-}/Å^{2}. For sample preparation, the organic fibers, obtained using a **[M$_3$]**/[**M$_3^{Seed}$**] 100/1 (v/v) ratio, were 100x time diluted with cyclohexane. Subsequently, 10 μL of the diluted solution was drop-casted on to a holey carbon TEM grid. The grid was left in ambiance for c.a. 1 h before TEM investigation. Dynamic light scattering (DLS) was performed using Malvern Instruments Zetasizer Nano ZS, UK at 293 K with a scattering angle 173° and wavelength λ = 633 nm.

Procedure for M_3^{Seed} preparation:

M_3^{Seed} solution was obtained by applying sonication to a Cyclohexane/$CHCl_3$ (84:16 v/v) solution of **M_3** and ***Me*M3** (molar ratio: 3:1; total concentration: 1.2 mM) for 20 minutes at 273 K in a round-bottom flask tightly closed with glass stopcock. **M_3^{Seed}** was used for further experiments immediately.

General procedure for seeded LSP

A 1.2 mM solution of **M_3** in 84:16 v/v cyclohexane/chloroform in was added to a 1 mm quartz cuvette and annealed for 40 sec at 323 – 328 K to ensure complete depolymerisation. After this treatment spontaneous polymerization does not occur for >3 h. The monomer solution was placed into a CD spectropolarimeter with the temperature set to 293 K and thermostated for 10 min. A defined volume of an **M_3^{Seed}** solution was added and the cuvette content was homogenized by turning the cuvette upside down 5 – 10 times and placed back into the CD spectropolarimeter at 293 K.

Notes: 1) For every experiment we prepared fresh **M_3^{Seed}** solutions (old solutions can be depolymerized as described above); 2) **M_3^{Seed}** solution was always kept at 273 K.

AFM studies on supramolecular polymers

Samples of supramolecular polymers for AFM were prepared by spin-coating (8000 rpm) on the silicon wafer immediately after 100-fold dilution with cyclohexane. Dilution is necessary to prevent severe bundling. AFM images were recorded for several different areas of the substrate. AFM data was processed using WSxM 5.0 software.[1] Images were analyzed using the ImageJ software, developed at the US National Institute of Health.[2] Lengths of all separate unbundled fibers were used for statistics. In every case, we measured approximately 200 fibers (Supplementary Table 1). Weight-averaged length (L_w) and number-averaged length (L_n) for n objects were calculated according to equations S1 and S2.

$$L_w = \frac{\sum_{i=1}^{n} N_i L_i^2}{\sum_{i=1}^{n} N_i L_i} \qquad \text{(S1)}$$

$$L_n = \frac{\sum_{i=1}^{n} N_i L_i}{\sum_{i=1}^{n} N_i} \qquad \text{(S2)}$$

The length distribution was characterized by the polydispersity index (PDI) according to:

$$PDI = \frac{L_w}{L_n} \qquad \text{(S3)}$$

General procedure for preparation of supramolecular block copolymer by seeded LSP

A 1.2 mM solution of **M**$_5$ in 93:7 v/v cyclohexane/chloroform was added to a 1 mm quartz cuvette and was annealed for 40 sec at 323 – 328 K to ensure complete depolymerisation. The monomer solution was placed in a CD spectropolarimeter with temperature set to 293 K and thermostated for 10 min. A defined volume of **M**$_3^{\mathbf{Seed}}$ solution was added and the cuvette content was homogenized by turning the cuvette upside down 5 – 10 times and placed back into CD spectropolarimeter at 293 K.

Notes: 1) For every experiment we prepared fresh **M**$_3^{\mathbf{Seed}}$ solutions (old solutions can be depolymerized as described above); 2) **M**$_3^{\mathbf{Seed}}$ solution was always kept at 273 K.

Living supramolecular polymerization

To provide evidence for the living nature of supramolecular polymerization we performed a multicycle dilution experiment. 20 µL of **M**$_3^{\mathbf{Seed}}$ solution was added to 200 µL of **M**$_3$ solution at 293 K, polymerization occurred and was completed within 60 seconds. 100 µL of the obtained polymer solution was added to 100 µL of **M**$_3$ solution. This procedure was repeated for two more cycles (Supplementary Fig. 36a). Since after each cycle, the initial concentration of **M**$_3^{\mathbf{Seed}}$ was diluted by half, the rate of polymerization decreases by half as well (Supplementary Fig. 36b). The rates of polymerization can be fitted according to exponential equation $y = A*(1/2)^{x-1}$, where x is the number of cycles (Supplementary Fig. 36c). These results clearly indicate that the number of active termini does not change during polymerization.

To demonstrate the living nature of supramolecular block copolymers, to 1 volume of **M**$_5$-**M**$_3$-**M**$_5$ **(**obtained with [**M**$_5$]/[**M**$_3^{\mathbf{Seed}}$] = 3:1 (v/v) ratio) block copolymer we added 0.5 volumes of **M**$_3$ (1.2 mM, 84:16 cyclohexane/chloroform v/v, 293 K) and investigate length distribution using AFM (Supplementary Fig. 40b).

Thermodynamics of polymerization

To obtain thermodynamic data, we studied thermal depolymerisation of polymers, which proceeds under thermodynamic control unlike the kinetically controlled, cooling-induced polymerization. The heating curves of the polymers, obtained upon cooling of the polymer solution, have unusual kinks presumably due to fast kinetics of polymerization leading to defects in the molecular packing and presence of different polymorphs in the same sample (for example those that form during spontaneous polymerization). Therefore, we prepared polymers via seeded growth under kinetic control, using different initial concentrations of the monomer and constant monomer/seed ratio ([**M**$_3$]/[**M**$_3^{\mathbf{Seed}}$]

50/1). The obtained polymer solutions were cooled down to 275 K and heating CD curves were recorded (Supplementary Fig. 53).

Obtained heating curves had non-sigmoidal shapes with clear transitions at T_e. The molar fraction of aggregated molecules (α_{agg}) was calculated according to the equation S4, where CD_{agg} and CD_{mono} are CD intensities of fully aggregated (at the lowest temperature) and monomeric states (at the highest temperature). The plot of α_{agg} versus temperature (Supplementary Fig. 54) provides heating curves which can be fitted according to the cooperative model proposed by Meijer and co-workers (equation S5) in which h_e is the molecular enthalpy release during elongation, T_e and T are elongation temperature and absolute temperature respectively.[3]

$$\alpha_{agg} = 1 - \frac{CD_{agg} - CD(T)}{CD_{agg} - CD_{mono}} \qquad \text{(S4)}$$

$$\alpha = \alpha_{SAT}\left[1 - exp\left(\frac{-h_e}{RT_e^2}(T - T_e)\right)\right] \qquad \text{(S5)}$$

The standard values of enthalpy ($\Delta H°$), entropy ($\Delta S°$) and Gibbs free energy were obtained using the van`t Hoff plot (Supplementary Fig. 55).

Synthesis and characterization data

All-*cis* 2,3,4,5,6-pentafluorocyclohexan-1-ol (1)

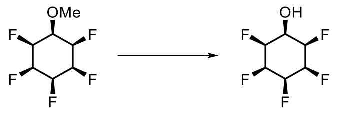

A 25 mL Schlenk flask was charged with all-cis 1,2,3,4,5-pentafluoro-6-methoxycyclohexane[4] (168 mg, 0.83 mmol, 1 equiv.) and 8 mL of anhydrous CH_2Cl_2. Butane-1-thiol (0.75 g, 0.89 mL, 8.31 mmol, 10 equiv.) and anhydrous $AlCl_3$ (406 mg, 2.91 mmol, 3.5 equiv.) were added subsequently and the reaction mixture was stirred for 14 hours at room temperature. The reaction mixture was filtered through silica pad, the first fraction was washed with hexane to remove the thiol, second with acetone. The acetone fraction was concentrated and the crude material was purified by flash column chromatography (CH_2Cl_2/acetone 8:2) to give **1** (129 mg, 83%) as a colourless solid.

^{1}H NMR (400 MHz, acetone-*d*$_6$, 295 K): δ 5.51 - 4.77 (m, 5H), 3.98 (t, 1H).

^{19}F NMR (377 MHz, acetone-*d*$_6$, 295 K): δ -211.20 (br s, 2F), -217.5 (m, 2F), -217.63 (m, 1F).

^{13}C NMR (101 MHz, acetone-d_6, 295 K): δ 91.70 (m), 89.73(m), 87.52 (m), 85.63 (m), 66.82 (m).

HRMS (ESI$^-$): *m/z* = 248.9728 $[M+CH_3COO]^-$ (calculated 249.0556 for $C_8H_{10}F_5O_3^-$).

General procedure compounds S1 – S3

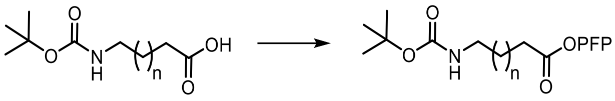

S1, n = 0
S2, n = 1
S3, n = 2

In a 100 mL round-bottom flask corresponding Boc-aminoacid (25 mmol, 1 equiv.), 2,3,4,5,6-pentafluorophenol (28 mmol, 1.12 equiv.) and few crystals of 4-dimethylaminopyridine were dissolved in 30 mL of CH_2Cl_2. EDC (28 mmol, 1.12 equiv.) was added and the reaction mixture was stirred 12h at room temperature. The reaction mixture was diluted with 70 mL of CH_2Cl_2 and washed with water (4*30 mL) and brine (30mL). The organic phase was dried over magnesium sulfate and evaporated to afford the target compounds, which were used for the next step without purification.

Perfluorophenyl 3-((tert-butoxycarbonyl)amino)propanoate (S1)

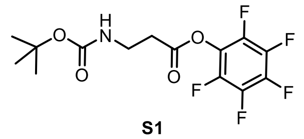

S1

Crude yield: 100%, brownish solid.

^{1}H NMR (400 MHz, $CDCl_3$, 295 K): δ 5.05 (br, 1H), 3.50 (q, J = 6.2 Hz, 2H), 2.91 (t, J = 6.1 Hz, 2H), 1.43 (s, 9H).

^{19}F NMR (377 MHz, $CDCl_3$, 295 K): δ -151.58 – -152.93 (m, 2F), -157.86 (t, J = 21.6 Hz, 2F), -162.03 – -162.68 (m, 2F).

^{13}C NMR (101 MHz, $CDCl_3$, 295 K): δ 168.34, 155.84, 142.44 – 142.06 (m), 141.21 – 140.40 (m), 140.36 – 139.56 (m), 139.27 – 138.9 (m), 138.65 – 137.96 (m), 137.04 – 136.34 (m), 79.85, 35.94, 33.83, 28.20.

HRMS (ESI$^+$): *m/z* = 378.0727 $[M+Na]^+$ (calculated 378.0741 for $C_{14}H_{14}F_5NO_4Na^+$).

Perfluorophenyl 4-((tert-butoxycarbonyl)amino)butanoate (S2)

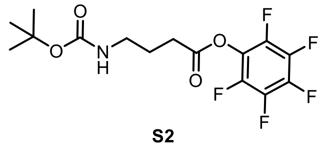

S2

Crude yield: 98%, white solid.

^{1}H NMR (400 MHz, $CDCl_3$, 295 K): δ 4.67 (br, 1H), 3.25 (q, J = 6.6 Hz, 2H), 2.72 (t, J = 7.4 Hz, 2H), 1.96 (p, J = 7.0 Hz, 2H), 1.44 (s, 9H).

^{19}F NMR (377 MHz, $CDCl_3$, 295 K): δ -151.03 – -154.44 (m, 2F), -157.64 (dt, J = 33.6, 15.1 Hz, 2F), -161.98 (q, J = 20.6, 17.3 Hz, 1F).

^{13}C NMR (101 MHz, $CDCl_3$, 295 K): δ 169.30, 156.20, 142.63 – 141.99 (m), 140.95 – 140.41 (m), 140.04 – 139.59 (m), 139.37 – 138.78 (m), 138.39 – 137.96 (m), 136.85 – 136.24 (m), 77.36, 39.70, 30.72, 28.49, 25.46.

HRMS (ESI^+): *m/z* = 392.0884 $[M+Na]^+$ (calculated 392.0897 for $C_{15}H_{16}F_5NO_4Na^+$).

Perfluorophenyl 5-((tert-butoxycarbonyl)amino)pentanoate (S3)

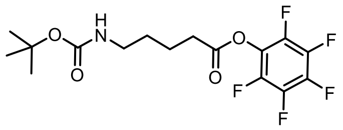

S3

Crude yield: 100%, brown solid.

^{1}H NMR (400 MHz, $CDCl_3$, 295 K): δ 4.57 (br, 1H), 3.18 (q, J = 6.7 Hz, 2H), 2.70 (t, J = 7.3 Hz, 2H), 1.80 (tt, J = 7.5 Hz, 6.2 Hz, 2H), 1.61 (dq, J = 10.4 Hz, 7.1 Hz, 2H) 1.44 (s, 9H).

^{19}F NMR (377 MHz, $CDCl_3$, 295 K): δ -152.75 (dd, J = 21.9 Hz, 4.9 Hz, 2F), -158 (t, J = 21.6 Hz, 1F), -162.29 (q, J = 22.9, 18.1 Hz, 1F).

^{13}C NMR (101 MHz, $CDCl_3$, 295 K): δ 169.25, 156.03, 142.63 – 142.02 (m), 141.03 – 139.55 (m), 139.53 – 137.57 (m), 137.04 – 136.25 (m), 79.32, 39.87, 32.81, 29.27, 28.34, 21.84.

HRMS (ESI^+): *m/z* = 406.1055 $[M+Na]^+$ (calculated 406.1054 for $C_{16}H_{18}F_5NO_4Na^+$).

All-*cis* 2,3,4,5,6-pentafluorocyclohexyl acetate (Ref2)

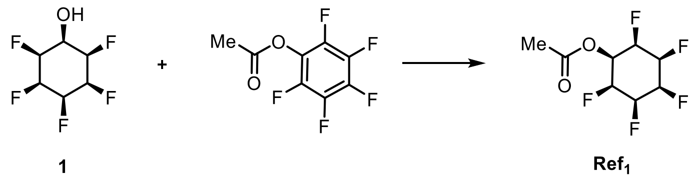

In a 10 mL round-bottom flask pentafluorophenyl acetate (150 mg, 0.66 mmol, 2.5 equiv.) and **1** (50 mg, 0.26 mmol, 1 equiv.) were dissolved in 1 mL of dry DMF and 1 mL of triethylamine. Reaction mixture was stirred at 120 °C until **1** was consumed (ca. 2 – 3 hours). Solvents were removed under reduced pressure and the crude material was purified by flash column chromatography (gradient elution CH_2Cl_2 to CH_2Cl_2/acetone 8:2) to afford all-cis 2,3,4,5,6-pentafluorocyclohexyl acetate (56 mg, 92%) as a colourless crystalline solid.

^{1}H NMR (400 MHz, acetone-d_6, 295 K): δ 5.60 – 4.86 (m, 6H), 2.15 (s, 3H).

^{19}F NMR (377 MHz, acetone-d_6, 295 K): δ -211.48 (br s, 2F), -215.92 (br s, 2F), -217.86 (br s, 1F).

^{13}C NMR (101 MHz, acetone-d_6, 295 K): δ 170.55, 90.15 – 88.28 (m), 87.95 – 86.28 (m), 86.9 – 84.51 (m), 68.10 – 67.25 (m), 20.91.

HRMS (ESI$^-$): *m/z* = 267.0216 [M + Cl]$^-$ (calculated 267.0217 for $C_8H_{10}F_5O_3Cl^-$).

General procedure compounds 2 - 4

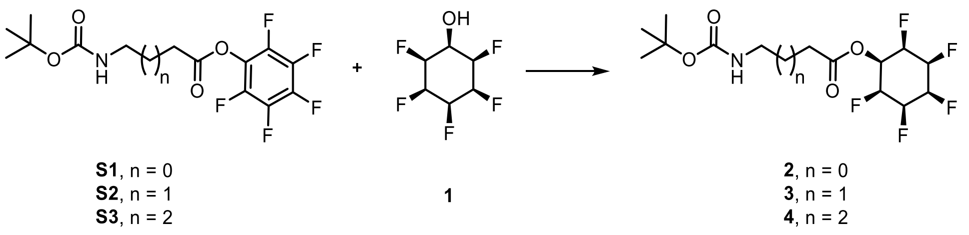

In a 10 mL round-bottom flask pentafluorophenyl ester **S1** – **S3** (7.7 mmol, 1.9 equiv.) and **1** (4 mmol, 1 equiv.) were dissolved in 3 mL of dry DMF and 2 mL of triethylamine. The reaction mixture was stirred at 120 °C until **1** was completely consumed (ca. 2 - 3 hours). Solvents were removed under reduced pressure and the crude material was purified by flash column chromatography (gradient elution CH_2Cl_2 to CH_2Cl_2/acetone 8:2).

All-*cis* 2,3,4,5,6-pentafluorocyclohexyl 3-((tert-butoxycarbonyl)amino)propanoate (2)

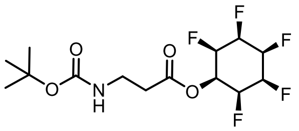

2

Yield: 95%, white solid.

^{1}H NMR (400 MHz, acetone-d_6, 295 K): δ 6.07 (br, 1H), 5.59 – 4.78 (m, 6H), 3.39 (q, J = 6.5 Hz, 2H), 2.66(t, J = 6.8 Hz, 2H), 1.39 (s, 9H).

^{19}F NMR (377 MHz, acetone-d_6, 295 K): δ -211.47 (br s, 2F), -215.84 (br s, 2F), -217.81 (br s, 1F).

^{13}C NMR (101 MHz, acetone-d_6, 295 K): δ 171.81, 156.97, 89.88 – 88.62 (m), 88.30 – 86.26 (m), 86.17 – 84.46 (m), 79.31, 68.34 – 67.22 (m), 37.45, 35.68, 29.0.

HRMS (ESI$^+$): *m/z* = 400.0938 [M + K]$^+$ (calculated 400.0950 for $C_{14}H_{20}F_5NO_4K^+$).

All-*cis* 2,3,4,5,6-pentafluorocyclohexyl 4-((tert-butoxycarbonyl)amino)butanoate (3)

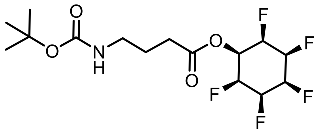

3

Yield: 100%, white solid.

^{1}H NMR (400 MHz, DMSO-d_6, 295 K): δ 6.88 (t, J = 5.8 Hz, 1H), 5.61 – 4.82 (m, 6H), 2.96 (q, J = 6.5 Hz, 2H), 2.48 – 2.38 (m, 2H), 1.66 (p, J = 7.2 Hz, 2H), 1.37 (s, 9H).

^{19}F NMR (377 MHz, DMSO-d_6, 295 K): δ -210.13 (br s, 2F), -214.63 (br s, 2F), -216.85 (br s, 1F).

^{13}C NMR (101 MHz, DMSO-d_6, 295 K): δ 177.61, 155.68, 88.42 – 87.00 (m), 86.55 – 84.89 (m), 84.29 – 82.92 (m), 77.55, 66.41 – 65.47 (m), 30.59, 28.25, 27.68, 24.80.

HRMS (ESI$^+$): *m/z* = 398.1371 [M + Na]$^+$ (calculated 398.1392 for $C_{15}H_{22}F_5NO_4Na^+$).

All-*cis* 2,3,4,5,6-pentafluorocyclohexyl 5-((tert-butoxycarbonyl)amino)pentanoate (4)

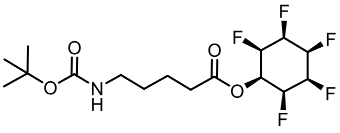

4

Yield: 92%, white solid.

^{1}H NMR (500 MHz, acetone-d_6, 295 K): δ 5.93 (br, 1H), 5.71 – 4.76 (m, 6H), 3.08 (t, J = 6.2 Hz, 2H), 2.49 (t, J = 7.4 Hz, 2H), 1.80 – 1.61 (m, 2H), 1.55 (p, J = 7.1 Hz, 2H), 1.39 (s, 9H).

^{19}**F NMR** (377 MHz, acetone-d_6, 295 K): δ -211.46 (br s, 2F), -215.92 (br s, 2F), -217.85 (br s, 1F).

^{13}**C NMR** (126 MHz, acetone-d_6, 295 K): δ 173.35, 157.22, 89.86 – 88.66 (m), 88.25 – 86.41 (m), 86.41 – 84.90 (m), 78.90, 68.27 – 67.38, 41.09, 40.96, 29.18, 23.28, 16.22.

HRMS (ESI$^+$): *m/z* = 412.1514 [M + Na]$^+$ (calculated 412.1523 for $C_{16}H_{24}F_5NO_4Na^+$).

Synthesis of S9

(S)-6-((tert-butyldimethylsilyl)oxy-4-methylhexanal (S4)

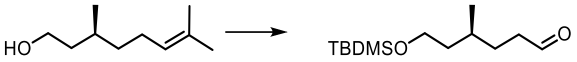

S4

A 250 mL round-bottom flask was charged with β-S-(-)citronellol (3 g, 19.2 mmol, 1 equiv.), 90 mL of dioxane and 30 ml of water. OsO_4 (96 mg, 0.38 mmol, 0.02 equiv.), $NaIO_4$ (16.4 g, 76.6 mmol, 4 equiv.) and 2,6-lutidine (4.38 ml, 4.1 g, 38 mmol, 2 equiv.) were added subsequently and the reaction mixture was stirred at room temperature. When all starting material was consumed, the reaction mixture was extracted with CH_2Cl_2. The organic phase was dried over magnesium sulfate, passed through a short celite pad and solvents were removed under reduced pressure. The brown oil, obtained after evaporation, was dissolved in 50 mL of CH_2Cl_2. TBDMSCl (4.3 g, 28 mmol, 1.5 equiv.) and imidazole (1.9 g, 28 mmol, 1.5 equiv.) was added and the reaction mixture was stirred for 12h at room temperature. The reaction mixture was transferred into a separatory funnel and washed with water (100 mL). The organic phase was dried over magnesium sulfate. The solvent was removed under reduced pressure and the residue was purified by flash column chromatography (petroleum ether/ethyl acetate 9:1) to give **S4** (2.6 g, 55% two steps) as a colourless oil.

^{1}**H NMR** (400 MHz, $CDCl_3$, 295 K): δ 9.77 (t, J = 1.9 Hz, 1H), 3.89 – 3.44 (m, 3H), 2.48 – 2.40 (m, 3H), 1.75 – 1.41 (m, 6H), 1.40 – 1.29 (m. 2H), 0.91 – 0.90 (m, 3H), 0.88 (s, 9H), 0.04 (s, 6H).

^{13}**C NMR** (101 MHz, $CDCl_3$, 295 K): δ 202.86, 61.04, 41.65, 39.49, 29.14, 28.93, 25.92, 19.33, 18.29, -5.32, -5.35.

HRMS (ESI$^+$): *m/z* = 245.1910 [M+H]$^+$ (calculated 245.1931 for $C_{13}H_{29}O_2Si^+$).

(S)-6-((tert-butyldimethylsilyl)oxy-4-methylhexan-1-ol (S5)

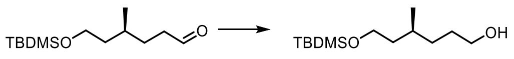

S4 S5

In a 250 mL round-bottom flask **S4** (2.6 g, 10.6 mmol, 1 equiv.) was dissolved in MeOH (100 mL) and $NaBH_4$ (401 mg, 10.6 mmol, 1 equiv.) was added at 0 °C. The reaction mixture was allowed to warm up to room temperature and was stirred at room temperature until all starting material was consumed. MeOH was evaporated under reduced pressure. The residue was dissolved in 100 mL of water and extracted with ethyl acetate (3*50 mL). The organic phase was washed with brine and dried over $MgSO_4$. The solvent was removed under reduced pressure and the crude material was purified by flash column chromatography (8:2 petroleum ether: ethyl acetate) to give **S5** (2.15 g, 82%) as a colourless oil.

^{1}H NMR (400 MHz, $CDCl_3$, 295 K): δ 3.71 – 3.55 (m, 4H), 1.68 – 1.43 (m, 5H), 1.43 – 1.28 (m, 2H), 1.23 – 1.12 (m, 1H), 0.93 – 0.85 (m, 12H), 0.04 (s, 6H).

^{13}C NMR (101 MHz, $CDCl_3$, 295 K): δ 63.29, 61.34, 39.81, 32.97, 30.19, 29.26, 25.94, 19.63, 18.31, -5.29, -5.31.

HRMS (ESI^+): *m/z* = 247.2090 $[M+H]^+$ (calculated 247.2093 for $C_{13}H_{31}SiO_2^+$).

(S)-tert-butyl((6-iodo-3-methylhexyl)oxy)dimethylsilane (S6)

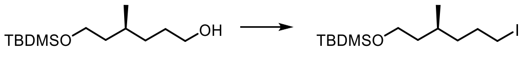

S5 S6

A 250 mL flask was charged with **S5** (2.15 g, 8.74 mmol, 1 equiv.) and dissolved in dry acetonitrile (50 mL). Triphenylphosphine (3 g, 11.4 mmol, 1.3 equiv.), iodine (3.1 g, 12.2 mmol, 1.4 equiv.) and imidazole (0.9 g, 13.1 mmol, 1.5 equiv.) were added subsequently under nitrogen atmosphere at 0 °C. The reaction mixture was stirred for 10 minutes at 0 °C and for 2 h at room temperature. The reaction mixture was concentrated under reduced pressure and 100 mL of petroleum ether was added. The precipitate was filtered off and the mother liquor was passed through a silica pad to obtain **S6** (1.96 g, 63%) as colourless oil.

^{1}H NMR (400 MHz, $CDCl_3$, 295 K): δ 3.70 – 3.58 (m, 2H), 3.21 – 3.11 (m, 2H), 1.94 – 1.73 (m, 2H), 1.65 – 1.49 (m, 2H), 1.46 – 1.29 (m, 2H), 1.29 – 1.17 (m, 1H), 0.93 – 0.83 (m, 12H), 0.04 (s, 6H).

^{13}C NMR (101 MHz, $CDCl_3$, 295 K): δ 61.16, 39.67, 37.87, 31.14, 28.70, 25.95, 25.86, 19.61, 18.30, 7.37, -5.27, -5.30.

HRMS (ESI^+): *m/z* = 357.1100 $[M+H]^+$ (calculated 357.1105 for $C_{13}H_{30}ISiO^+$).

(S)-tert-butyl((6-buthylthio)-3-methylhexyl)oxy)dimethylsilane (S7)

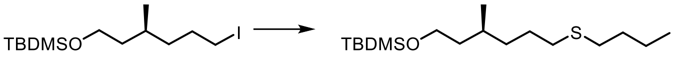

S6 **S7**

In a 100 ml round-bottom flask butane-1-thiol (594mg, 6.6 mmol, 1.2 equiv.) was dissolved in dry DMF (25 mL). Cs_2CO_3 (2.15g, 6.6 mmol, 1.2 equiv.), tetrabutylammonium iodide (2.4g, 6.6 mmol, 1.2 equiv.) were added and stirred for 1 h at room temperature under N_2 atmosphere. The reaction mixture was cooled to 0 °C and **S6** (1.96 g, 5.5 mmol, 1 equiv.) was added dropwise. The reaction mixture was stirred for 2 h at room temperature and water (100 mL) was added. The reaction mixture was extracted with ethylacetate (3x50 mL). The organic phase was washed with water, brine and dried over $MgSO_4$. The filtrate was concentrated under reduced pressure and the crude material was purified by flash column chromatography (petroleum ether/ethyl acetate 95:5) to give **S7** (1.4 g, 80%) as a colourless oil.

^{1}H NMR (400 MHz, $CDCl_3$, 295 K): δ 3.71 – 3.55 (m, 2H), 2.49 (dd, J = 9.3, 7.9, 6.3, 1.8 Hz, 4H), 1.72 – 1.48 (m, 6H), 1.47 – 1.28 (m, 4H), 1.27 – 1.14 (m, 1H), 0.97 – 0.80 (m, 16H), 0.04 (s, 6H).

^{13}C NMR (101 MHz, $CDCl_3$, 295 K): δ 61.30, 39.80, 36.34, 32.42, 31.81, 29.18, 27.17, 25.95, 22.02, 19.60, 18.30, 13.69, -5.30.

HRMS (ESI^+): *m/z* = 319.2480 $[M+H]^+$ (calculated 319.2491 for $C_{17}H_{39}OSSi^+$).

(S)-6-(butylthio)-3-methylhexan-1-ol (S8)

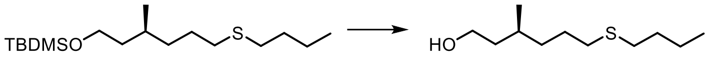

S7 **S8**

S7 (1.4 g, 4.4 mmol, 1 equiv.) was dissolved in 30 mL THF. TBAF (1 M solution in THF, 4.84 mL, 4.8 mmol, 1.1 equiv.) was added at 0 °C and the reaction mixture was stirred at room temperature for 12 h. The solvent was removed under reduced pressure and water (75 mL) was added. The reaction

mixture was extracted with ethylacetate (3*50 mL). The organic phase was washed with brine and dried over $MgSO_4$. The solvent was removed under reduced pressure and the crude material was purified by flash column chromatography (ethyl acetate/petroleum ether 20:80) to give **S8** (898 mg, 100%) as a colourless oil.

^{1}H NMR (400 MHz, $CDCl_3$, 295 K): δ 3.67 (qt, J = 10.6, 6.6 Hz, 2H), 2.49 (dtd, J = 7.9, 4.7, 2.4 Hz, 4H), 1.68 – 1.49 (m, 4H), 1.47 – 1.32 (m, 6H), 1.28 – 1.17 (m, 1H), 0.95 – 0.85 (m, 7H).

^{13}C NMR (101 MHz, $CDCl_3$, 295 K): δ 60.98, 39.77, 36.15, 32.33, 31.82, 31.76, 29.12, 26.99, 22.00, 19.50, 13.67.

HRMS (ESI$^-$): *m/z* = 205.1621 $[M+H]^+$ (calculated 205.1626 for $C_{11}H_{25}OS^+$).

(S)-butyl(6-iodo-4-methylhexyl)sulfide (S9)

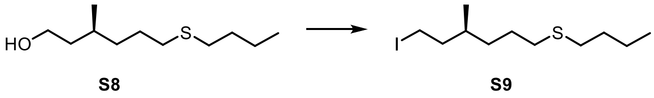

This compound was prepared following the same procedure as for **S6** to give **S9** (450 mg, 30%) as colourless oil.

^{1}H NMR (400 MHz, $CDCl_3$, 295 K): δ 3.25 (ddd, J = 9.6, 8.3, 5.7 Hz, 1H), 3.16 (ddd, J = 9.6, 8.0, 7.3 Hz, 1H), 2.55 – 2.44 (m, 4H), 1.86 (dddd, J = 13.5, 8.2, 7.3, 5.1 Hz, 1H), 1.71 – 1.50 (m, 6H), 1.47 – 1.34 (m, 3H), 1.23 (dddd, J = 13.2, 10.2, 7.7, 5.6 Hz, 1H), 0.97 – 0.85 (m, 6H).

^{13}C NMR (101 MHz, $CDCl_3$, 295 K): δ 40.64, 35.29, 33.48, 32.26, 31.82, 31.73, 26.86, 21.97, 18.60, 13.67, 5.08.

HRMS (ESI$^+$): *m/z* = 315.0626 $[M+H]^+$ (calculated 315.0638 for $C_{11}H_{24}IS^+$).

General procedure compounds S10 – S12

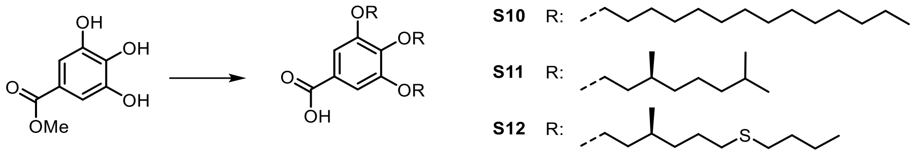

A 250 mL round-bottom flask was charged with 3,4,5-trihydroxymethylbenzoate (10 mmol, 1 equiv.), the corresponding alkyl halide (35 mmol, 3.5 equiv.), a spatula tip of potassium iodide and 100 mL of dry DMF. Anhydrous Cs_2CO_3 (42 mmol, 4.2 equiv.) was added and the reaction mixture was stirred at 130 °C until all starting material was consumed (NMR control). The solvent was removed under reduced pressure. The residue was suspended in 150 mL of water and extracted with CH_2Cl_2 (4*75 mL). The combined organic phase was dried over magnesium sulfate and evaporated under reduced pressure. To the residue were added 50 mL of MeOH, 150 mL of water and sodium hydroxide (0.15 mol, 15 equiv.). The reaction mixture was refluxed until all starting material was consumed (TLC control), cooled down to room temperature, carefully acidified with concentrated hydrochloric acid to pH 1 and extracted with CH_2Cl_2. The organic phase was dried over magnesium sulfate and the solvent was removed under reduced pressure. The crude material was purified by flash column chromatography (petroleum ether/ethyl acetate 9:1).

3,4,5-tris(tetradecyloxy)benzoic acid (S10)

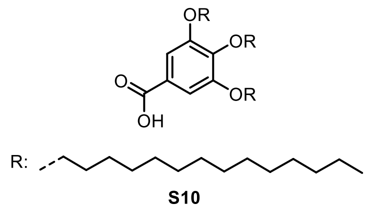

Yield: 87%, orange oil.

^{1}H NMR (400 MHz, $CDCl_3$, 295 K): δ 7.33 (s, 2H), 4.06 – 4.0 (m, 6H), 1.82 (p, J = 6.7 Hz, 4H), 1.74 (p, J = 6.9 Hz, 2H), 1.48 (p, J = 7.1 Hz, 6H), 1.27 (m, 66H), 0.88 (t, J = 6.7 Hz, 9H).

^{13}C NMR (101 MHz, $CDCl_3$, 295 K): δ 171.95, 152.82, 143.10, 123.61, 108.48, 73.53, 69.14, 31.93, 30.32, 29.75, 29.71, 29.67, 29.64, 29.56, 29.39, 29.38, 29.26, 26.07, 26.04, 22.69, 14.12.

HRMS (ESI$^+$): *m/z* = 759.6863 $[M + H]^+$ (calculated 759.6867 for $C_{49}H_{91}O_5^+$).

3,4,5-tris(((S)-3,7-dimethyloctyl)oxy)benzoic acid (S11)

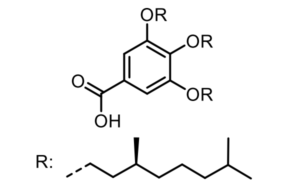

S11

Yield: 85%, orange oil.

^{1}H NMR (400 MHz, $CDCl_3$, 295 K): δ 7.34 (s, 2H), 4.15 – 4.20 (m, 6H), 1.92 – 1.79 (m, 3H), 1.76 – 1.67 (m, 3H), 1.67 – 1.57 (m, 2H), 1.57 – 1.47 (m, 4H), 1.39 – 1.22 (m, 9H), 1.22 – 1.08 (m, 9H), 0.95 (d, J = 6.6 Hz, 6H), 0.93 (d, J = 6.6 Hz, 3H), 0.87 (dd, J = 6.6, 2.3 Hz, 18H).

^{13}C NMR (101 MHz, $CDCl_3$, 295 K): δ 171.71, 152.88, 143.19, 123.62, 108.55, 77.20, 71.79, 67.52, 39.36, 39.27, 37.48, 37.33, 36.28, 29.86, 29.64, 27.98, 24.73, 22.69, 22.59, 19.59, 19.57.

HRMS (ESI^+): *m/z* = 591.4982 $[M + H]^+$ (calculated 591.4989 for $C_{37}H_{67}O_5^+$).

Compound S12

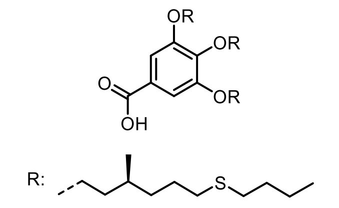

S12

Yield: 98%, orange oil.

^{1}H NMR (400 MHz, $CDCl_3$, 295 K): δ 7.32 (s, 2H), 4.12 – 4.00 (m, 6H), 2.58-2.43 (m, 12H), 1.95 – 1.80 (m, 3H), 1.80 – 1.69 (m,3H), 1.69 – 1.49 (m, 17H), 1.49 – 1.35 (m, 10H), 1.35 – 1.21 (m, 7H), 1.01 – 0.86 (m, 19H).

^{13}C NMR (101 MHz, $CDCl_3$, 295 K): δ 170.77, 152.79, 142.94, 123.60, 108.46, 77.20, 71.54, 67.34, 37.20, 36.50, 36.28, 36.15, 32.51, 32.43, 31.91, 31.89, 31.81, 29.67, 29.40, 27.17, 22.04, 19.51, 19.45, 13.71, 1.01.

HRMS (ESI^-): *m/z* = 727.4475 $[M - H]^-$ (calculated 727.4469 for $C_{40}H_{71}O_5S_3^-$).

General procedure compounds S13 – S15

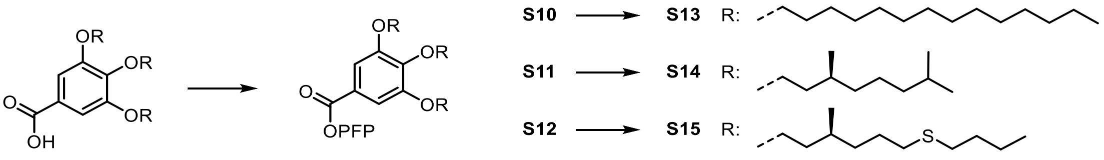

Acid **S10** – **S12** (11 mmol, 1 equiv.), 2,3,4,5,6-pentafluorophenol (13.2 mmol, 1.2 equiv.) and few crystals of DMAP were dissolved in 40 mL of CH_2Cl_2. EDC (13.2 mmol, 1.2 equiv.) was added. The

reaction mixture was stirred at room temperature overnight, diluted with 100 mL of CH_2Cl_2 and washed with water (4*75 mL). The organic phase was dried over magnesium sulfate and evaporated to give the title compound, which was used for the next step without further purification.

Pentafluorophenyl 3,4,5-tris(tetradecyloxy)benzoate (S13)

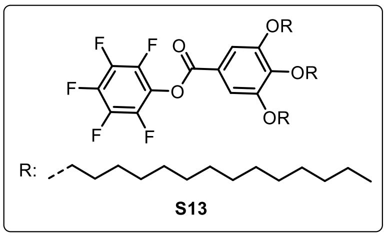

Crude yield: 96%, colourless solid.

¹H NMR (400 MHz, $CDCl_3$, 295 K): δ 7.39 (s, 2H), 4.09 – 4.0 (m, 6H), 1.83 (p, J = 6.8 Hz, 4H), 1.79 – 1.70 (p, J = 6.9 Hz, 2H), 1.48 (p, J = 7.4 Hz, 6H), 1.40 – 1.18 (m, J = 8.9 Hz, 74H), 0.88 (t, J = 6.7 Hz, 9H).

¹⁹F NMR (377 MHz, $CDCl_3$, 295 K): δ -152.23 – -153.02 (m, 2F), -158.39 (t, *J* = 22.3 Hz, 1F), -162.55 – -162.87 (m, 2F).

¹³C NMR (101 MHz, $CDCl_3$, 295 K): δ 171.25, 162.45, 153.12, 144.02, 143.09 – 142.16 (m), 141.15 – 139.78 (m), 139.63 – 138.57 (m), 137.33 – 136.32 (m), 121.01, 109.05, 73.68, 72.98, 69.32, 64.67, 31.93, 30.34, 29.70, 29.63, 29.56, 29.37, 29.25, 26.06, 22.69, 14.11.

Pentafluorophenyl 3,4,5-tris(((S)-3,7-dimethyloctyl)oxy)benzoate (S14)

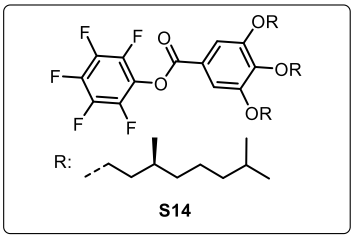

Crude yield: 90%, orange oil.

¹H NMR (400 MHz, $CDCl_3$, 295 K): δ 7.40 (s, 2H), 4.16 – 4.03 (m, 6H), 1.93 – 1.80 (m, 3H), 1.76 - 1.68 (m, 3H), 1.67 – 1.58 (m, 2H), 1.58 – 1.47 (m, 4H), 1.38 – 1.23 (m, 9H), 1.21 – 1.10 (m, 9H), 0.95 (d, J = 6.5 Hz, 6H), 0.92 (d, J = 6.6 Hz, 3H), 0.85 (dd, J = 6.6, 1.3 Hz, 18H).

¹⁹F NMR (377 MHz, $CDCl_3$, 295 K): δ -151.60 – -152.27 (m, 2F), -157.66 (t, *J* = 21.7 Hz, 2F), -162.01 (td, J = 22.5, 5.1 Hz, 1F).

¹³C NMR (101 MHz, $CDCl_3$): δ 162.47, 153.11, 143.93, 142.98 – 142.08 (m), 141.21 – 139.94 (m), 139.57 – 138.83 (m), 137.19 – 136.26 (m), 125.46, 121.03, 108.94, 77.20, 71.89, 67.58, 39.33, 39.23, 37.45, 37.30, 36.21, 29.79, 29.58, 27.98, 24.73, 22.69, 22.58, 19.55.

Compound S15

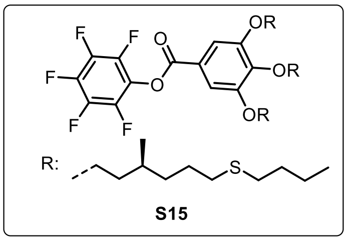

Crude yield: 88%, orange oil.

^{1}H NMR (400 MHz, $CDCl_3$, 295 K): δ 7.39 (s, 2H), 4.15 – 4.01 (m, 6H), 2.57 – 2.43 (m, 14H), 1.95 – 1.80 (m, 3H), 1.80 – 1.70 (m, 4H), 1.69 – 1.50 (m, 17H), 1.50 – 1.35 (m, 11H), 1.34 – 1.22 (m, 4H), 1.02 – 0.86 (m, 21H).

^{19}F NMR (377 MHz, $CDCl_3$, 295 K): δ -151.76 – -152.42 (m, 2F), -157.65 (t, *J* = 21.7 Hz, 1F), -162.00 (td, *J* = 22.6, 5.1 Hz, 2F).

^{13}C NMR (101 MHz, $CDCl_3$, 295 K): δ 162.41, 153.05, 143.83, 142.85 – 142.43 (m), 140.36 – 139.82 (m), 139.54 – 183.91 (m), 137.08 – 136.24 (m), 121.14, 108.99, 106.56, 71.70, 67.48, 37.18, 36.47, 36.29, 36.24, 36.19, 36.11, 32.50, 32.42, 31.91, 31.81, 31.79, 29.64, 29.38, 27.16, 27.13, 22.03, 22.02, 19.50, 19.43, 13.70, 13.68.

General procedure compounds M_1 – M_5

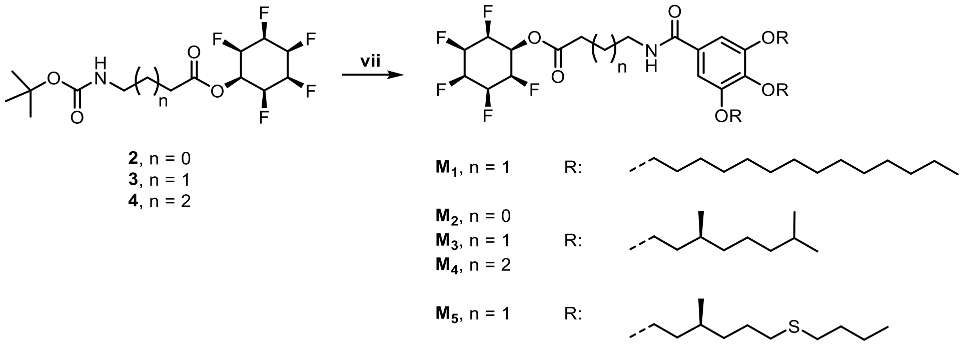

To a suspension of compound **2 - 4** (2 mmol, 1 equiv.) in 2 mL of anhydrous CH_2Cl_2 was added 2 mL of TFA at 0 °C. The reaction mixture was stirred at room temperature until all starting material was consumed. CH_2Cl_2 and TFA were removed under vacuum. Pentafluorophenyl ester **S13** – **S15** (4 mmol, 2 equiv.), 5 mL of DMF and triethylamine 3 mL were added subsequently and the reaction mixture was stirred at room temperature overnight. After removal of solvents under reduced pressure the crude material was purified by flash column chromatography (CH_2Cl_2 to CH_2Cl_2/acetone 8:2) to yield the title compound.

Comments on reaction conditions, side products and purification:

1. DMF is needed to completely dissolve starting materials containing all-*cis* $C_6H_6F_5$ group and to prevent gelation which sometimes can happen if DCM alone is used as a solvent.
2. To completely remove pentafluorophenol from the reaction mixture, sometimes two purifications by flash column chromatography are needed.
3. For the linker based on γ-aminobutyric acid approximately 13% of butyrolactam was formed as a side product.

Compound M_1

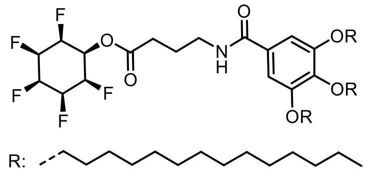

M_1

Yield: 71%, colourless solid.

^{1}H NMR (400 MHz, $CDCl_3$, 295 K): δ 6.95 (s, 2H), 6.38 (br s, 1H), 5.52 – 4.22 (m, 5H), 4.03 - 3.92 (m, 6H), 3.50 (q, J = 6.5 Hz, 2H), 2.58 (t, J = 7.0 Hz, 2H), 2.00 (p, J = 6.8 Hz, 2H), 1.83 – 1.65 (m, 7H), 1.51 – 1.39 (m, 7H), 1.38 – 1.16 (m, 66H), 0.88 (t, J = 6.7 Hz, 9H).

^{19}F NMR (377 MHz, $CDCl_3$, 295 K) mixture of conformers: δ -209.83 (br s, 2F), -210.66, -211.73, -215.95 (d, *J* = 26.2 Hz, 2F), -215.78, -216.83 (br s, 1F).

^{13}C NMR (101 MHz, $CDCl_3$, 295 K): δ 172.24, 167.52, 153.10, 141.06, 129.14, 105.43, 87.79 – 86.82 (m), 85.92 – 84.55 (m), 83.76 – 82.73 (m), 73.50, 69.28, 66.47 – 65.85, 38.89, 31.92, 31.17, 30.31, 29.73, 29.65, 29.58, 29.42, 29.36, 26.08, 24.73, 22.68, 14.10.

HRMS (ESI^+): *m/z* = 1016.7679 $[M+H]^+$ (calculated 1016.7706 for $C_{59}H_{103}F_5NO_6^+$).

Compound M_2

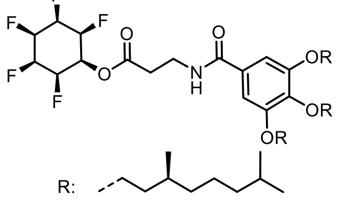

M_2

Yield: 64%, glassy colourless solid.

^{1}H NMR (400 MHz, $CDCl_3$, 295 K): δ 6.96 (s, 2H), 6.77 (br s, 1H), 5.45 – 4.38 (m, 5H), 4.08 – 3.93 (m, 6H), 3.73 (q, J = 6.1 Hz, 2H), 2.79 (t, J = 5.8 Hz, 2H), 1.89 – 1.75 (m, 3H), 1.74 – 1.63 (m, 3H), 1.63 – 1.45 (m, 6H), 1.38 – 1.20 (m, 10H), 1.20 – 1.04 (m, 9H), 0.94 – 0.89 (m, 9H), 0.86 (d, J = 6.5 Hz, 18H).

^{19}F NMR (377 MHz, $CDCl_3$, 295 K) mixture of conformers: δ -207.32 (br s, 2F), -208.13 (br s, 1F), -209.13 (br s, 0.5F), -212.01 – -212.60 (m, 2F), -213.01 (br s, 1F), -214.25 (s. 0.5F).

^{13}C NMR (126 MHz, $CDCl_3$, 295 K): δ 170.74, 167.48, 153.06, 140.94, 128.96, 105.34, 88.17 – 86.63 (m), 86.63 – 84.12 (m), 84.05 – 81.71 (m), 71.73, 67.43, 66.60 – 66.03 (m), 39.32, 39.24, 37.48, 37.35, 37.31, 36.35, 35.46, 34.57, 29.77, 29.65, 27.94, 24.70, 24.67, 22.66, 22.56, 19.51, 19.42.

HRMS (ESI^+): *m/z* = 872.5128 $[M+K]^+$ (calculated 872.5230 for $C_{46}H_{75}F_5NKO_6^+$).

Compound M_3

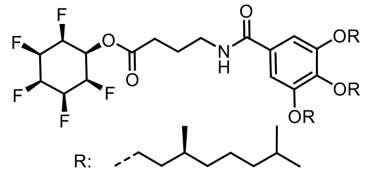

M_3

Yield: 87%, glassy colourless solid.

^{1}H NMR (500 MHz, $CDCl_3$, 295 K): δ 6.97 (s, 2H), 6.41 (t, J = 5.9 Hz, 1H), 5.47 – 4.37 (m, 6H), 4.09 – 3.92 (m, 6H), 3.49 (q, J = 6.6 Hz, 2H), 2.57 (t, J = 7.0 Hz, 2H), 1.99 (p, J = 6.9 Hz, 2H), 1.90 – 1.76 (m, 3H), 1.75 - 1.64 (m, 4H), 1.63 – 1.56 (m, 7H), 1.38 – 1.21 (m, 10H), 1.21 – 1.08 (m, 10H) 0.95 – 0.89 (m, 9H), 0.86 (d, J = 6.6 Hz, 20H).

^{19}F NMR (377 MHz, $CDCl_3$, 295 K) mixture of conformers: δ -208.81 (br s, 2F), -209.62, -210.64, -214.94, -215.97 (br s, 1F).

^{13}C NMR (126 MHz, $CDCl_3$, 295 K): δ 172.21, 167.56, 153.12, 141.08, 129.17, 105.45, 87.84 – 86.73, 86.20 – 84.41, 84.11 – 82.60, 83.38, 77.20, 71.74, 67.58, 66.49 – 65.97, 39.33, 39.24, 38.93, 37.48, 37.35, 37.31, 36.37, 31.18, 29.79, 29.66, 27.95, 24.71, 22.67, 22.57, 19.52, 19.47.

HRMS (ESI^+): *m/z* = 848.5829 $[M+H]^+$ (calculated 848.5822 for $C_{47}H_{79}F_5NO_6^+$).

Compound M_4

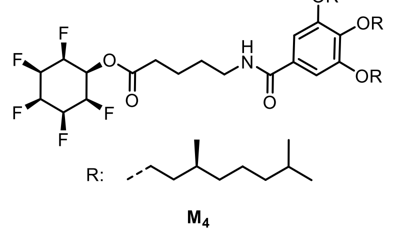

M_4

Yield: 26%, glassy colourless solid.

^{1}H NMR (400 MHz, acetone-*d_6*, 295 K): δ 7.76 (s, 1H), 7.22 (s, 2H), 5.59 – 4.86 (m, 8H), 4.14 – 3.93 (m, 6H), 3.44 – 3.31 (m, 3H), 2.58 – 2.44 (m, 3H), 2.56 – 2.45 (m, 3H), 1.92 – 1.46 (m, 20H), 1.44 – 1.25 (m, 11H), 1.25 – 1.09 (m, 10H), 0.98 – 0.94 (m, 9H), 0.90 – 0.85 (m, 19H).

^{19}F NMR (377 MHz, acetone-d_6, 295 K): δ -211.44 (br s, 2F), -215.89 (br s, 2F), -217.85 (br s, 1F).

^{13}C NMR (101 MHz, acetone-d_6, 295 K): δ 173.31, 167.43, 154.29, 141.93, 131.46, 107.09, 101.42, 90.01 – 88.59, 88.11 – 86.68, 86.12 – 84.65, 72.34, 68.10 – 67.44, 40.66, 40.56, 40.45, 40.33, 38.76, 38.63, 37.80, 34.51, 34.29, 31.03, 29.25, 26.06, 23.56, 23.46, 23.15, 20.60, 20.40.

HRMS (ESI$^+$): *m/z* = 884.5575 $[M+Na]^+$ (calculated 884.5803 for $C_{48}H_{80}F_5NNaO_6^+$).

Compound M$_5$

Yield: 23%, glassy colourless solid.

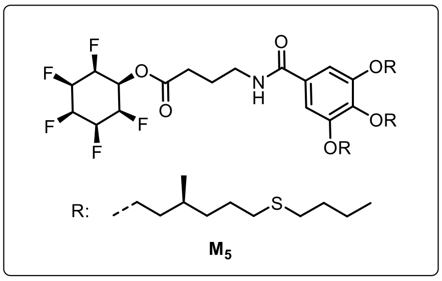

^{1}H NMR (500 MHz, $CDCl_3$, 295 K): δ 6.96 (s, 2H), 6.25 (br s, 1H), 5.48 – 4.35 (m, 6H), 4.10 – 3.95 (m, 6H), 3.53 (q, J = 6.5 Hz, 2H), 2.60 (t, J = 7.2 Hz, 2H), 2.54 - 2.44 (m, 12H), 2.02 (p, J = 6.8 Hz, 2H), 1.91 – 1.79 (m, 4H), 1.77 – 1.68 (m, 4H), 1.68 – 1.52 (m, 16H), 1.51 – 1.35 (m, 11H), 1.35 – 1.21 (m, 6H), 0.98 – 0.94 (m, 8H), 0.z94 – 0.89 (m, 11H).

^{19}F NMR (377 MHz, $CDCl_3$, 295 K) mixture of conformers: δ -209.48 (br s, 2F), -210.32, -211.32, -214.77 (d, J = 24 Hz, 2F), -215.53, -216.53 (br s, 1F).

^{13}C NMR (126 MHz, $CDCl_3$, 295 K): δ 172.26, 167.44, 153.06, 141.05, 129.27, 105.54, 87.55 – 86.43 (m), 86.15 – 84.15 (m), 84.11 – 82.73 (m), 71.49, 67.49, 66.49 – 65.77, 38.87, 37.20, 36.52, 36.26, 36.12, 32.53, 32.45, 31.91, 31.82, 31.22, 29.64, 29.43, 27.17, 24.74, 22.03, 19.49, 19.45, 13.69.

HRMS (ESI$^-$): *m/z* = 1020.5067 $[M+Cl]^-$ (calculated 1020.5075 for $C_{50}H_{84}F_5NS_3O_6Cl^-$).

Synthesis of *Me*M3

Methyl 4-((tert-butoxycarbonyl)(methylamino)butanoate (S17)

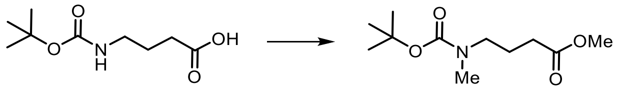

S17

A 100 mL heat gun-dried Schlenk flask was charged with γ-(Boc-amino)butyric acid (2 g, 9.9 mmol, 1 equiv.), methyl iodide (6 mL, 99 mmol, 10 equiv.) and 40 mL of dry DMF under argon. NaH (2.36 g of 60% (w%) solution in mineral oil, 60 mmol, 6 equiv.) was added at 0 °C, the reaction mixture was stirred at this temperature for 2 h and ice bath was removed and reaction was stirred at room temperature overnight. Excess of NaH was carefully quenched with water, 50 mL of concentrated aqueous solution of ammoniac was added. The reaction mixture was diluted with 150 mL of water and extracted with ethyl acetate (4*50 mL). The combined organic phase was washed with water (3*50 mL) and brine. The organic phase was dried over sodium sulfate and evaporated under reduced pressure to give **S17** (2.2 g, 97%) as yellowish oil ,which was used for the next step without purification.

^{1}H NMR (400 MHz, $CDCl_3$, 295 K): δ 3.64 (s, 3H), 3.22 (t, J = 7.1 Hz, 2H), 2.81 (s, 3H), 2.29 (t, J = 7.4 Hz, 2H), 1.81 (p, J = 7.3 Hz, 2H), 1.42 (s, 9H).

^{13}C NMR (101 MHz, $CDCl_3$, 295 K): δ 173.52, 155.69, 79.29, 51.57, 51.51, 48.02, 47.57, 37.02, 33.95, 32.68, 31.86, 30.99, 29.96, 29.63, 29.29, 28.35, 27.02, 23.08, 22.98, 22.62, 19.66, 17.15, 14.05.

HRMS (ESI$^+$): *m/z* = 254.1364 $[M+Na]^+$ (calculated 254.1368 for $C_{11}H_{21}NO_4Na^+$).

Compound S18

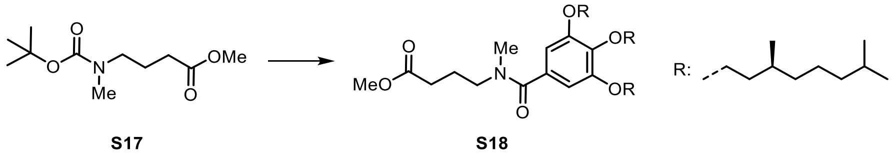

S17 S18

S17 (254 mg, 1.1 mmol, 1 equiv.) was dissolved in CH_2Cl_2 (1 mL) and TFA (1 mL) was added. The reaction mixture was stirred for 30 minutes and the solvent was removed under vacuum. To the obtained solid material **S14** (1g, 1.32 mmol, 1.2 equiv), few crystals of DMAP, CH_2Cl_2 (2 mL) and TEA (1 mL) were added subsequently. The reaction mixture was stirred for 12h at room temperature.

The solvent was removed under reduced pressure and the crude material was purified by flash column chromatography (petroleum ether/ethyl acetate 8:2) to give **S18** (515 mg, 67%) as a colourless oil.

^{1}H NMR (400 MHz, $CDCl_3$, 295 K): δ 6.56 (s, 2H), 4.05 – 3.09 (m, 6H), 3.75 – 3.20 (m, 5H), 3.10 – 2.92 (br, 3H), 2.62 – 2.08 (br, 2H), 2.08 – 1.94 (br, 1H), 1.94 – 1.75 (m, 4H), 1.74 – 1.45 (m, 10H), 1.39 – 1.20 (m, 10H), 1.20 – 1.08 (m, 10H), 0.92 (d, J = 6.6 Hz, 6H), 0.91 (d, J = 6.6 Hz, 3H), 0.86 (d, J = 6.7 Hz, 18H).

^{13}C NMR (101 MHz, $CDCl_3$, 295 K): δ 171.72, 153.08, 139.11, 130.94, 105.46, 105.06, 77.21, 71.66, 67.48, 51.67, 39.33, 39.23, 37.48, 37.30, 36.32, 29.79, 29.64, 27.95, 24.70, 22.68, 22.57, 19.51.

HRMS (ESI^+): *m/z* = 704.5808 $[M+H]^+$ (calculated 704.5829 for $C_{43}H_{78}NO_6^+$).

Compound *Me*M$_3$

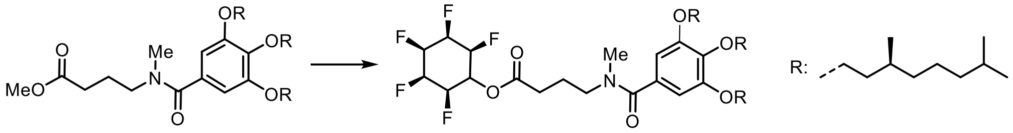

S18 *Me*M$_3$

S18 (1.1 g, 1.38 mmol, 1 equiv.) and sodium hydroxide (1 g, 25 mmol, 18 equiv.) were refluxed in 25 ml of water/methanol (3:1) mixture until all starting material was consumed. The reaction mixture was acidified at 0 °C to pH=1 with a concentrated aqueous solution of hydrochloric acid and extracted with CH_2Cl_2 (3*50 mL). The organic phase was dried over anhydrous sodium sulfate and evaporated under reduced pressure. The obtained orange oil was dissolved in 50 mL of CH_2Cl_2. Pentafluorophenol (305 mg, 1.66 mmol, 1.2 equiv.), few crystals of DMAP and EDC (312 mg, 1.66 mmol, 1.2 equiv.) were added subsequently and the reaction mixture was stirred overnight at room temperature. 100 mL of water were added and extracted with CH_2Cl_2 (4*50 mL). Organic phase was dried over anhydrous sodium sulfate and solvent was removed under reduced pressure. To the residue **1** (201 mg, 1.06 mmol, 0.77 equiv.), 1 mL of dry DMF and 1 mL of TEA were added. The reaction mixture was stirred at 120 °C for 4 h after which solvents were removed and crude material was purified via flash column chromatography (gradient elution CH_2Cl_2 to CH_2Cl_2/acetone 8:2) to give ***Me*M$_3$** (870 mg, 65%) as a colourless solid.

^{1}H NMR (500 MHz, $CDCl_3$, 295 K): δ 6.58 (s, 2H), 5.44 – 4.30 (m, 5H), 4.10 – 3.87 (m, 6H), 3.69 – 3.28 (m, 2H), 3.14 – 2.92 (m, 3H), 2.68 – 2.26 (m, 2H), 2.10 – 1.77 (m, 5H), 1.77 – 1.66 (m, 3H),

1.66 – 1.49 (m, 6H), 1.42 – 1.24 (m, 9H), 1.25 – 1.11 (m, 9H), 0.93 (d, J = 6.6 Hz, 6H), 0.92 (d, J = 6.6 Hz, 3H), 0.87 (d, J = 6.6 Hz, 17H).

^{19}F NMR (377 MHz, $CDCl_3$, 295 K): δ -210.26 (d, J = 93.3 Hz, 2F), -211.18, -212.36, -215.75 (br s, 2F), -216.43, -217.12 (br s, 1F).

^{13}C NMR (126 MHz, $CDCl_3$, 295 K): δ 171.67, 153.18, 139.34, 131.56, 131.10, 105.55, 104.75, 87.63 – 86.68 (m), 86.02 – 84.59 (m), 83.91 – 83.05 (m), 71.75, 66.97 – 65.95 (m), 50.20, 46.10, 39.35, 39.30, 39.28, 39.25, 39.20, 37.51, 37.42, 37.34, 37.25, 36.34, 32.80, 30.78, 29.96, 29.82, 29.72, 27.96, 24.71, 22.68, 22.60, 22.57, 19.54, 19.49.

HRMS (ESI^+): *m/z* = 862.5955 $[M+H]^+$ (calculated 862.5984 for $C_{48}H_{81}F_5NO_6^+$).

Synthesis of Ref2

Cyclohexyl 4-((tert-butoxycarbonyl)amino)butanoate (S19)

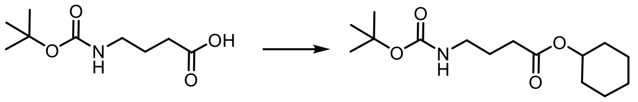

S19

γ-(Boc-amino)butyric acid (470 mg, 2.3 mmol, 1 equiv.), cyclohexanol (481 µL, 4.6 mmol, 2 equiv.) and few crystals of DMAP were dissolved in CH_2Cl_2. EDC (441 mg, 2.3 mmol, 1 equiv.) was added and the reaction mixture was stirred overnight at room temperature. The reaction mixture was diluted with ethyl acetate (50 mL), washed with water (3*25mL) and brine (2*25 mL). The organic phase was dried over sodium sulfate and evaporated under reduced pressure. Excess of cyclohexanol was distilled off under vacuum to give **S19** (450 mg, 69%) as a colourless oil.

^{1}H NMR (400 MHz, $CDCl_3$, 295 K): δ 4.74 (td, J = 8.9, 4.1 Hz, 1H), 4.63 (br, 1H), 3.16 (q, J = 6.6 Hz, 2H), 2.32 (t, J = 7.3 Hz, 2H), 1.87 – 1.75 (m, 4H), 1.75 – 1.64 (m, 2H), 1.59 – 1.49 (m, 1H), 1.43 (s, 9H), 1.40 – 1.16 (m, 5H).

^{13}C NMR (101 MHz, $CDCl_3$, 295 K): δ 172.72, 155.90, 79.17, 77.21, 72.72, 39.98, 32.04, 31.61, 28.39, 25.34, 23.74.

HRMS (ESI^+): *m/z* = 593.3778 $[2M+Na]^+$ (calculated 593.3772 for $C_{30}H_{54}N_2O_8Na^+$).

Compound Ref_2

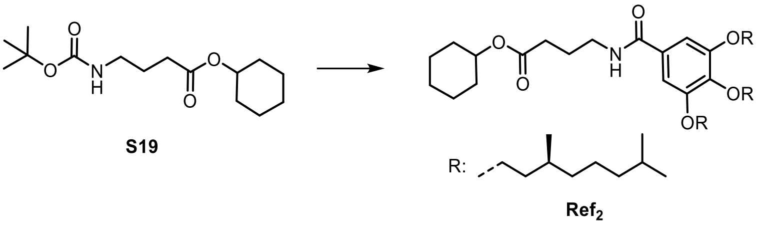

S19 (450 mg, 1.58 mmol, 1 equiv.) was stirred in 1 mL of CH_2Cl_2/TFA (1:1 v/v) mixture for 30 min. Solvent was removed under reduced pressure. **S14** (1.5 g, 1.9 mmol, 1.2 equiv.), few crystals of DMAP, 1 mL of CH_2Cl_2 and 1 mL of TEA were added subsequently and the reaction mixture was stirred overnight at room temperature. Solvent was removed under reduced pressure and crude material was purified by flash column chromatography (petroleum ether/ethyl acetate 8:2) to yield **Ref_2** (684 mg, 57%) as yellow oil, which solidified upon standing.

^{1}H NMR (400 MHz, $CDCl_3$, 295 K): δ 7.00 (s, 2H), 6.69 (t, J = 5.4 Hz, 1H), 4.74 (td, J = 8.9, 4.1 Hz, 1H), 4.21 – 3.86 (m, 6H), 3.50 (q, J = 6.3 Hz, 2H), 2.45 (t, J = 6.8 Hz, 2H), 1.96 (p, J = 6.7 Hz, 2H), 1.91 – 1.76 (m, 5H), 1.75 – 1.65 (m, 5H), 1.64 – 1.57 (m, 2H), 1.57 – 1.46 (m, 5H), 1.45 – 1.21 (m, 16H), 1.21 – 1.09 (m, 9H), 0.94 (d, J = 6.6 Hz, 6H), 0.91 (d, J = 6.7 Hz, 3H), 0.86 (d, J = 6.6 Hz, 9H).

^{13}C NMR (101 MHz, $CDCl_3$, 295 K): δ 173.68, 167.47, 153.06, 140.97, 129.17, 105.44, 76.12, 73.15, 71.70, 67.53, 40.11, 39.35, 39.26, 37.49, 37.35, 37.30, 36.38, 32.65, 31.58, 29.83, 29.64, 27.98, 25.29, 24.73, 24.19, 23.73, 22.70, 22.59, 19.54.

HRMS (ESI$^+$): *m/z* = 758.6307 $[M+H]^+$ (calculated 758.6299 for $C_{47}H_{84}NO_6^+$).

N-butyl-3,4,5-tris(((S)-3,7-dimethyloctyl)oxy)benzamide (**Ref_3**)

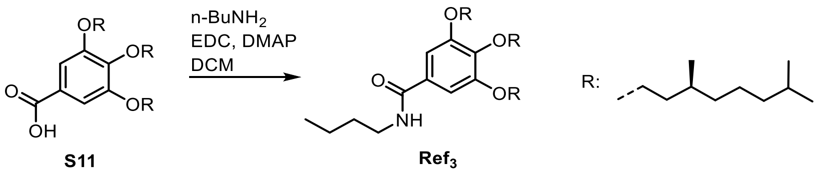

S11 (180 mg, 0.31 mmol, 1 equiv.), butan-1-amine (61 µL, 0.62 mmol, 2 equiv.) and few crystals of DMAP were dissolved in 10 mL of CH_2Cl_2. EDC (116 mg, 0.62 mmol, 2 equiv.) was added. The reaction mixture was stirred at room temperature overnight, diluted with 50 mL of CH_2Cl_2 and washed with water (4*50 mL). The organic phase was dried over magnesium sulfate and evaporated.

Crude material was purified by flash column chromatography to give **Ref$_3$** (160 mg, 81%) as a colouress oil.

^{1}H NMR (400 MHz, $CDCl_3$, 295 K): δ 6.95 (s, 2H), 6.01 (t, J = 5.8 Hz, 1H), 4.09 – 3.94 (m, 6H), 3.43 (q, J = 7.2 Hz, 2H), 1.91 – 1.76 (m, 3H), 1.76 – 1.65 (m, 2H), 1.65 – 1.55 (m, 4H), 1.55 – 1.46 (m, 3H), 1.46 – 1.36 (m, 2H), 1.36 – 1.20 (m, 7H), 1.20 – 1.07 (m, 9H), 0.99 – 0.89 (m, 12H), 0.86 (d, J = 6.6 Hz, 18H).

^{13}C NMR (101 MHz, $CDCl_3$, 295 K): δ 167.42, 153.09, 141.05, 129.90, 105.63, 71.70, 67.69, 39.88, 39.35, 39.26, 37.49, 37.33, 37.30, 36.38, 31.82, 29.82, 29.64, 27.97, 24.72, 24.70, 22.69, 22.60, 22.58, 20.17, 19.57, 19.54, 13.79.

HRMS (ESI$^+$): *m/z* = 668.5596 $[M+Na]^+$ (calculated 668.5588 for $C_{41}H_{75}NNaO_4^+$)

Computational Methods

Parametrization

The molecular models of the molecules **M$_2$**, **M$_3$**, **M$_4$** and ***Me*M$_3$** herein studied, along with the solvent composed by cyclohexane and chloroform were parametrized by using the General Amber Force Field (GAFF),[5] and the partial charges were modelled by using the AM1-BCC method.[6] These parameters were extracted by using the software Antechamber.[7]

Molecular dynamics setup

All the simulation herein performed, were run on GROMACS-2018.6[8] patched with plumed-2.5.[9] All systems were simulated for 1 μs of MD at the temperature of 293 K and pressure of 1 atm in explicit TIP3P water molecules[10] in periodic boundary NPT conditions (constant N: number of particles, P: pressure and T: temperature), employing the v-rescale thermostat[11] and the Berendsen barostat (semi-isotropic barostat during the study of fiber stability).[12] A timestep of 2 fs was used in the MD simulations. The electrostatic interactions were treated using particle mesh Ewald (PME).[13] The cutoff lengths of the real summation and of the VdW were set to 1.0 nm. The dynamics of the hydrogens was constrained using the LINCS algorithm.[14] All the systems studied were simulated with a solvent composition 84:16 cyclohexane:chloroform.

While for the studies of conformations and self-assembly the systems after being minimized were equilibrated with short runs followed by the 1μs MD simulation, the MD simulations of the preformed fibers were performed by first pre-equilibrating the “tails” of the monomers, by keeping restrained the atoms of the “core” (Supplementary Fig. 25b) for 10ns. After this it followed 1μs of MD as production run.

Metadynamics

In this work, we employed metadynamics (MetaD) simulations[15] to enhance the exploration of the conformational space of the single monomers. We used two collective variables (CV): CV1, monitoring the orientation of the pentafluorocyclohexane moiety (F_5-CHX) with respect to the amide moiety, and CV2, controlling the opening of the "core" of the monomer. CV2 is defined as the radius of gyration of the heavy atoms of the "core", while CV1 is defined as the difference between two distances (d_{FsH} and d_{HsO}, in Supplementary Fig. 25c). d_{FsH} is the distance between geometrical center of the fluorides and the amide nitrogen, while d_{HsO} is the distance between the geometrical center of the F_5-CHX-hydrogens and the amide-oxygen. Small values of CV2 indicate that the monomer core is closed, while large values indicate that the core is open. Positive values of CV1 indicate that the system tends to orient F_5-CHX-hydrogens toward the oxygen, while negative values show that the fluorides are closer to the amide group. These metadynamics calculations were performed in its well-tempered variant (WT-MetaD), which allows to obtain a smoother convergence of the system.[16] During the WT-MetaD run, the bias was deposited along CV1 and CV2 in the form of Gaussian-shaped kernels of height *0.8* kJ/mol and σ equal to 0.02 and 0.01 nm along CV1 and CV2 respectively while the deposition rate was *1* ps^{-1}. The bias factor of this WT-MetaD run was set to 15.

Intrinsic structural dynamics of the fiber

In order to deeper investigate on the dynamic of the **M_3** and **M_5** monomers within the fibers, we performed an unsupervised machine-learning analysis that allowed us to classify the arrangement, order and dynamics of the **M_3** and **M_5** monomers within the fiber in a robust, abstract and high-dimensional way. The configuration of each monomer from MD trajectory was classified according to its molecular environment by means of a Smooth Overlap of Atomic Positions (SOAP),[17] an approach that encodes the atomic/molecular environment of each molecule as a rotationally invariant representation (SOAP vectors). The SOAP vectors of each molecule were computed, considering five centers, defined as the center of the cyclohexane, the center of the amide for M_3 and M_5, the alkyl centers in the three tails on **M_3** (Supplementary Fig. 52a), and the sulfurs atoms on **M_5** (Supplementary Fig. 52b) that occupy the equivalent position of the alkyl moieties in **M_3**. Based on this description, the behavior of each monomer in the system is represented (and simplified) by the movements and behaviors of these five centers, which are monitored and characterized by the SOAP vectors. This approach was previously demonstrated to be capable of providing remarkably rich insights into the internal structure and dynamics of supramolecular polymers.[18] We carried out this SOAP analysis with the Python package *DScribe*,[19] setting the input parameters as r_{cut} = 60Å, n_{max} = 5, l_{max} = 5, and leaving the other parameters as default.

The SOAP centers could then be classified into different states by applying a clustering algorithm to the SOAP dataset, to obtain the most probable states in the multidimensional descriptor space. First, we reduced the high-dimensional SOAP features via principal component analysis (PCA) by retaining only the first three components. This approach reduced the computational cost for the processing of the data, while maintaining a high level of accuracy (up to ~86% of the information preserved). Linear PCA dimensionality reduction was performed using the Python package *Scikit-Learn.*[20] At this point, we performed unsupervised clustering of the dimensionally reduced dataset by applying the density-based clustering scheme Probabilistic Analysis of Molecular Motifs (PAMM),[18, 21, 22] which allowed us to classify all possible arrangements of the SOAP centers in the monomers within the fiber during the MD trajectories. The different clusters detected by the PAMM method are represented by different colors and are projected onto the first two PCA components. The obtained microclusters were then merged into dominant macroclusters (see references 18, 21 and 22 for details), in order to obtain a relevant classification with a more direct interpretation in terms of the structural features within the aggregate (Figs. 7c,e). In fig. 7c we can observe the labelling of each center included in the SOAP analysis. From this we can understand that the red-labelled SOAP centers are related to the cyclohexane centers while the blue-labelled SOAP centers are related to the center of the amide – these identify the backbone of the fibers. The other three macroclusters (gray, cyan and green colored) are related to the SOAP centers located on the tails,and are indicative of the fibers' surface. In particular, the green centers are localized in closer proximity of the fiber center, while the cyan ones represent an intermediate layer, and the gray ones identify the most external ones.

Finally, as described in detail in a previous work,[18] the time evolution of this clustering of SOAP centers states, and their dynamic interconversion along the MD trajectory allowed us to retrieve the probability of transition among different states, thus providing a clear picture of the intrinsic dynamics of the fiber. The transition probabilities reported in Fig. 7f of the main text were obtained with this methodology. The rates for the transitions between them can be then simply estimated by dividing these transition probabilities by the timelapse between the analysed snapshots (the examined snapshots are distant in time 0.1 ns from each other in our case.

Supplementary Figures

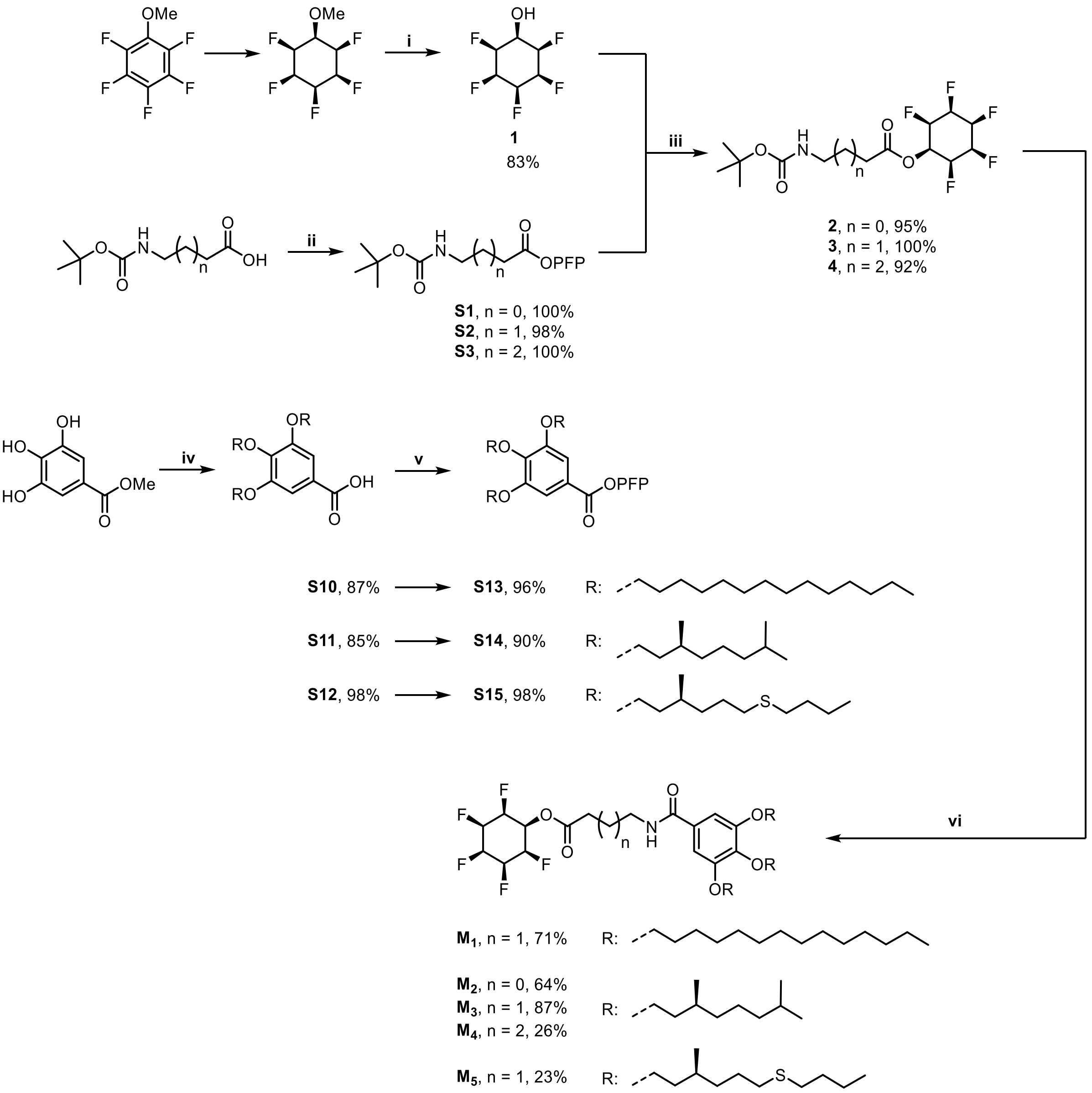

Supplementary Figure 1 | Overview on the synthesis of monomers **M$_1$ – M$_5$**. Reaction conditions: (i) n-BuSH, $AlCl_3$, CH_2Cl_2, room temperature, 14h; (ii) 2,3,4,5,6-pentafluorophenol (PFPOH), EDC, DMAP, CH_2Cl_2, room temperature, 12h; (iii) DMF, TEA, 120 °C, 2h; (iv) 1) R-X, Cs_2CO_3, KI, DMF, 120 °C, 3h; 2) NaOH, MeOH, H_2O; (v) PFPOH, EDC, DMAP, CH_2Cl_2, room temperature, 12h; (vi) 1) TFA, CH_2Cl_2, 0 °C, 2h; 2) CH_2Cl_2, DMAP, TEA, room temperature, 12h.

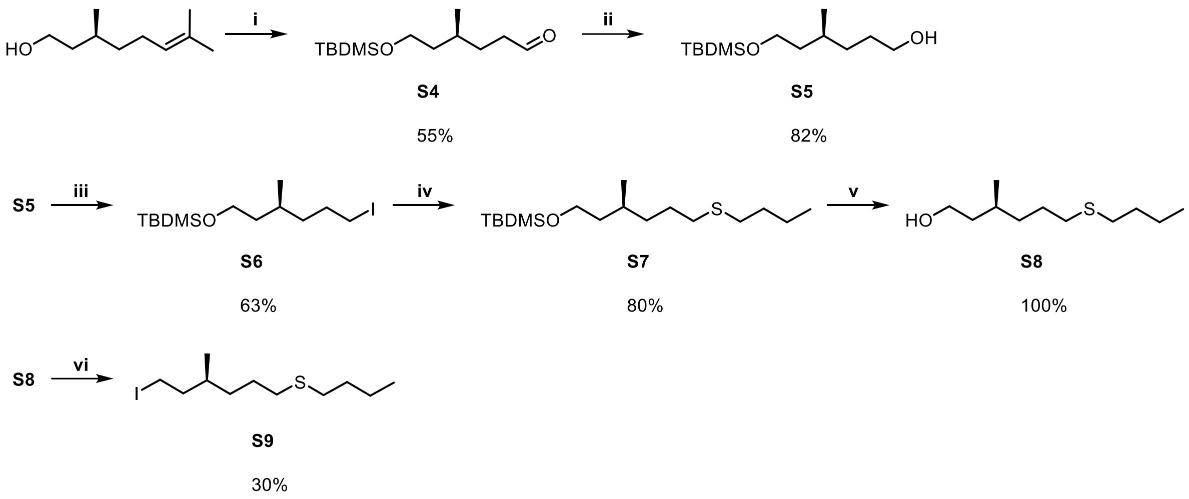

Supplementary Figure 2 | Overview on synthesis of **S9**. Reaction conditions: (i) 1) OsO_4, $NaIO_4$, 1,4-dioxane, H_2O, room temperature, 24h; 2) TBDMSCl, imidazole, CH_2Cl_2, room temperature, 12h; (ii) $NaBH_4$, MeOH, 0 °C to room temperature, 12h; (iii) I_2, PPh_3, imidazole, acetonitrile, 0 °C to room temperature, 2h; (iv) n-BuSH, Cs_2CO_3, TBAI, DMF, 0 °C to room temperature, 2h; (v) TBAF, THF, 0 °C to room temperature, 12h; (vi)) I_2, PPh_3, imidazole, acetonitrile, 0 °C to room temperature, 2h.

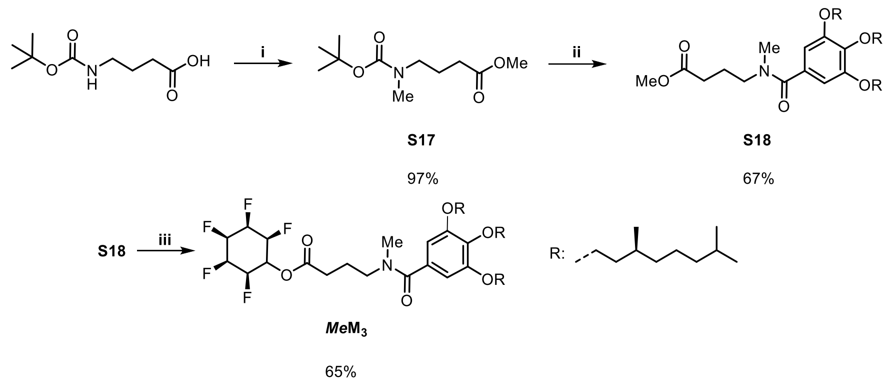

Supplementary Figure 3 | Overview on synthesis of ***Me*M3.** Reaction conditions: (i) MeI, NaH, DMF, 0 °C to room temperature, 12 h; (ii) 1) TFA, CH_2Cl_2, room temperature, 30 min; 2) **S14**, CH_2Cl_2, TEA, room temperature, 12 h; (iii) 1) NaOH, THF, H_2O, reflux, 8 h; 2) PFPOH, EDC, DMAP, CH_2Cl_2, room temperature, 12 h; 3h) **1**, DMF, TEA, 120 °C, 4 h.

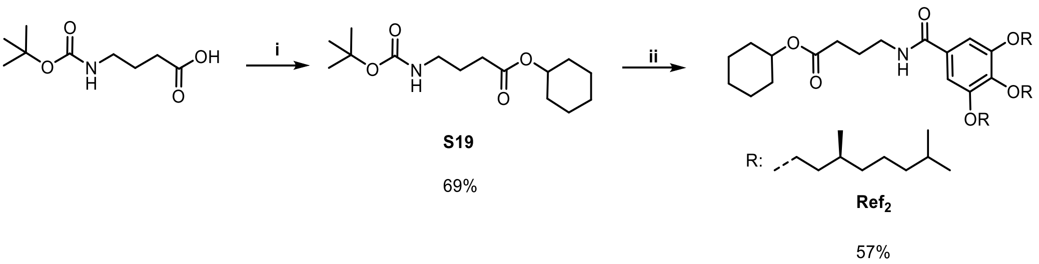

Supplementary Figure 4 | Overview on synthesis of **Ref2**. Reaction conditions: (i) Cyclohexanol, EDC, DMAP, CH_2Cl_2, room temperature, 12 h; (ii) 1) TFA, CH_2Cl_2, 0 °C, 2h; 2) CH_2Cl_2, DMAP, TEA, room temperature, 12h.

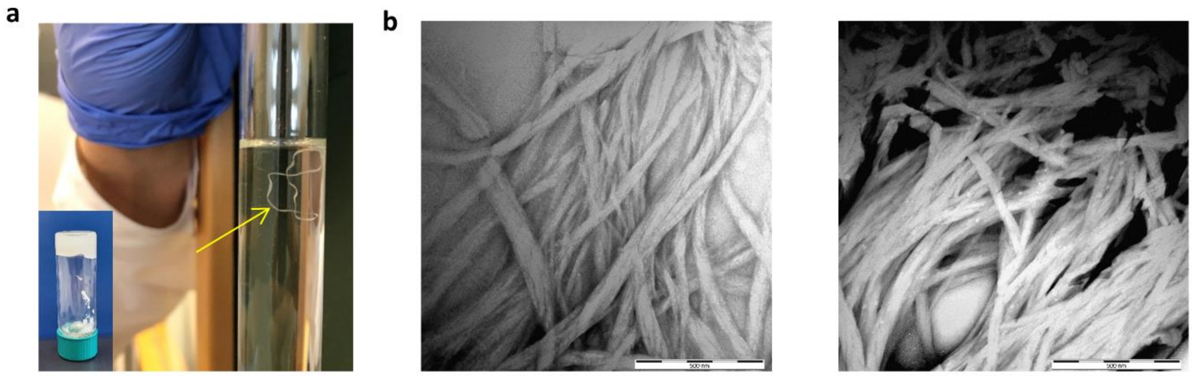

Supplementary Figure 5 | a, Fibers of **M_1** growing on the solvent-air interface inside a test tube after flash column chromatography. Inset shows a gel formed by 2 wt.% solution of **M_1** in $CHCl_3$. **b**, TEM images of **M_1** fibers on a carbon-coated copper grid, negatively stained with uranyl acetate (scale bars: 500 nm).

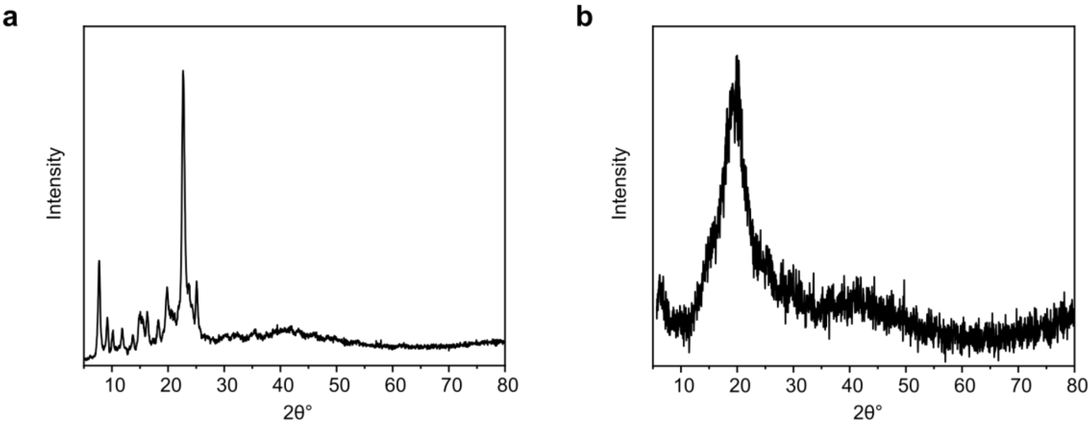

Supplementary Figure 6 | a, PXRD pattern for **$M_1^{Polymer}$** fibers obtained from 20 mM solution in $CHCl_3$. **b**, PXRD pattern for **$M_3^{Polymer}$** fibers obtained from 1.2 mM solution in cyclohexane/chloroform 84:16 v/v via seeded growth. In both cases, solvents were removed under vacuum at 273 K and polymers were finely grinded in a mortar prior to measurement.

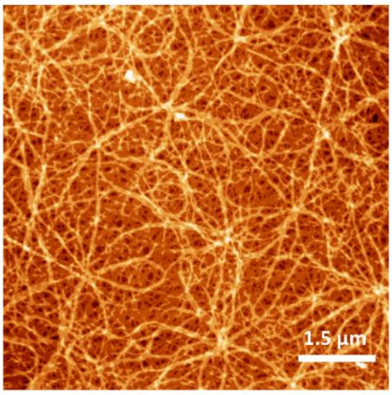

Supplementary Figure 7 | AFM height image of a cooled solution of **M_3** (275 K, 1.2 mM, cyclohexane/chloroform 84:16 v/v) spin-coated on a silicon wafer.

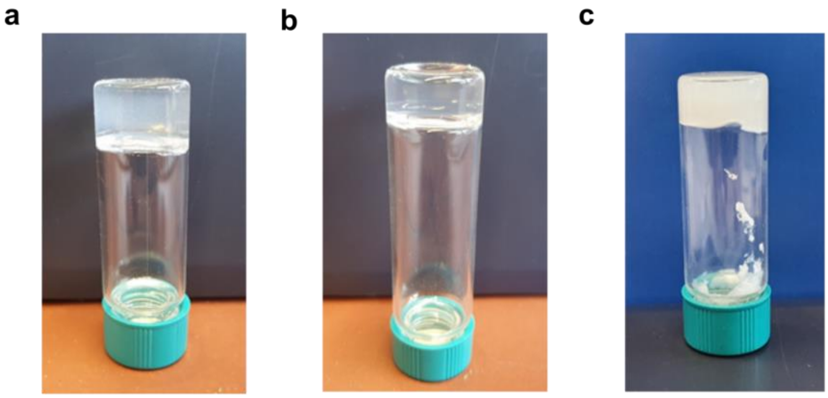

Supplementary Figure 8 | a, Gel of $\mathbf{M_3}$ (5 mM, cyclohexane/chloroform 84:16 v/v, 293 K). **b**, Gel of $\mathbf{M_5}$ (5 mM cyclohexane/chloroform 95:5 v/v 293 K). **c**, Gel of $\mathbf{M_1}$ (17 mM in $CHCl_3$ 293 K).

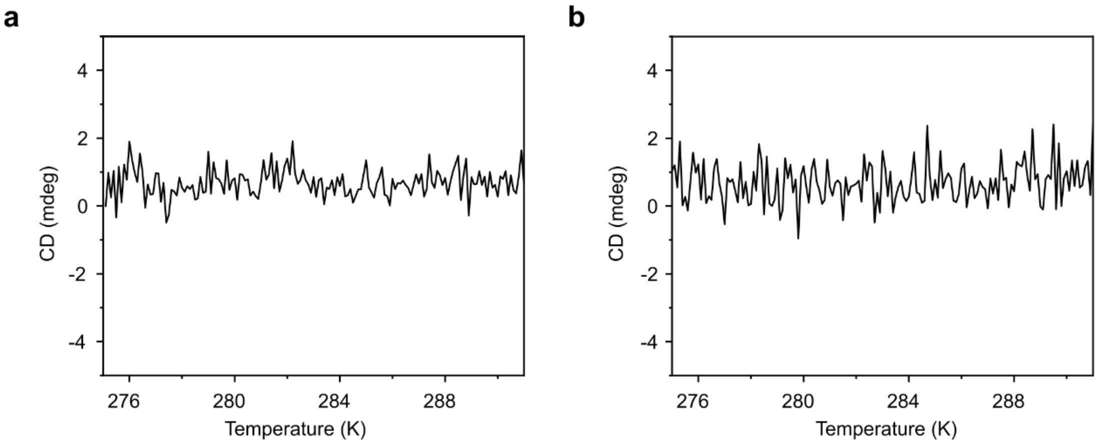

Supplementary Figure 9 | Plots of ellipticity at 260 nm against temperature for the cooling of (**a**) $\mathbf{Ref_2}$ and (**b**) ***Me*****M3**. Conditions: 1.2 mM, CH/$CHCl_3$ (84:16 v/v), 0.5 K min^{-1}.

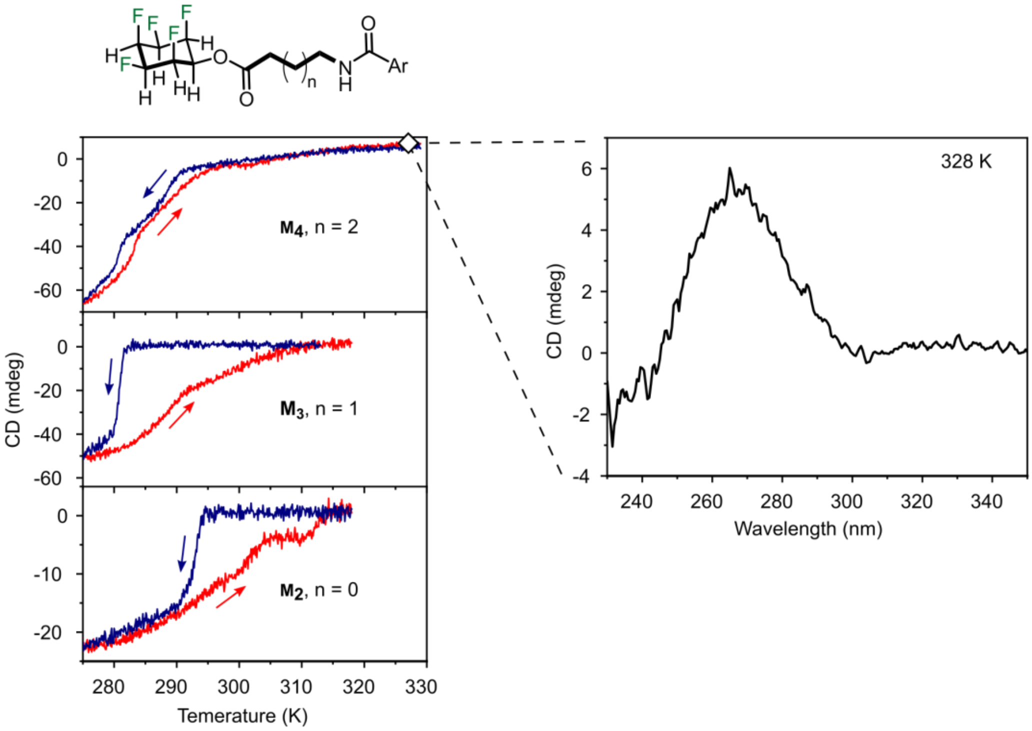

Supplementary Figure 10 | CD signal (260 nm) recorded for cooling (blue lines) and heating (red lines) of 1.2 mM solutions of **M4**, **M3** and **M2** in cyclohexane/chloroform mixture (84:16 v/v) at a rate of 0.5 K min^{-1} and CD spectrum of **M4** (1.2 mM, cyclohexane/chloroform 84:16 v/v) at 328 K.

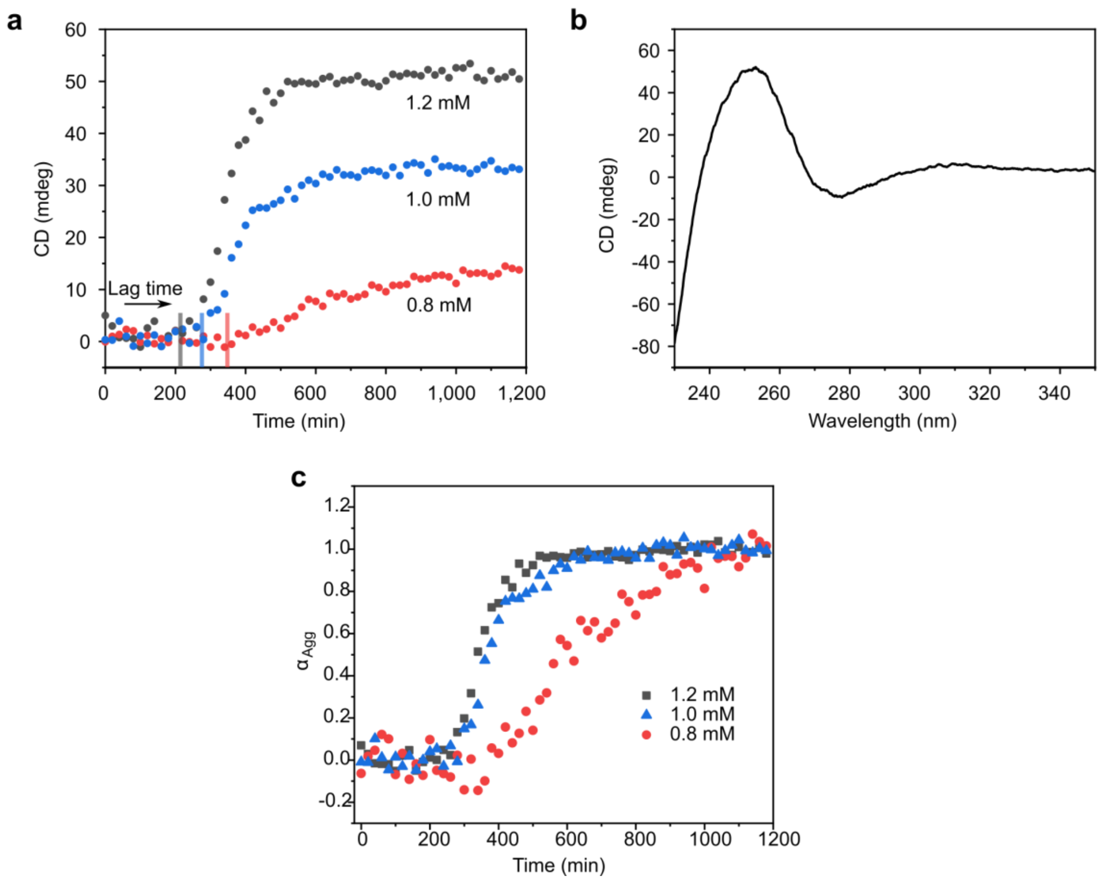

Supplementary Figure 11 | a, Time-course change of the CD signal (252 nm) for different concentrations of **M3** in cyclohexane/chloroform 84:16 (v/v) mixture at 293 K. **b**, CD spectrum of fully-aggregated 1.2 mM solution of **M3** in cyclohexane/chloroform 84:16 (v/v) mixture at 293 K. **c**, Normalized time-course change of the CD signal. Spontaneous polymerization of **M3** in cyclohexane/chloroform 84:16 (v/v) mixture occurs after a lag time and exhibits a sigmoidal transition. The lag time of the spontaneous polymerization depends on the initial concentration of **M3**, namely the time lag becomes shorter upon increasing the initial concentration of **M3**. This result indicates that no off-pathway aggregates are involved into kinetic stabilization of **M3**. Interestingly, during spontaneous polymerization at 293 K, we observed the formation of a different polymorph, which we did never observe in the seeded living polymerization.

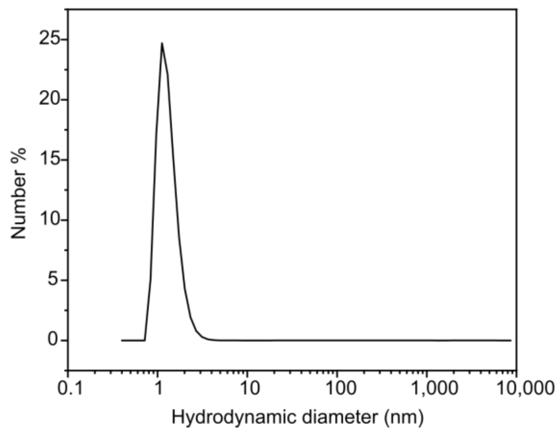

Supplementary Figure 12 | DLS profile of 1.2 mM solution of **M3** in cyclohexane/chloroform 84:16 (v/v) mixture at 293 K.

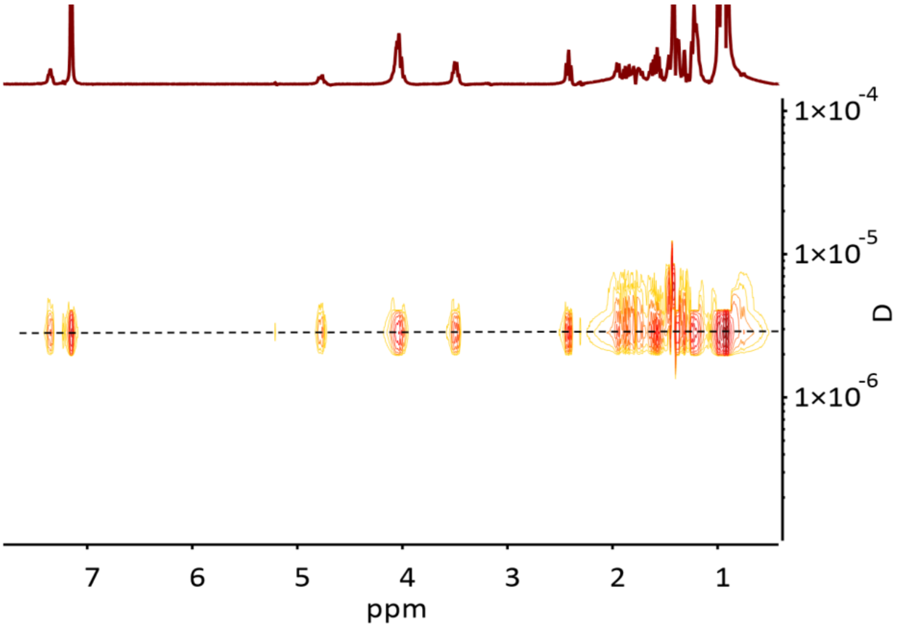

Supplementary Figure 13 | DOSY NMR of **Ref$_2$** (20 mM) in cyclohexane-*d$_{12}$*/chloroform-*d* 84:16 v/v mixture at 295 K. Diffusion coefficients are represented in $cm^2\ s^{-1}$. Black dotted line corresponds to D = $2.9\times10^{-10}\ m^2\ s^{-1}$.

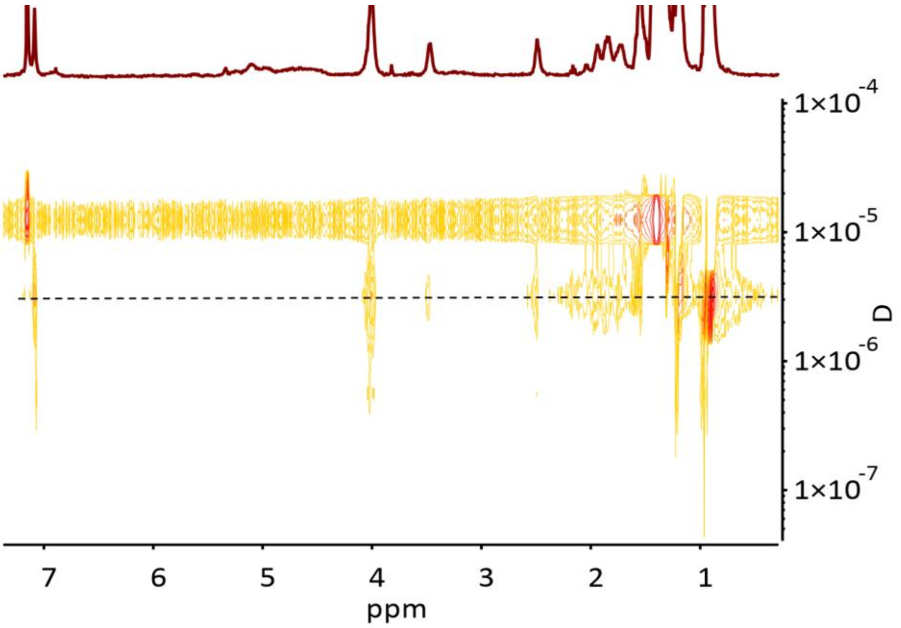

Supplementary Figure 14 | DOSY NMR of **M$_3$** (1.2 mM) in cyclohexane-*d$_{12}$*/chloroform-*d* 84:16 v/v mixture at 295 K. Diffusion coefficients are represented in $cm^2\ s^{-1}$. Black dotted line corresponds to D = $3.3\times10^{-10}\ m^2\ s^{-1}$. Please note not all signals can be clearly seen at this concentration due to limited resolution of the NMR machine.

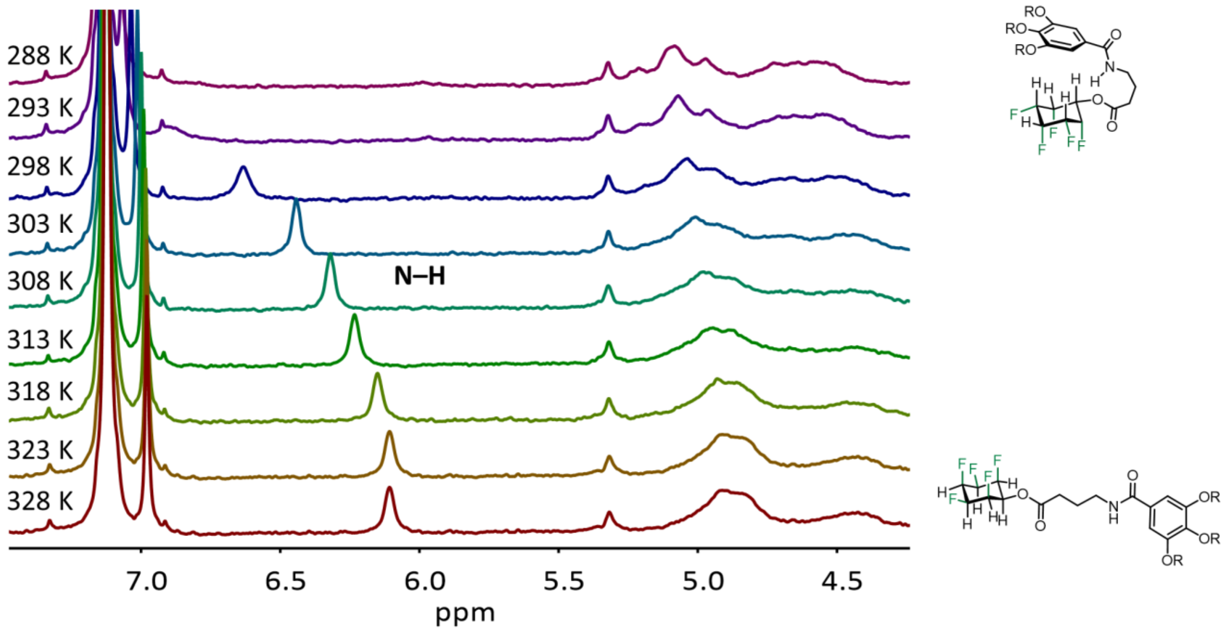

Supplementary Figure 15 | Partial VT ^{1}H NMR (500 MHz) spectra of **M$_3$** (1.2 mM) in cyclohexane-d_{12}/chloroform-*d* 84:16 v/v mixture.

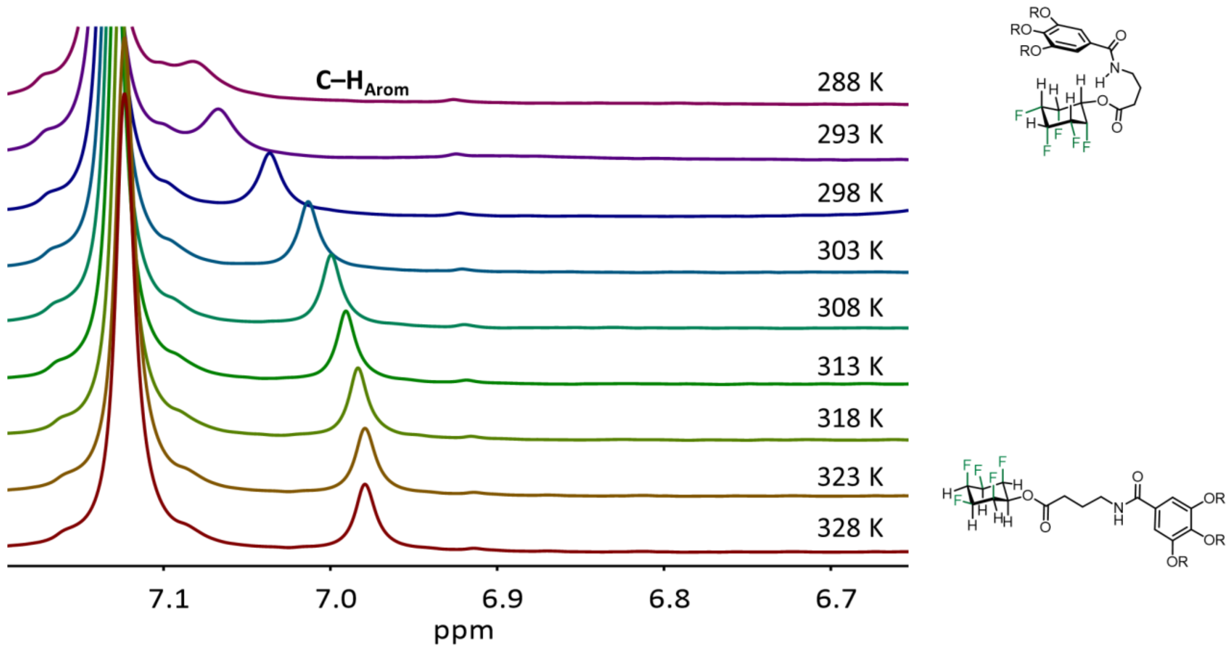

Supplementary Figure 16 | Partial VT ^{1}H NMR (500 MHz) spectra of **M$_3$** (1.2 mM) in cyclohexane-d_{12}/chloroform-*d* 84:16 v/v mixture. Aromatic region.

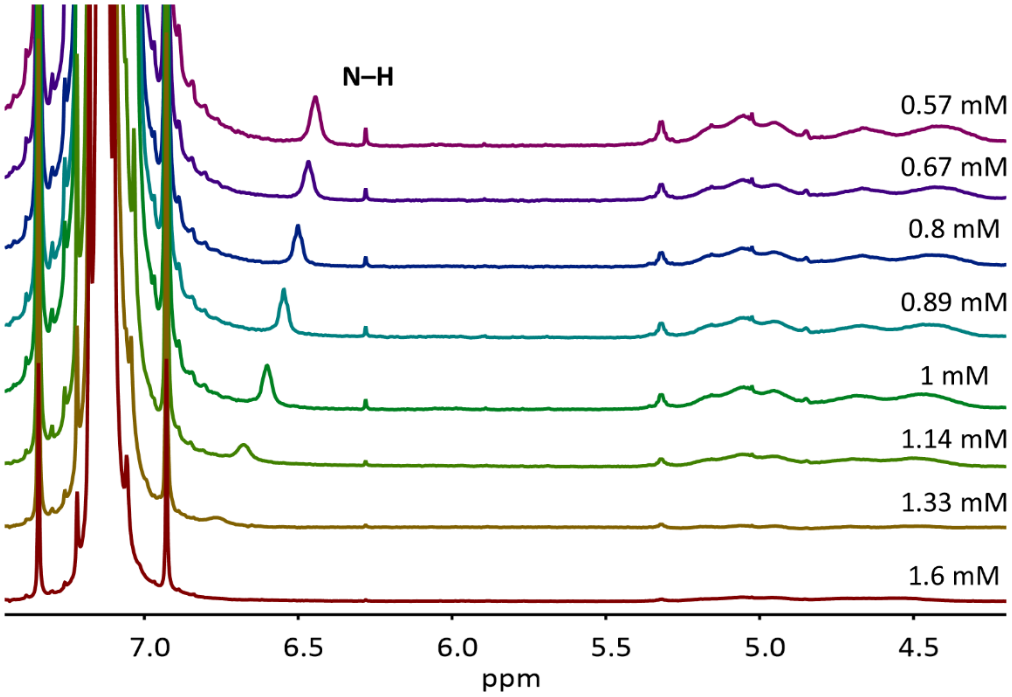

Supplementary Figure 17 | Concentration dependent ^{1}H NMR (400 MHz, 295 K) spectra of **M_3** in cyclohexane-d_{12}/chloroform-*d* 84:16 v/v mixture. Small changes of δ(N–H) were observed indicating low aggregation in solution.

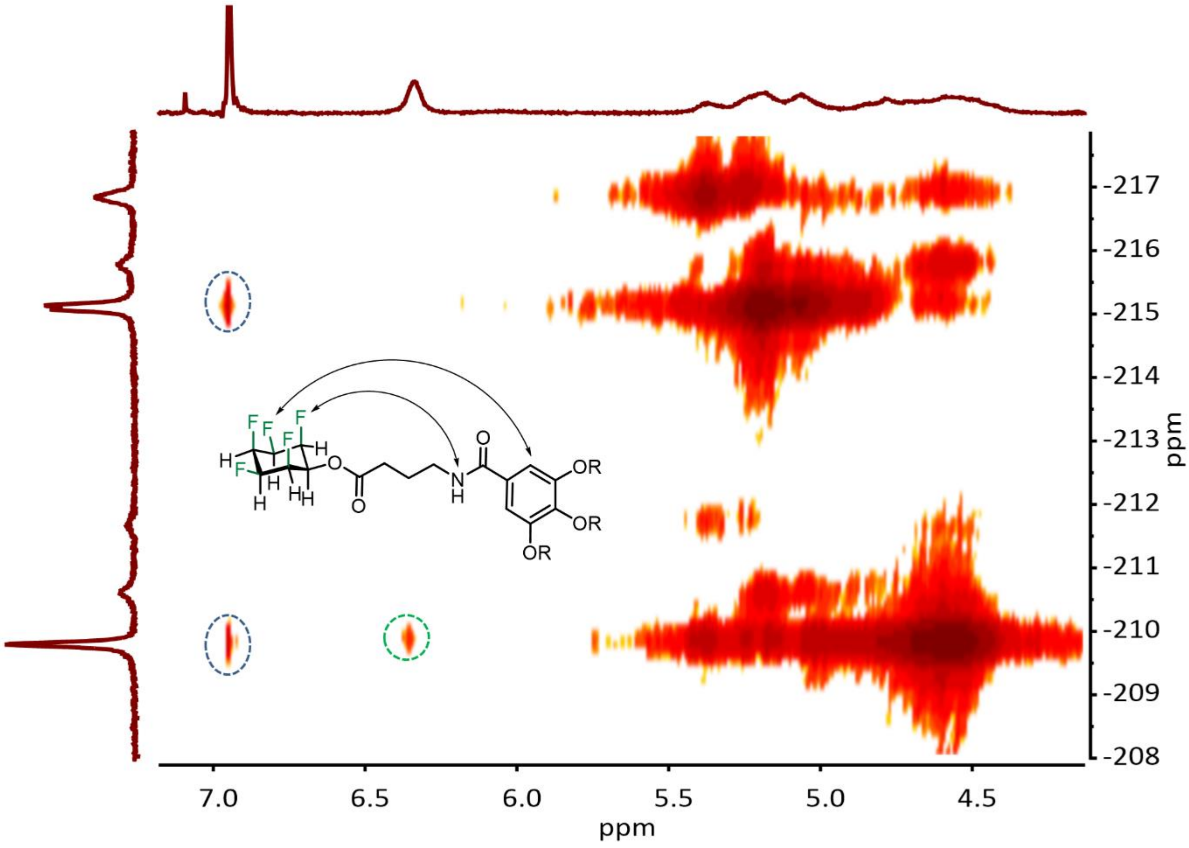

Supplementary Figure 18 | ^{1}H-^{19}F HOESY spectrum of 35 mM solution of **M_1** in $CDCl_3$ at 295 K.

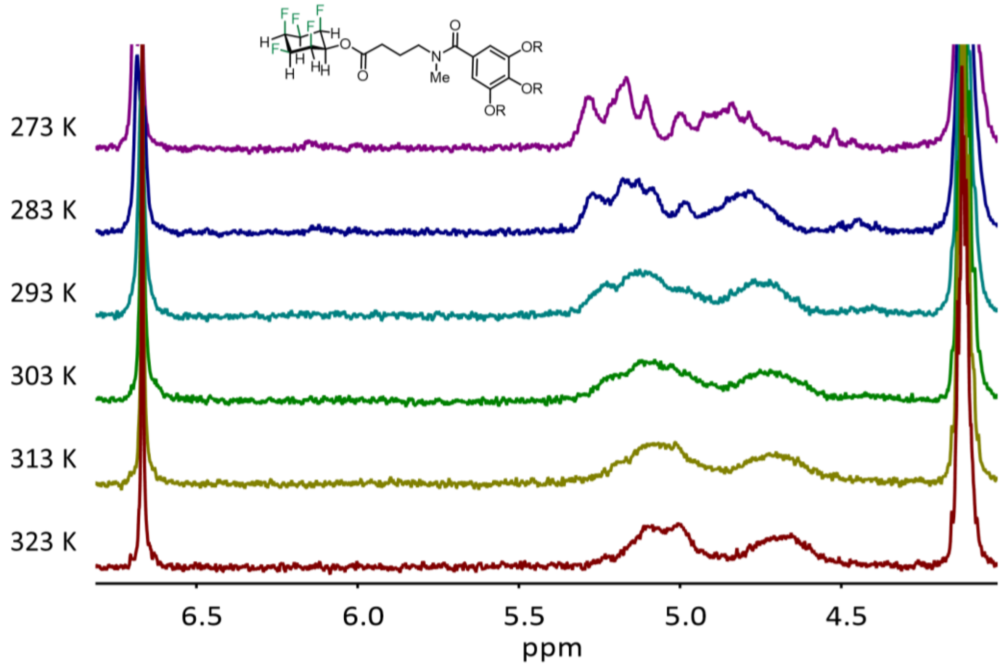

Supplementary Figure 19 | Partial VT ^{1}H NMR (500 MHz, 295 K) spectra of **MeM3** (1.2 mM) in cyclohexane-d_{12}/chloroform-*d* 84:16 v/v mixture.

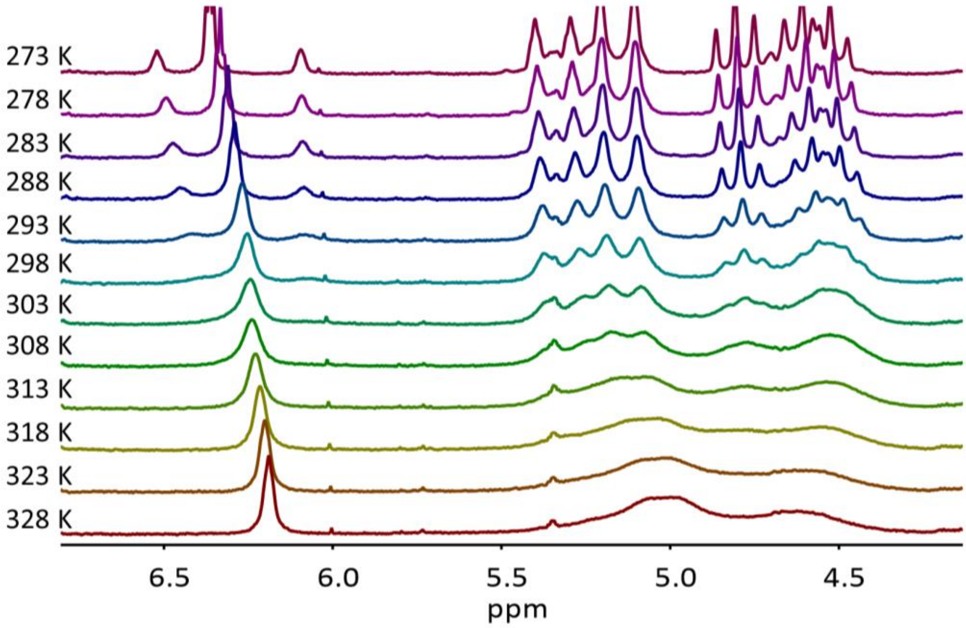

Supplementary Figure 20 | Partial VT ^{1}H NMR (500 MHz) spectra of **M3** (15 mM) in chloroform-*d*.

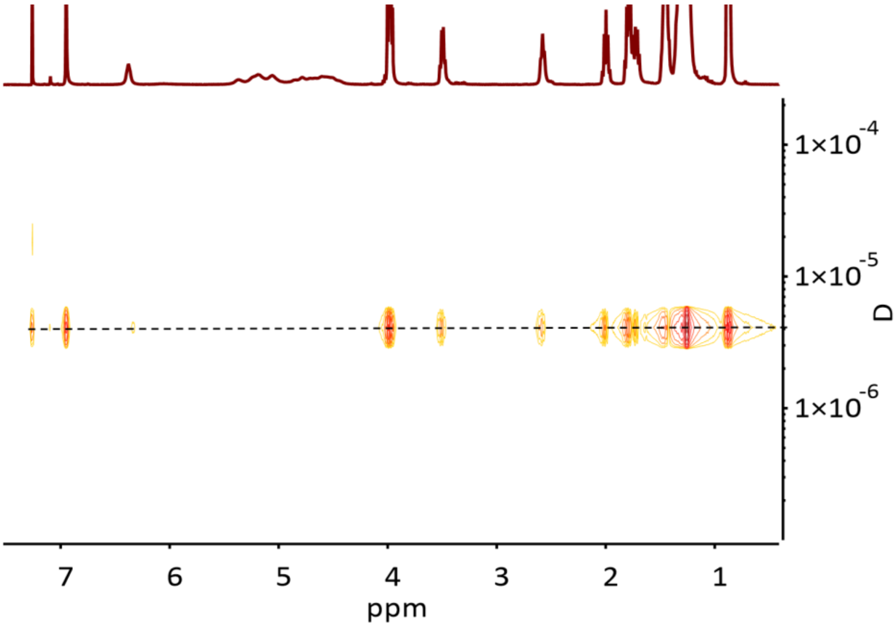

Supplementary Figure 21 | DOSY NMR of **M1** (25 mM) in $CDCl_3$ at 295 K. Diffusion coefficient are represented in $cm^2\ s^{-1}$. Black dotted line corresponds to D = $4.2\times10^{-10}\ m^2\ s^{-1}$.

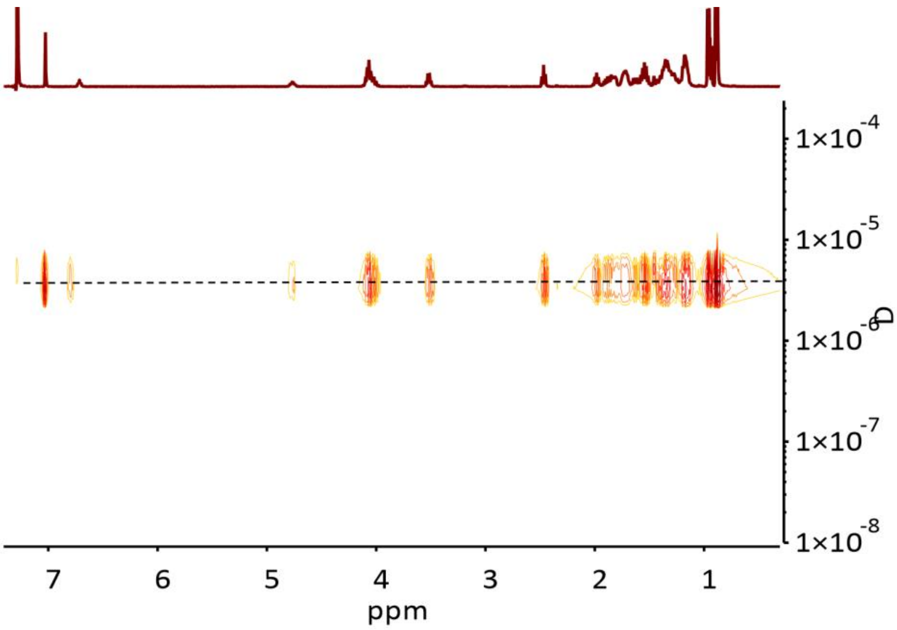

Supplementary Figure 22 | DOSY NMR of **Ref2** (25 mM) in $CDCl_3$ at 295 K. Diffusion coefficient are represented in $cm^2\ s^{-1}$. Black dotted line corresponds to D = $4\times10^{-10}\ m^2\ s^{-1}$.

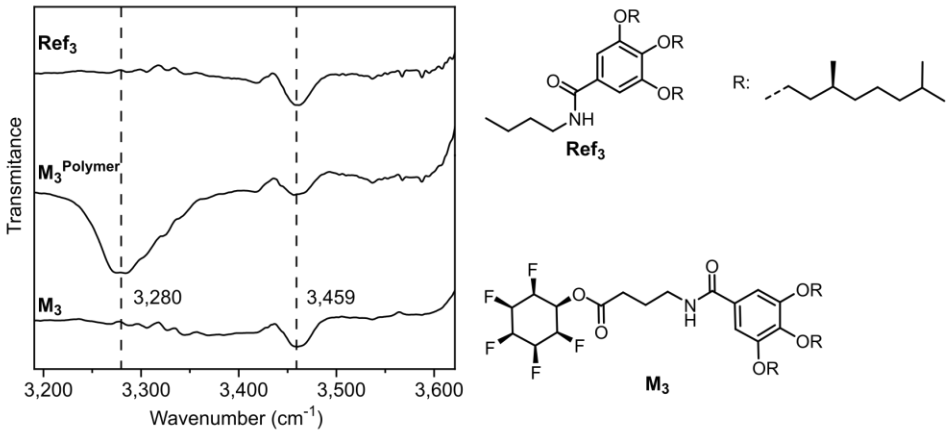

Supplementary Figure 23 | FT-IR spectra of N-H stretching vibration of 1.2 mM solutions (cyclohexane/chloroform 84:16 v/v) of **Ref**$_3$, **M**$_3$$^{\mathbf{Polymer}}$, obtained via seeded growth, and **M**$_3$. The vibration at 3280 cm^{-1} corresponds to intermolecularly hydrogen bonded amide.

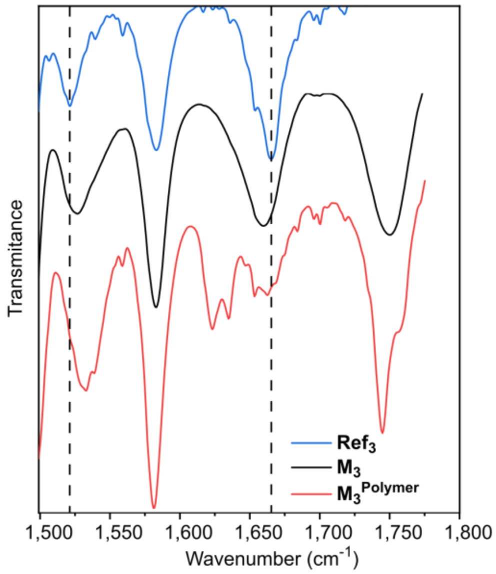

Supplementary Figure 24 | FT-IR spectra of the amide I (dashed line at 1665 cm^{-1}, C=O stretching vibration) and amide II (dashed line at 1521 cm^{-1}, NH in plane bend and the CN stretching vibration) of 1.2 mM solutions (cyclohexane/chloroform 84:16 v/v) of **Ref**$_3$, **M**$_3$$^{\mathbf{Polymer}}$, obtained via seeded growth, and **M**$_3$. The small shift is observed for both amide bends for **M**$_3$ and **M**$_3$$^{\mathbf{Polymer}}$ in comparison to **Ref**$_3$, lacking all-*cis* $C_6H_6F_5$ group. The shift of amide I band can be a result of CH···π interaction between all-*cis* $C_6H_6F_5$ group and aromatic ring, which directly influences the conjugation between the amide and aromatic ring.

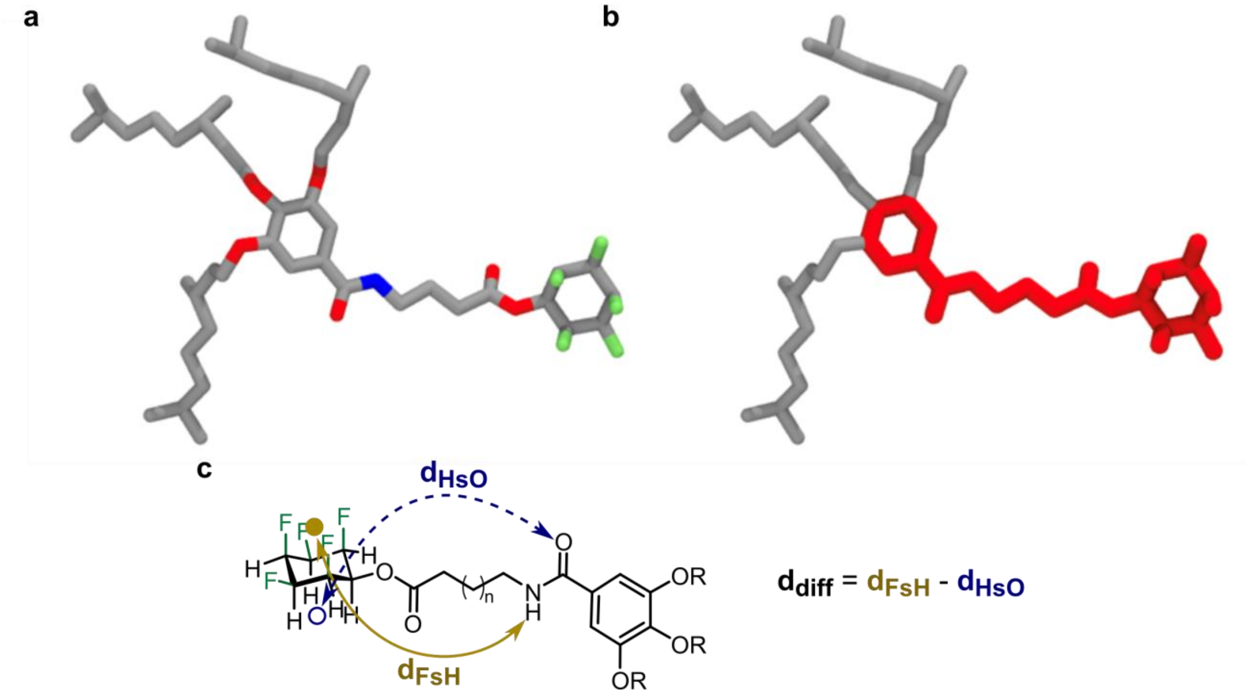

Supplementary Figure 25 | a, Licorice representation of **M3** monomer (hydrogen atoms have been ommited for clarity). **b**, Partition of **M3** monomer into “core” (in red) and “tails” (grey). The radius of gyration of the monomer core is used to define CV2 used in the WT-MetaD simulations. **c**, CV1 is defined as difference between d_{FsH} and d_{HsO} distances. Distances are measured between the centres of mass of the cyclohexane fluorides and the amidic hydrogen (d_{FsH}) and the centres of mass of the cyclohexane hydrogens and the amidic oxygen (d_{HsO}).

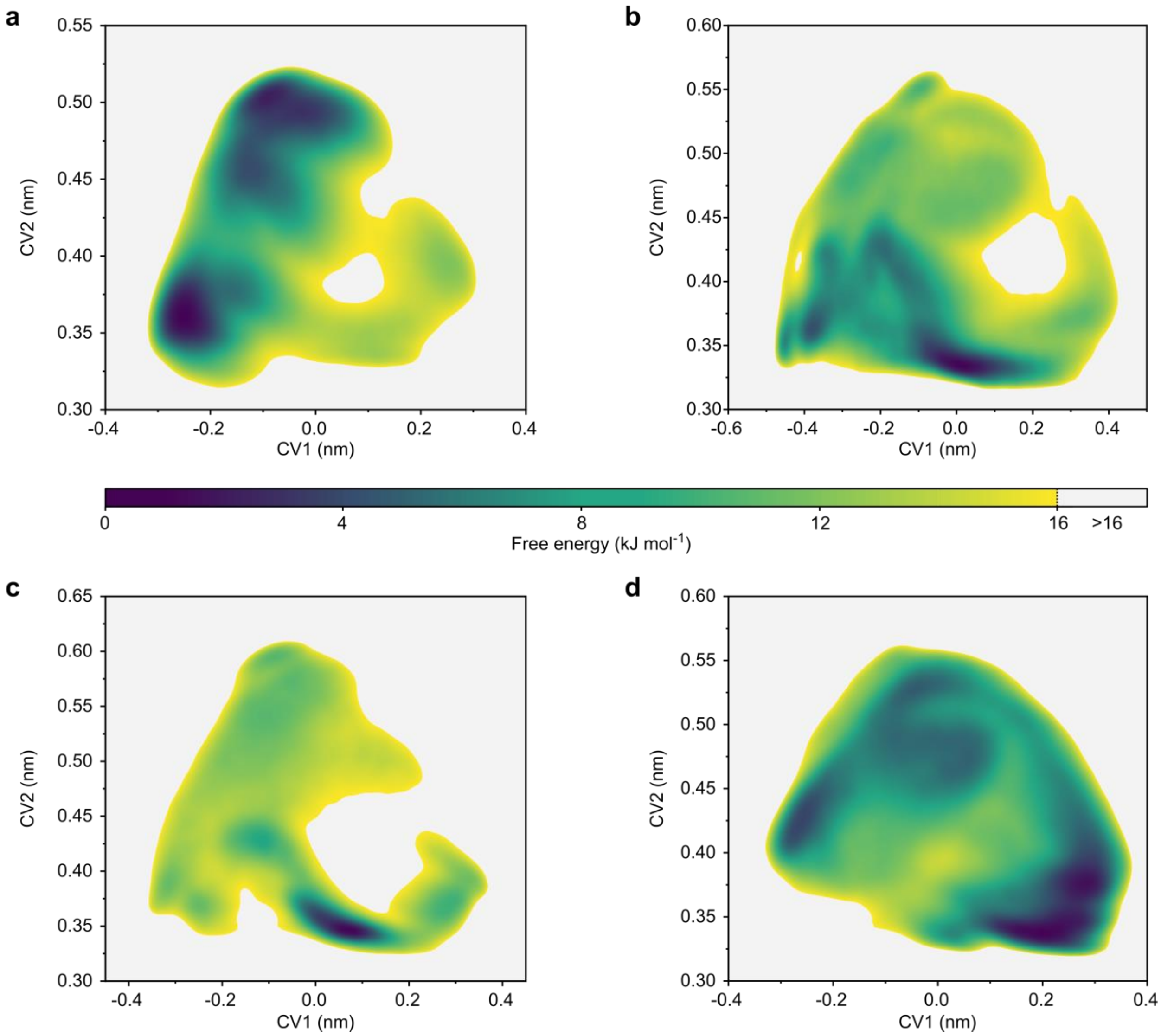

Supplementary Figure 26 | Free energy surface of the monomers **M2** (**a**), **M3** (**b**), **M4** (**c**) and ***Me*M3** (**d**) conformational space estimated by MetaD enhancing the sampling along the difference of the distances between pentafluorocyclohexyl-Hs and amide oxygen and between the pentafluorocyclohexyl-Fs and the amide-nitrogen (abscissa, except for the case of ***Me*M3** where the methyl-carbon was considered), and the radius of gyration of the core of each monomer (ordinate).

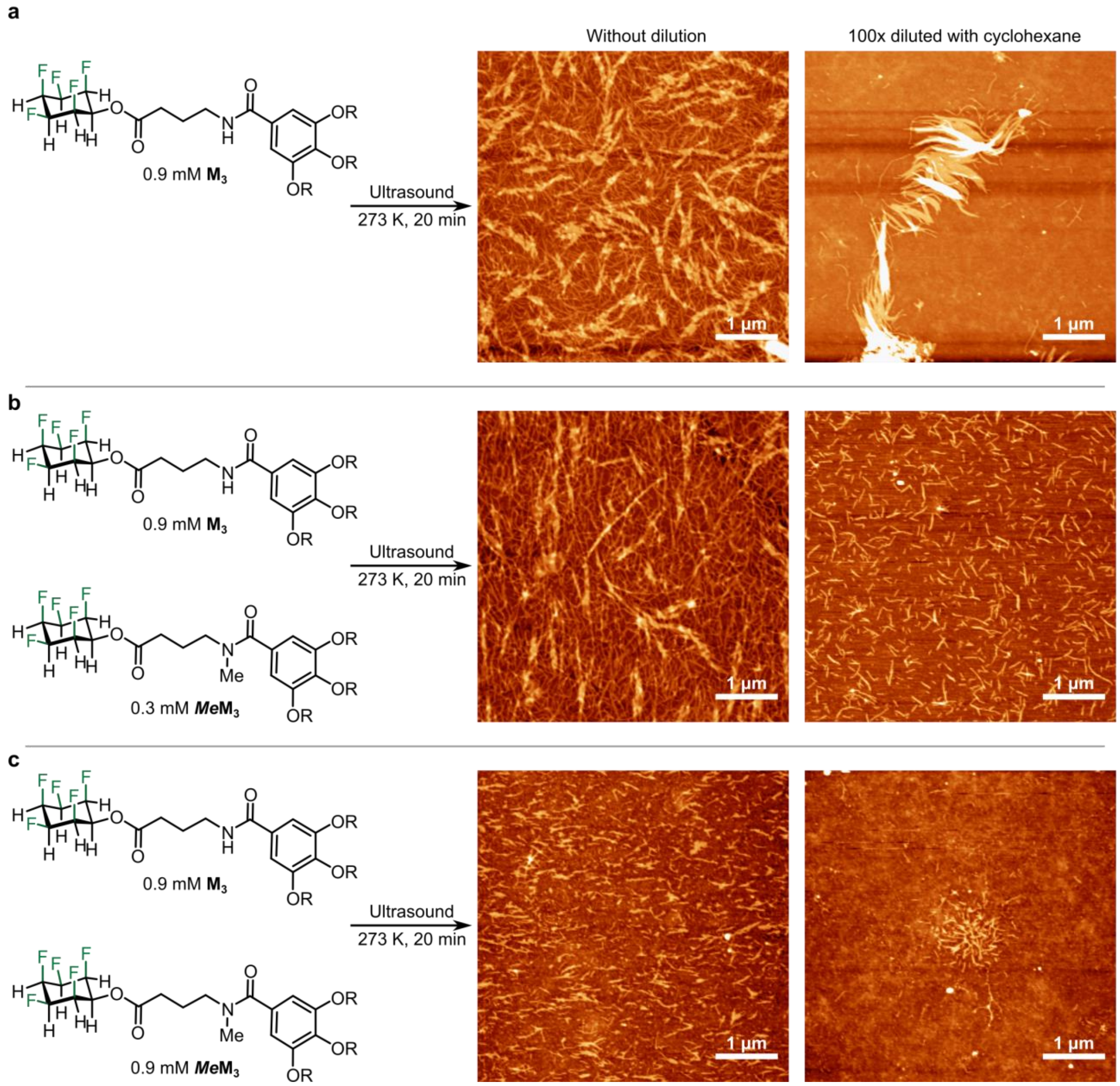

Supplementary Figure 27 | a, Fibers of $\mathbf{M_3}$ obtained by applying ultrasound for 20 min at 273 K to 0.9 mM solution of $\mathbf{M_3}$ in cyclohexane/chloroform mixture 84:16 (v/v). AFM height images of fibers spin-coated on the silicon wafer directly from the solution (without dilution) and diluted 100x with cyclohexane. In former case, most of the material is deposited in the form of bundles of long non-uniform (ca. 1 µm) fibers. **b**, $\mathbf{M_3}^{\mathbf{Seed}}$ obtained by applying ultrasound for 20 min at 273 K to the mixture of $\mathbf{M_3}$ (0.9 mM) and $\boldsymbol{Me}\mathbf{M_3}$ (0.3 mM) in cyclohexane/chloroform solvent mixture 84:16 (v/v). AFM height images show well-defined short fibers after dilution. **c**, Fibers of $\mathbf{M_3}$ obtained by applying ultrasound for 20 min at 273 K to the mixture of $\mathbf{M_3}$ (0.9 mM) and $\boldsymbol{Me}\mathbf{M_3}$ (0.9 mM) in cyclohexane/chloroform solvent mixture 84:16 (v/v). AFM height images of fibers spin-coated on the silicon wafer indicate the formation of aggregates with poorly defined morphology. In all cases, AFM samples were prepared in an identical way, using the same substrate (silicone).

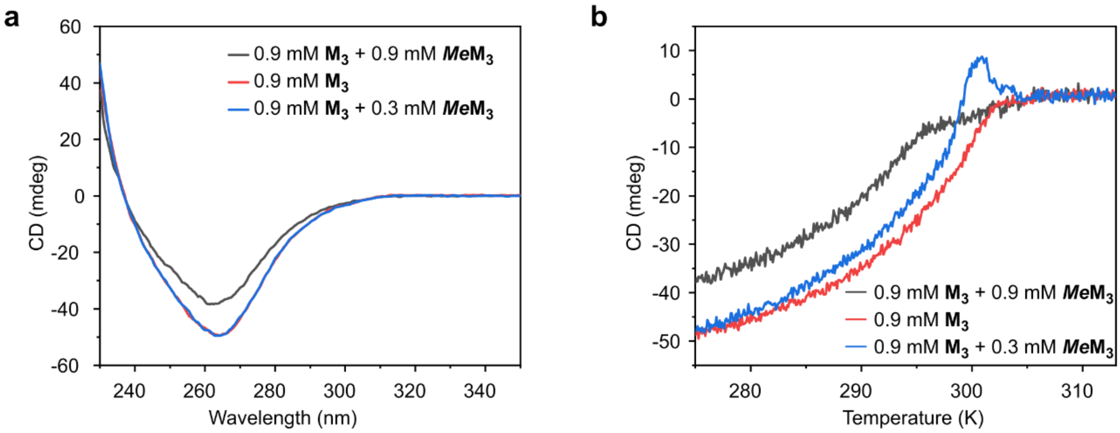

Supplementary Figure 28 | a, CD spectra (84:16 v/v cyclohexane/chloroform, 275 K) of “seeds” obtained by 1) sonication of 0.9 mM solution of **M3** for 20 min at 273 K (red line); 2) sonication of the mixture of ***Me*M3** (0.3 mM) and **M3** (0.9 mM) for 20 min at 273 K (blue line); 3) sonication of the mixture of ***Me*M3** (0.9 mM) and **M3** (0.9 mM) for 20 min at 273 K (black line). **b**, Melting CD curves (260 nm, 84:16 v/v cyclohexane/chloroform, 0.5 K/min) of “seeds” obtained by 1) sonication of 0.9 mM solution of **M3** for 20 min at 273 K (red line); 2) sonication of the mixture of ***Me*M3** (0.3 mM) and **M3** (0.9 mM) for 20 min at 273 K (blue line); 3) sonication of the mixture of ***Me*M3** (0.9 mM) and **M3** (0.9 mM) for 20 min at 273 K (black line).

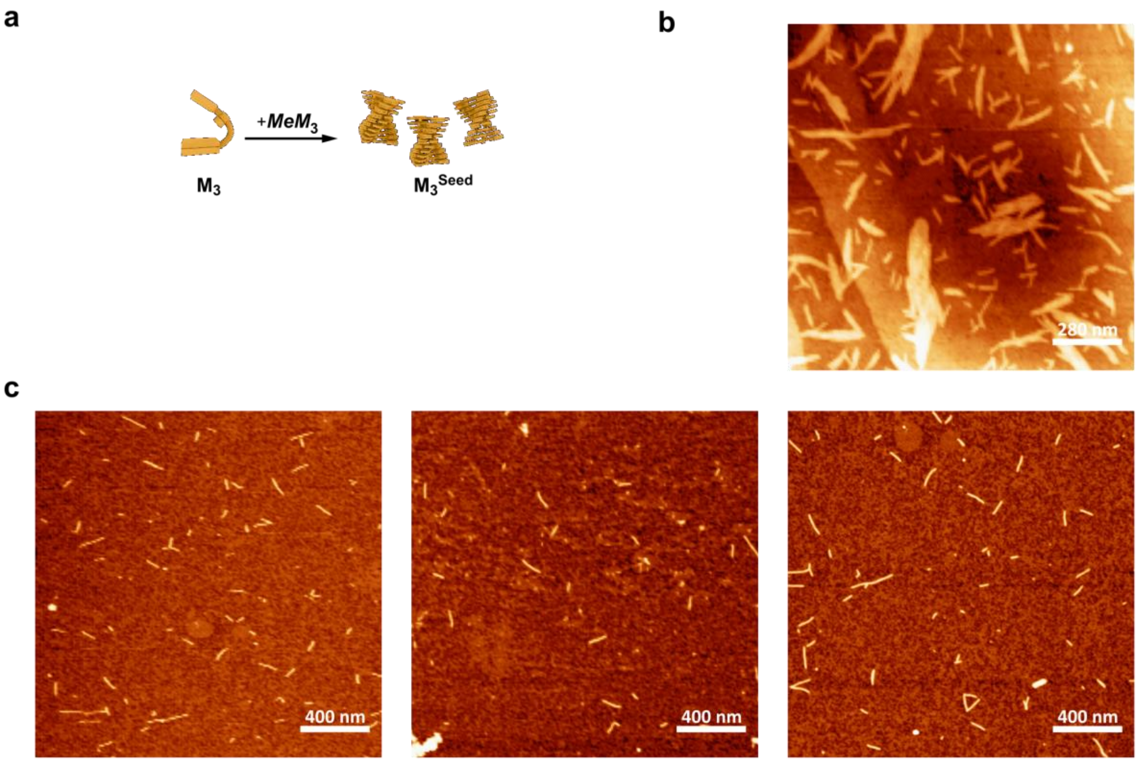

Supplementary Figure 29 | a. Schematic illustration of $\mathbf{M3}^{\mathbf{Seed}}$ preparation. **b.** AFM height image of $\mathbf{M3}^{\mathbf{Seed}}$, spin-coated (1 µL, 8000 rpm) on a HOPG wafer before 100x dilution with cyclohexane (scale bar 280 nm). **c.** Representative AFM height images of $\mathbf{M3}^{\mathbf{Seed}}$, spin-coated on a silicon wafer after 100x dilution with cyclohexane.

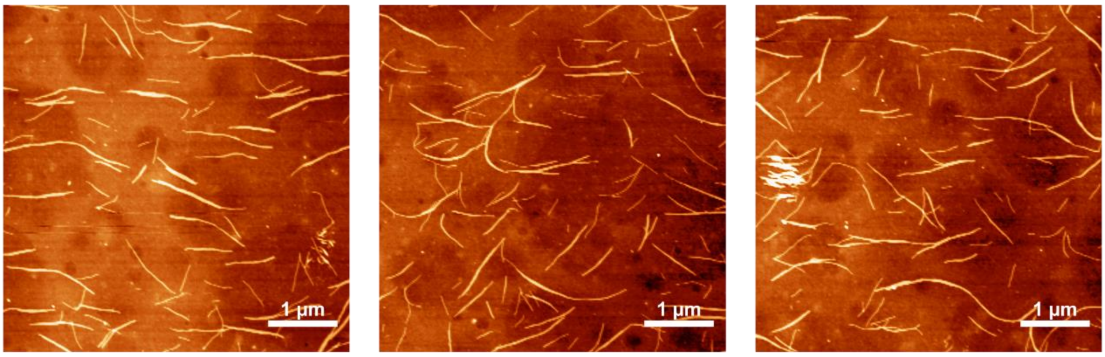

Supplementary Figure 30 | AFM height images of supramolecular polymers obtained using “seeds” obtained by sonication of 0.9 mM solution of $\mathbf{M_3}$ (84:16 v/v cyclohexane/chloroform) for 20 min at 273 K. $\mathbf{M_3}$/**Seed** = 3:1.

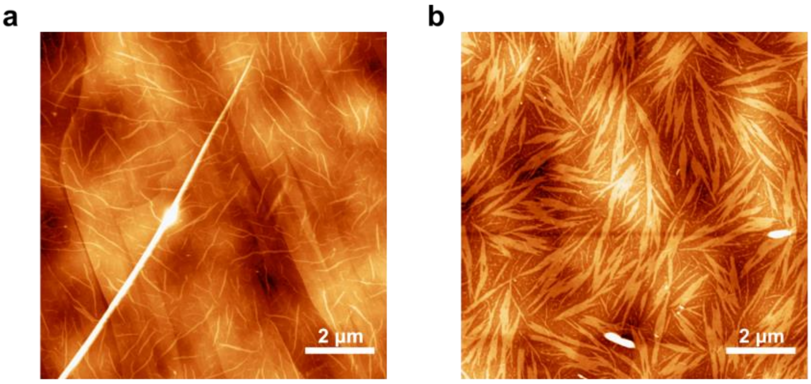

Supplementary Figure 31 | AFM height images of $\mathbf{M_3}^{\mathbf{Polymer}}$ obtained with 5:1 [$\mathbf{M_3}$]/[$\mathbf{M_3}^{\mathbf{Seed}}$] (v/v) ratio spin-coated (<1 µL, 8000 rpm) before 100x dilution on (**a**) HOPG and (**b**) silicon wafers.

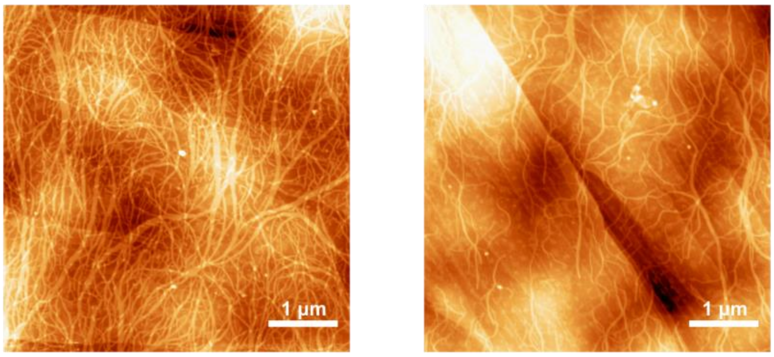

Supplementary Figure 32 | AFM height images of $\mathbf{M_3}^{\mathbf{Polymer}}$ obtained with 10:1 [$\mathbf{M_3}$]/[$\mathbf{M_3}^{\mathbf{Seed}}$] (v/v) ratio spin-coated (<1 µL, 8000 rpm) before 100x dilution on HOPG.

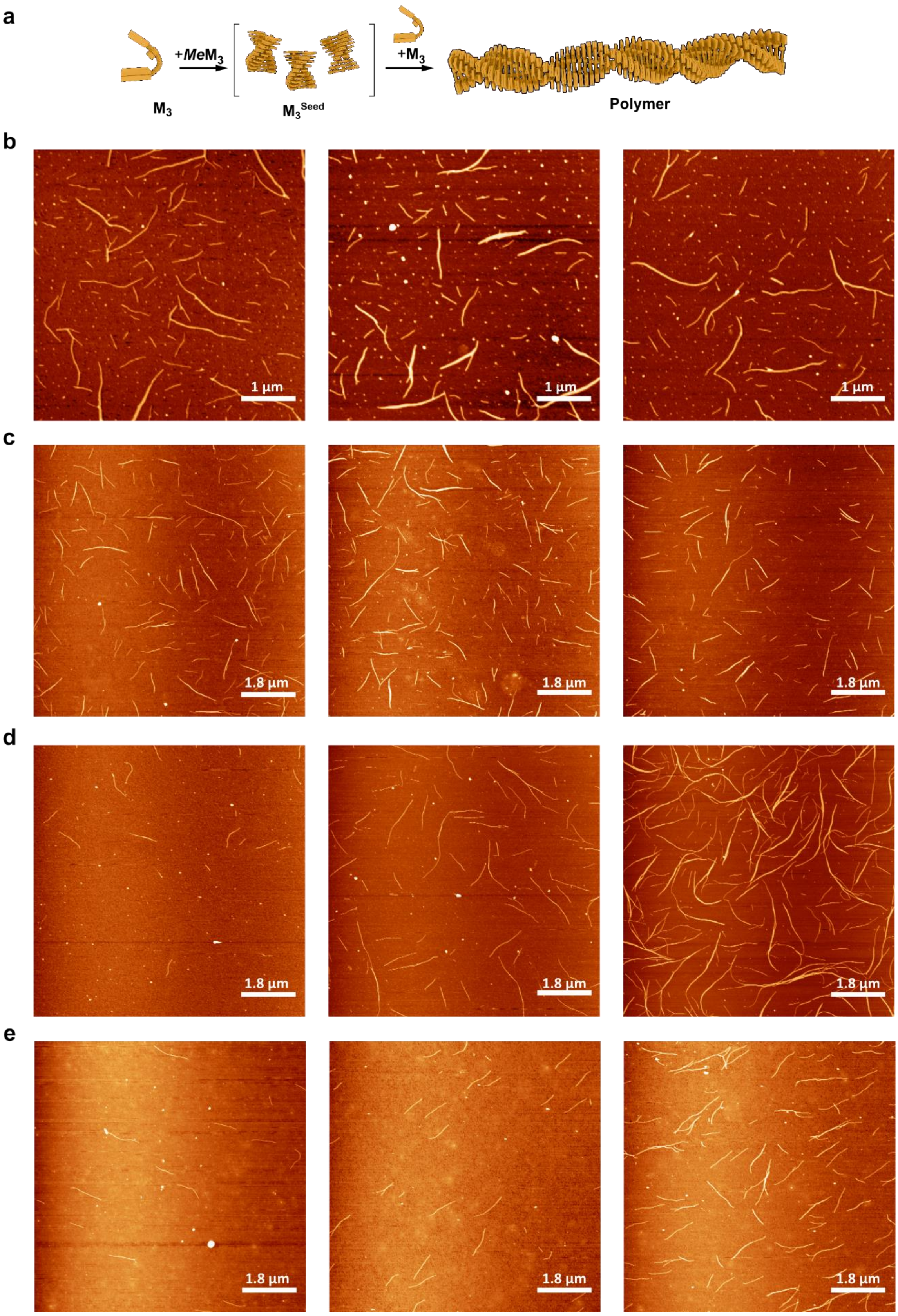

Supplementary Figure 33 | a, Schematic illustration of seeded living supramolecular polymerization. AFM height images of $\mathbf{M3}^{\mathbf{Polymer}}$ obtained with (**b**) 3:1 (**c**) 5:1, (**d**) 7:1 and (**e**) 10:1 [**M3**]/[$\mathbf{M3}^{\mathbf{Seed}}$] (v/v) ratio**.**

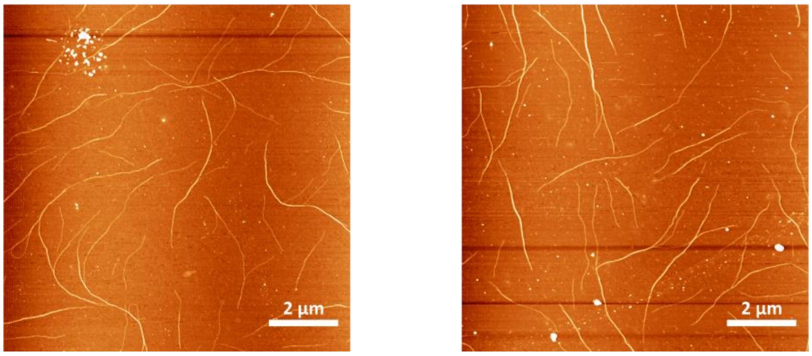

Supplementary Figure 34 | AFM height images of supramolecular polymers obtained with 20:1 [**M3**]/[**M3**^{Seed}] ratio**.**

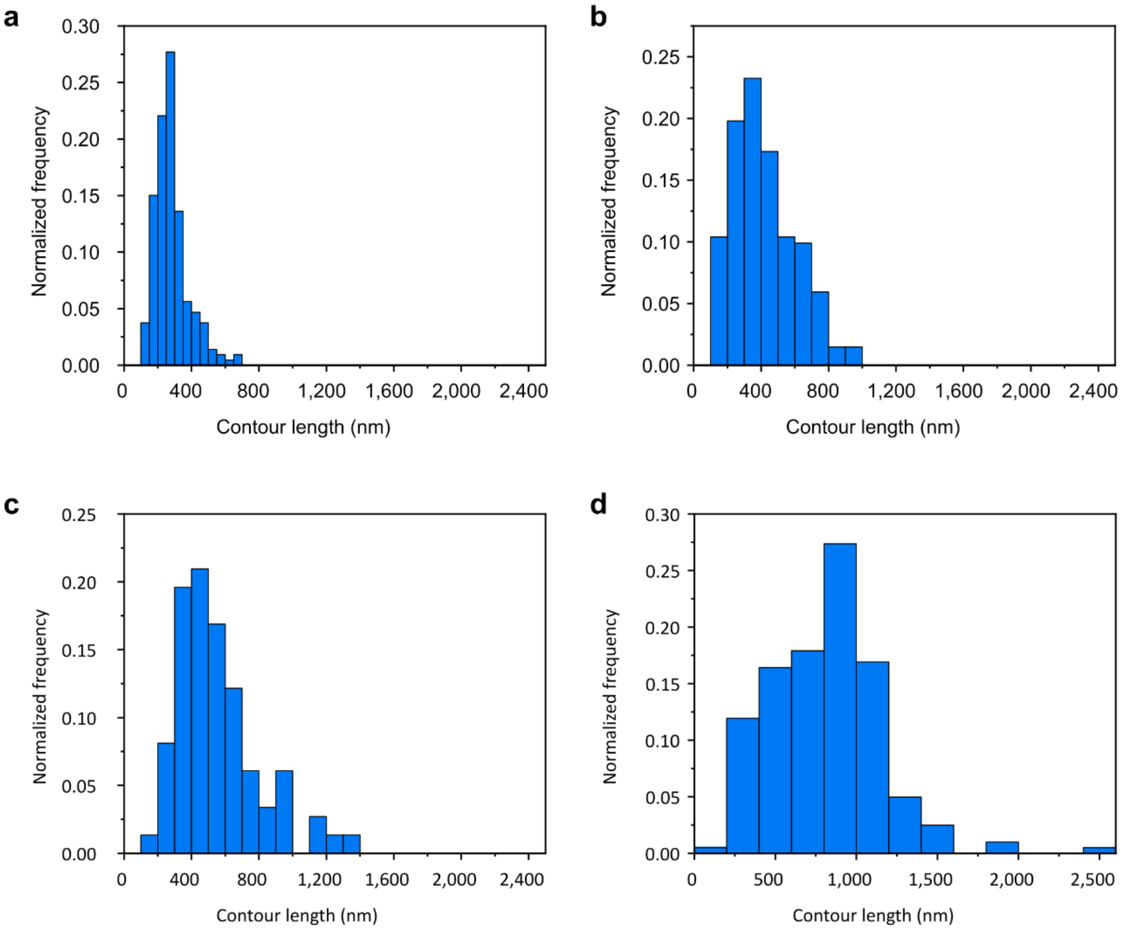

Supplementary Figure 35 | Contour length distribution obtained from AFM images of supramolecular polymers obtained with different [**M3**]/[**M3**^{Seed}] ratios: (**a**) 3:1; (**b**) 5:1; (**c**) 7:1; (**d**) 10:1 v/v.

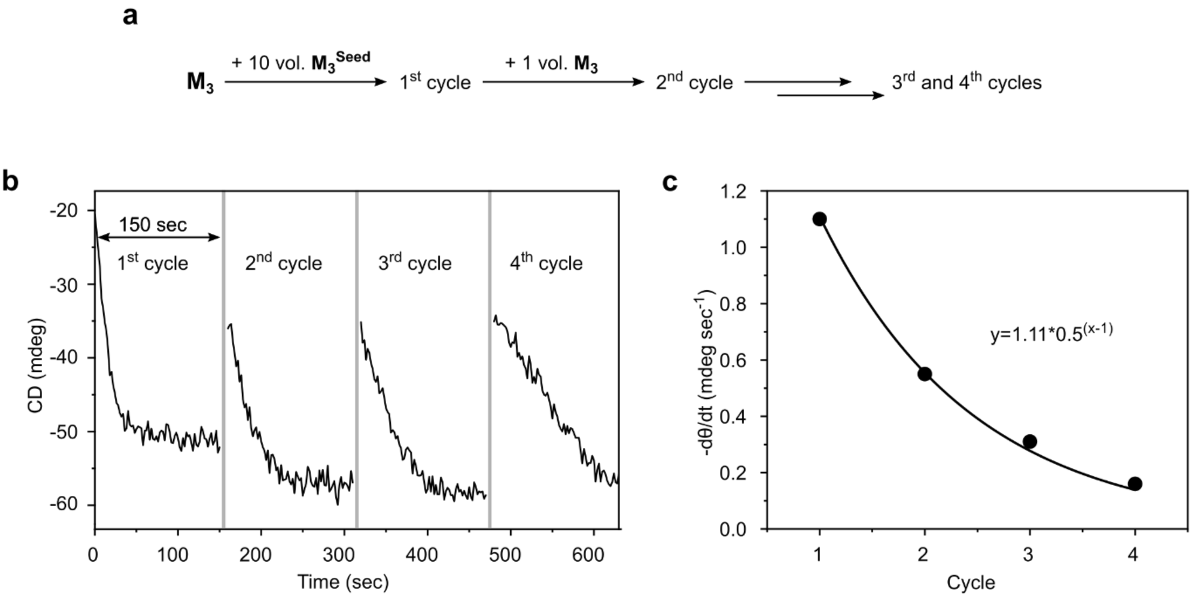

Supplementary Figure 36 | a, Schematic illustration of the experiment used to investigate the living character of the supramolecular polymerization of **M3**. **b**, Time-dependent CD (260 nm) changes during each polymerization cycle. **c**, Rate of polymerization as a function of the cycle number.

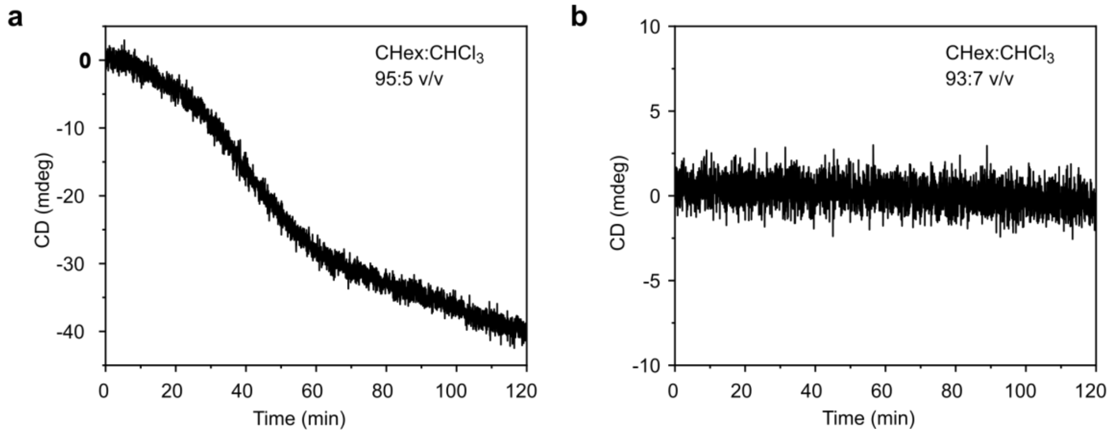

Supplementary Figure 37 | Time development of the CD signal (260 nm) of **M5** in (**a**) cyclohexane/chloroform 95:5 (v/v) and (**b**) cyclohexane/chloroform 93:7 (v/v) at 293 K. In cyclohexane/chloroform 95:5 (v/v) spontaneous polymerization starts in <10 min whereas in cyclohexane/chloroform 93:7 (v/v) monomer remains in a molecularly dissolved state for >1 h which is enough to carry out seeded living supramolecular polymerization. If necessary kinetic stability can be increased by increasing the fraction of chloroform.

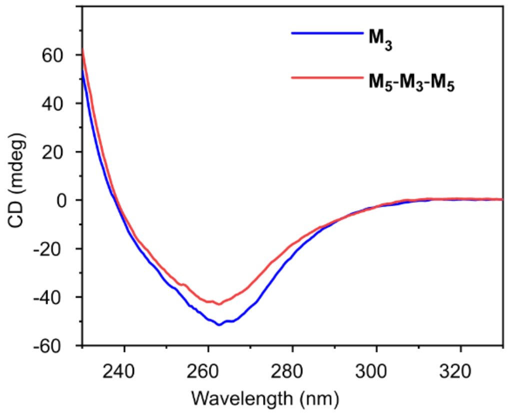

Supplementary Figure 38 | Blue - CD spectrum of **M3** homopolymer (c_{tot} = 1.2 mM, 84:16 v/v cyclohexane/chloroform, 293 K, [**M3**]/[**M3**^{Seed}] = 50:1 v/v). Red - CD spectrum of **M5-M3-M5** supramolecular block copolymer (c_{tot} = 1.2 mM, 293 K, [**M5**]/[**M3**^{Seed}] = 3:1 v/v).

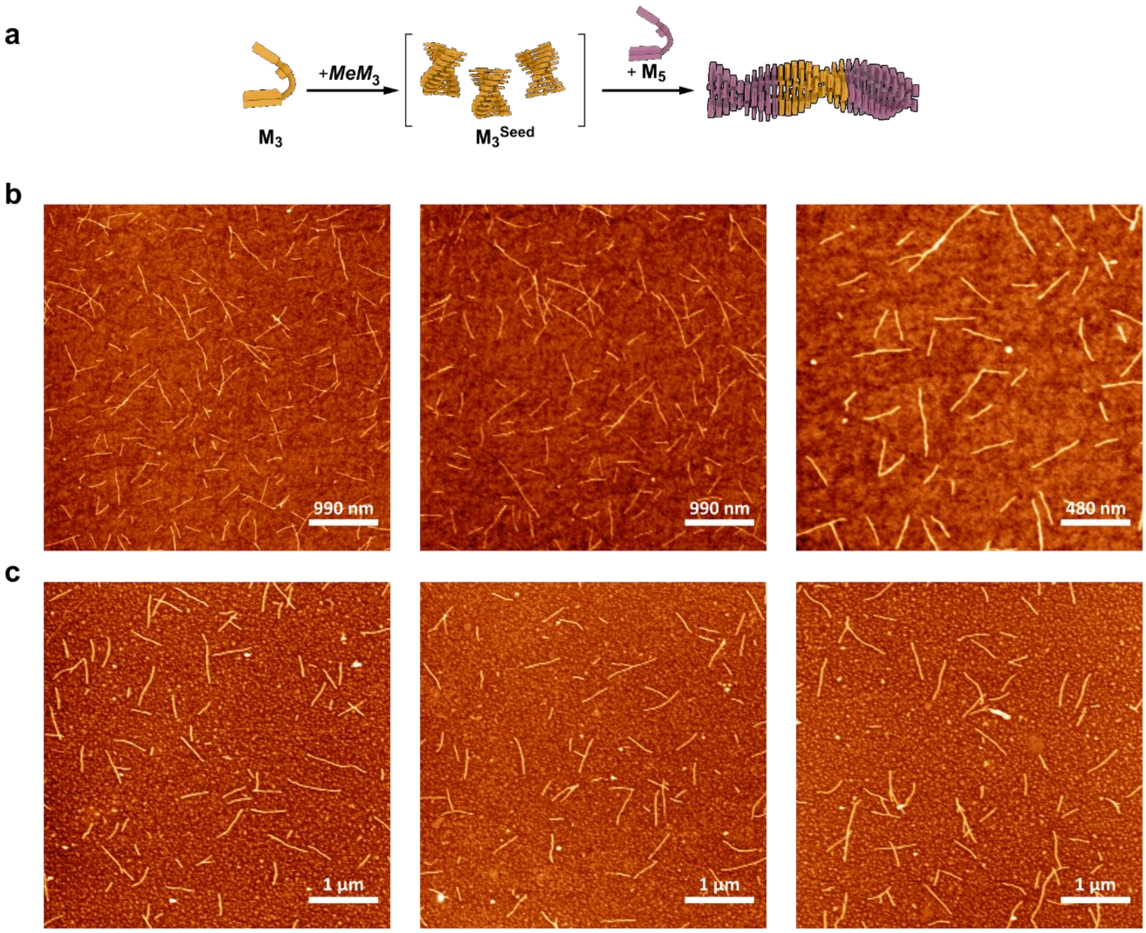

Supplementary Figure 39 | a, Schematic illustration of the experiment. Solution of **M5** (1.2 mM, cyclohexane/chloroform 93:7 v/v) was seeded with **M3**^{Seed} at 293 K. AFM height images of **M5-M3-M5** supramolecular block copolymer obtained with (**b**) 3:1 and (**c**) 5:1 [**M5**]/[**M3**^{Seed}] (v/v) ratio**.** The values for the **M3-M3-M3** homopolymer obtained with 3:1 [**M3**]/[**M3**^{Seed}] (v/v) ratio were 316 nm, 282 nm and 1.12. The values for the **M3-M3-M3** homopolymer obtained with 5:1 [**M3**]/[**M3**^{Seed}] (v/v) ratio were 499 nm, 418 nm and 1.19.

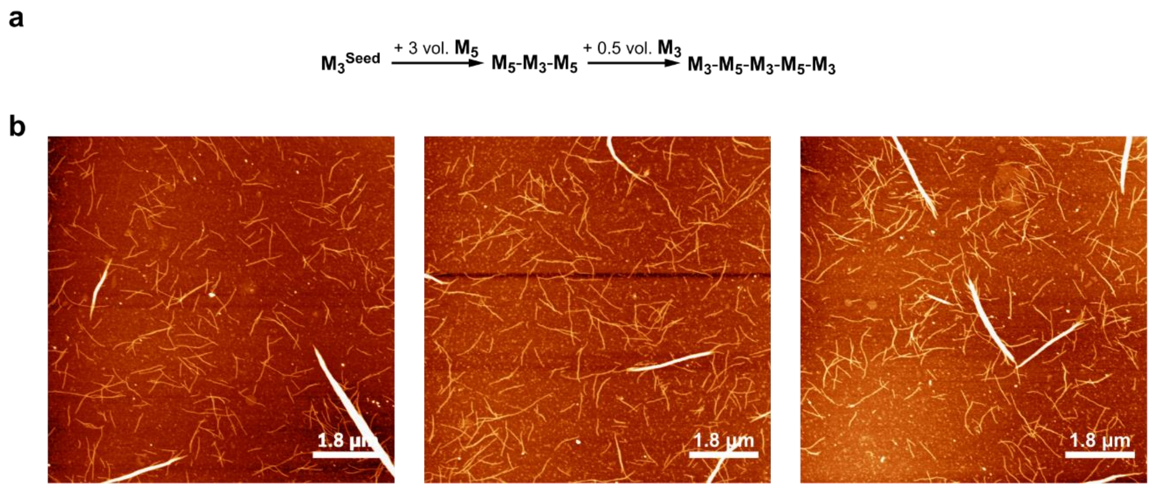

Supplementary Figure 40 | a, Schematic illustration of the experiment. Solution of **M**$_5$ (1.2 mM, cyclohexane/chloroform 93:7 v/v) was seeded with **M**$_3^{Seed}$ at 293 K with [**M**$_5$]/[**M**$_3^{Seed}$] = 3:1 (v/v). To 1 volume of the obtained block copolymer (**BAB**) solution were added 0.5 volume of **M**$_3$ (1.2 mM, cyclohexane/chloroform 93:7 v/v) to obtain **ABABA** copolymer. **b**, AFM height images of **ABABA** supramolecular block copolymer.

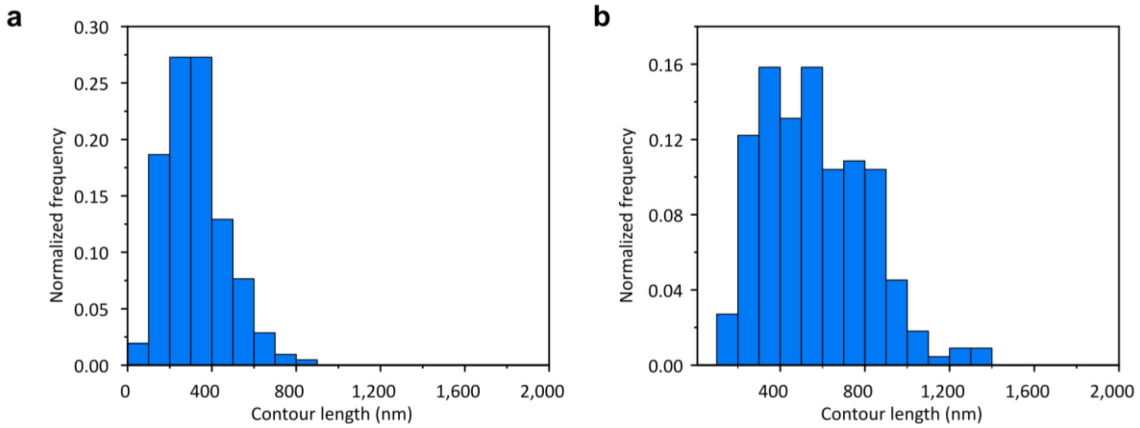

Supplementary Figure 41 | Contour length distribution obtained from AFM images of supramolecular polymers. **a M**$_5$**-M**$_3$**-M**$_5$ supramolecular block copolymer obtained with [**M**$_5$]/[**M**$_3^{Seed}$] = 3:1 (v/v). **b M**$_3$**-M**$_5$**-M**$_3$**-M**$_5$**-M**$_3$ obtained with [**M**$_5$**-M**$_3$**-M**$_5$]/[**M**$_3$] = 2:1 (v/v). The weight-average length (L_w), number-average length (L_n) and PDI (L_w/L_n) are 673 nm, 566 nm and 1.19.

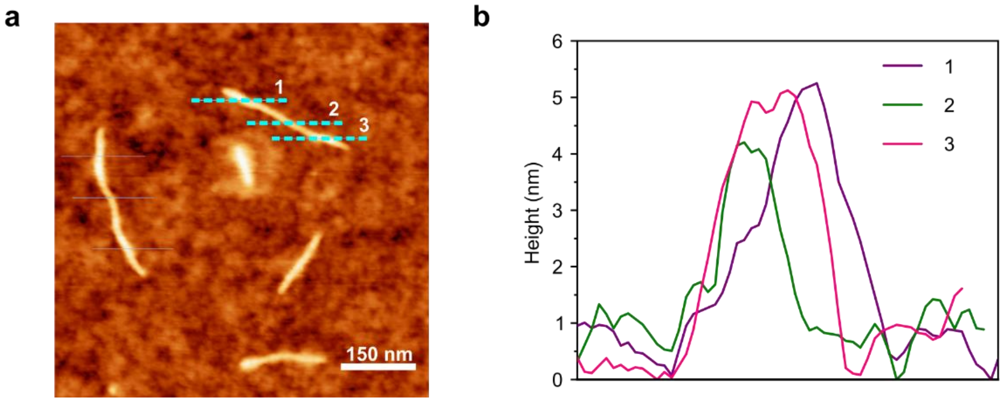

Supplementary Figure 42 | AFM height image of **M5-M3-M5** supramolecular block copolymer obtained with [**M5**]/[**M3**^{Seed}] = 3:1 (v/v) ratio spin-coated on the silicon wafer and cross-section analyses along blue dashed lines 1 – 3.

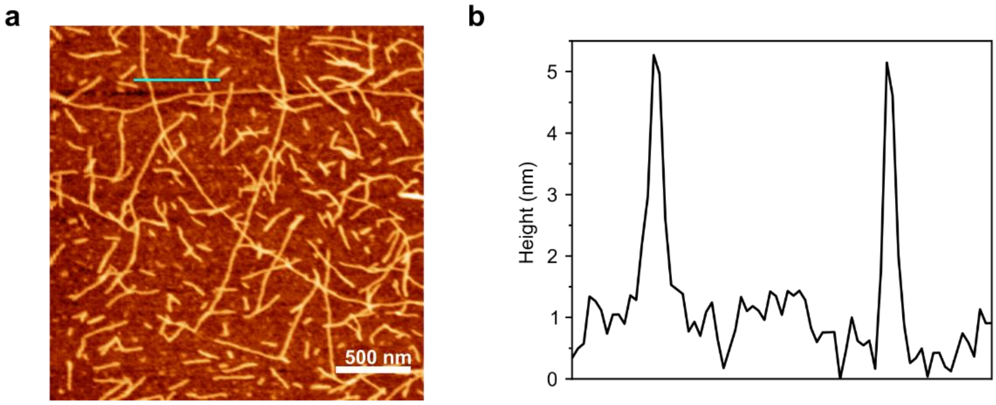

Supplementary Figure 43 | AFM height image of **M5** homopolymer obtained by sonication for 60 sec of 1.2 mM solution of **M5** (93:7 cycholexane/chloroform v/v, rt.) spin-coated on the silicon wafer and cross-section analyses along the blue line.

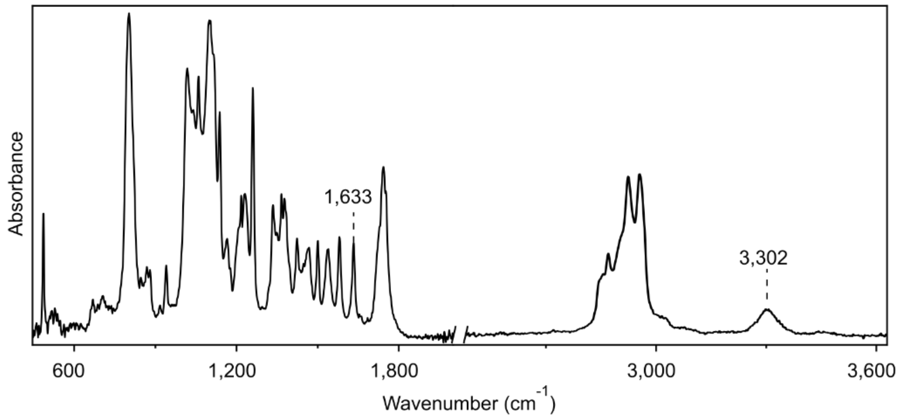

Supplementary Figure 44 | ATR-IR spectrum of $\mathbf{M_3}^{\mathbf{Polymer}}$.

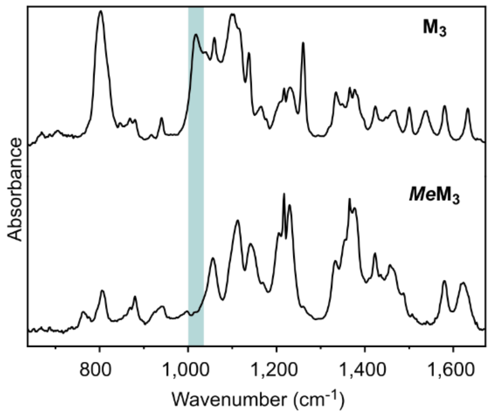

Supplementary Figure 45 | Partial ATR-IR spectra of ***Me*M3** and the supramolecular polymer of **M3**. In the case of $\mathbf{M_3}^{\mathbf{Polymer}}$ new strong C−F stretching band appears at 1018 cm^{-1} (highlighted in blue).

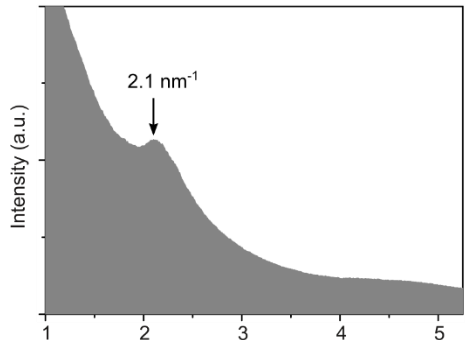

Supplementary Figure 46 | Radial-integrated intensity profile of the electron diffraction pattern shown in Fig. 5a. The diffraction ring is located at 2.1 nm^{-1}, corresponding to a lattice distance of 4.8 Å in the real space.

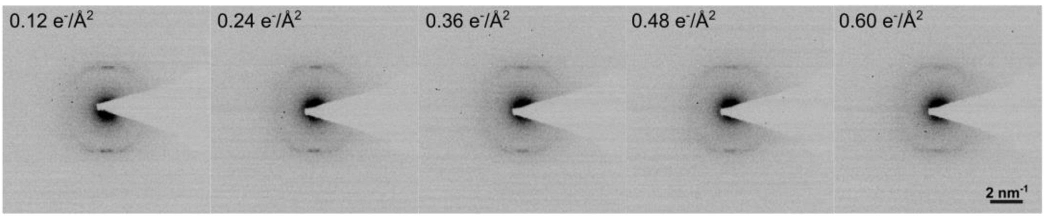

Supplementary Figure 47 | Electron dose series analysis. The selected-area electron diffraction patterns were acquired at the same position with an increasingly accumulated electron dose. With a

total electron dose of 0.12 $e^-/Å^2$, the diffraction spots remained sharp and readily visible. However, with an accumulated electron dose of 0.60 $e^-/Å^2$, only diffusive intensity could be observed, indicating substantial structural disintegration of the organic fibers.

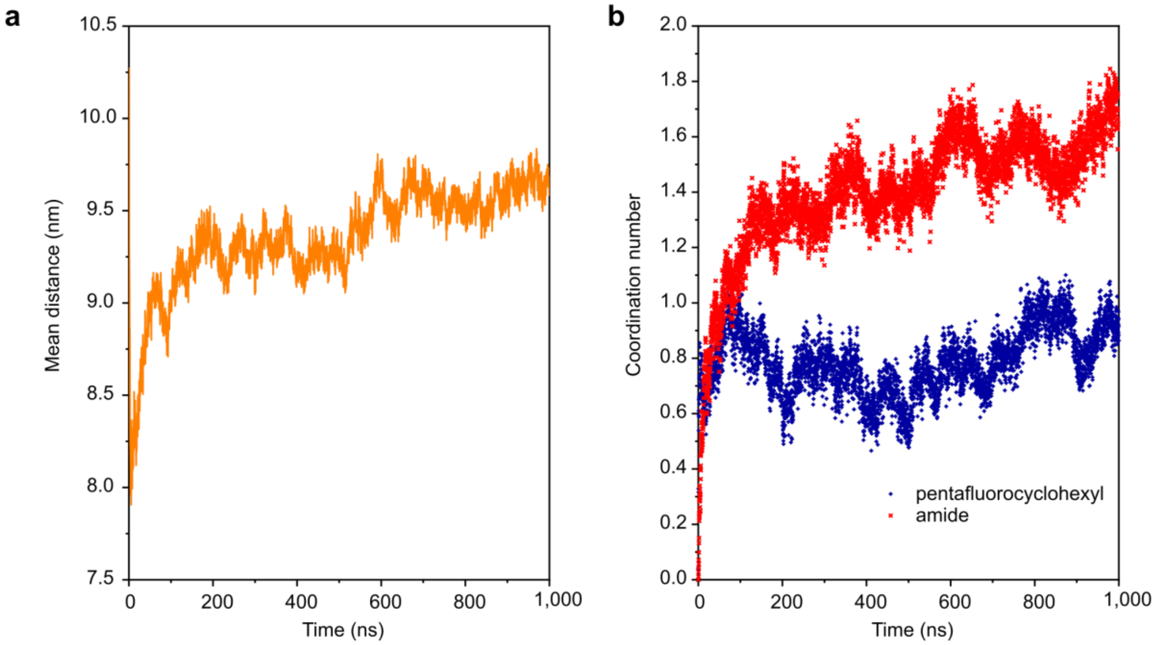

Supplementary Figure 48 | Self-assembly of 150 **M3** monomers in solution. (a) Time evolution of the mean distance between the center of the benzene and the center of the cyclohexane groups of each **M3** monomer during the MD simulation. (b) Time evolution of the coordination number between the amide (red) and pentafluorocyclohexyl (blue) groups of the **M3** monomers during self-assembly.

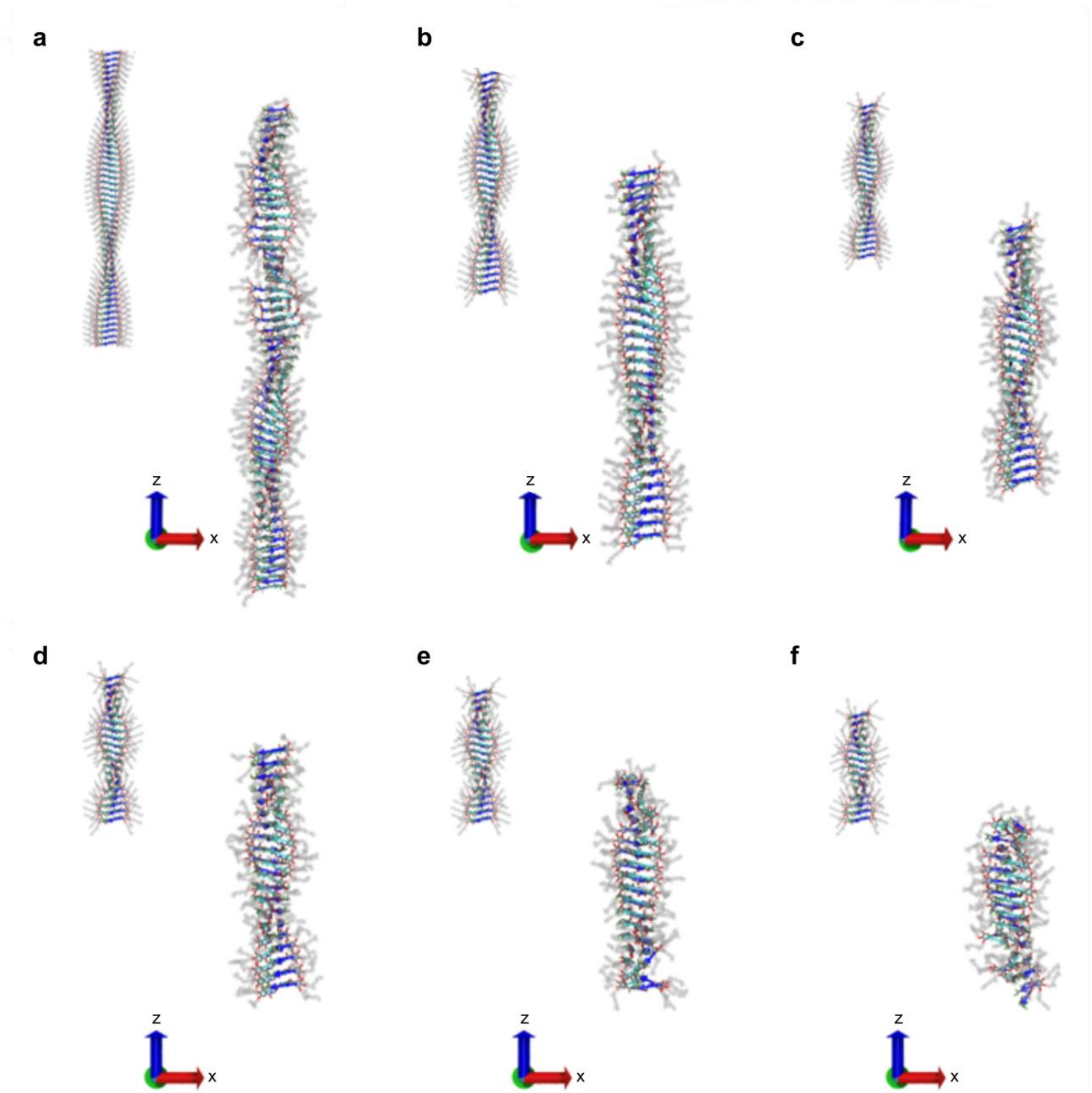

Supplementary Figure 49 | Atomistic models of preformed helical assemblies (top-left of each panel) of **M3** which are relaxed during 1μs of MD simulations (last equilibrated MD frame shown as zoomed at the bottom right of each panel). The starting configurations for the assemblies are prepared starting from the same dimeric units and changing in each system the initial torsion between them (helical pitch) along the fibers to ~24 (**a**), ~15 (**b**), ~12 (**c**), ~10 (**d**), ~9 (**e**), and ~7.5 nm (**f**). The structures (starting and equilibrated) are shown at atomistic level showing the heavy atoms (licorice representation), overlaid by blue and cyan arrows that connect the benzene group (tail) to the pentafluorocyclohexyl (head) columns of monomers. Alkyl chains are shown in transparency.

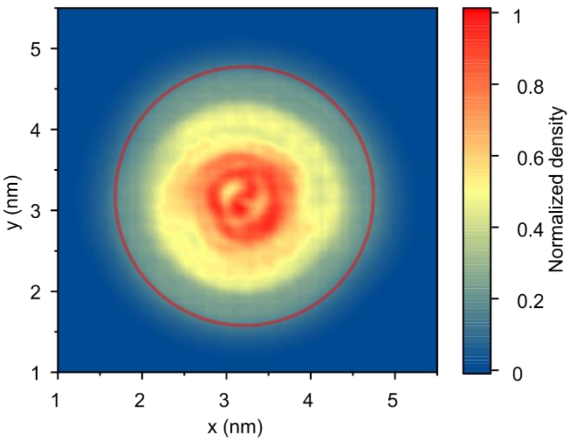

Supplementary Figure 50 | Normalized atom distribution of the average *xy* section of the $\mathbf{M_3}^{\mathbf{Polymer}}$ fiber (*z* being the main fiber axis). High densities are colored in red, while low densities are green/blue. The red circle indicates the experimental estimation of the fiber thickness.

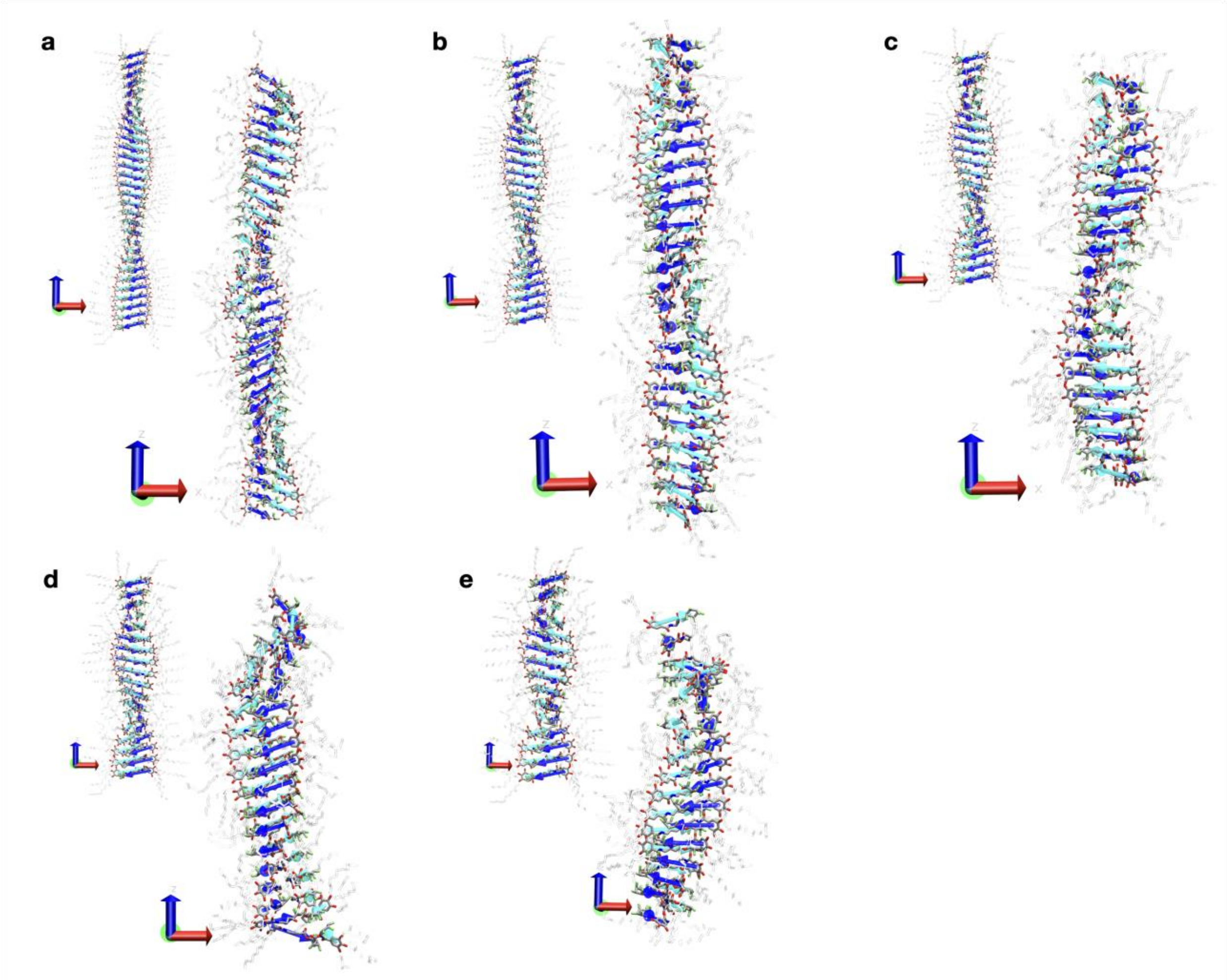

Supplementary Figure 51 | Atomistic models of preformed helical assemblies (top-left of each panel) of $\mathbf{M_5}$ which are relaxed during 1 μs of MD simulations (last equilibrated MD frame shown as zoomed at the bottom right of each panel). The starting configurations for the assemblies are prepared starting from the same dimeric units and changing in each system the initial torsion between them (helical pitch) along the fibers to ~15 (**a**), ~12 (**b**), ~10 (**c**), ~9 (**d**), and ~7.5 nm (**e**). The structures (starting and equilibrated) are shown at atomistic level showing the heavy atoms (licorice representation), overlaid by blue and cyan arrows that connect the benzene group (tail) to the pentafluorocyclohexyl (head) columns of monomers. Solvophilic chains are shown in transparency.

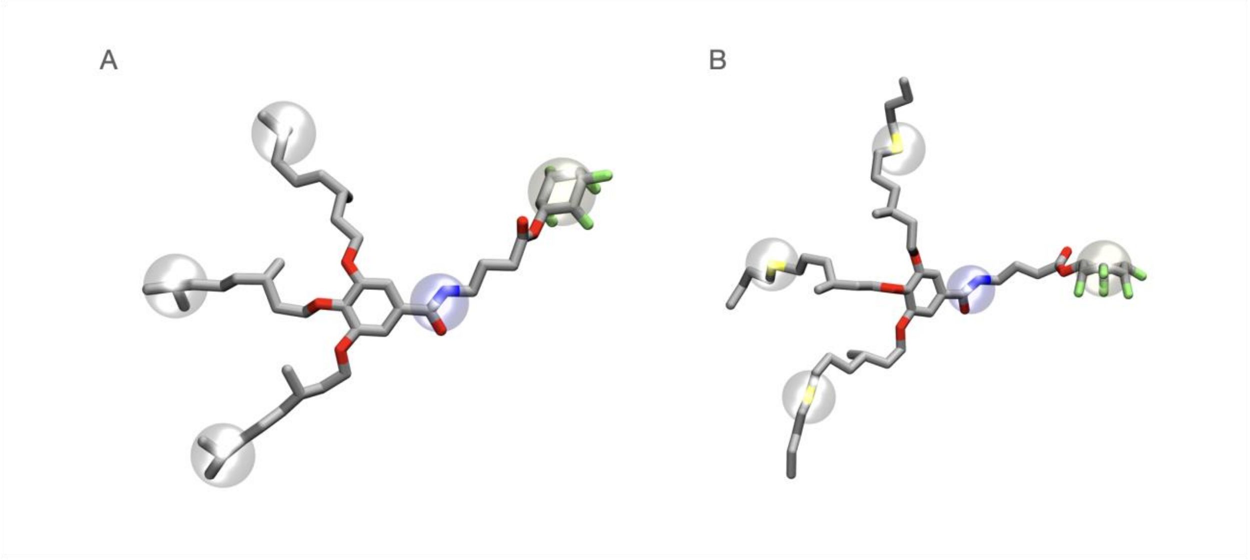

Supplementary Figure 52 | Localization of the SOAP centers (transparent spheres) on the **M$_3$** (panel A) and **M$_5$** (panel B) monomers. Colour code: green: F; red: O; blue: N; grey: C.

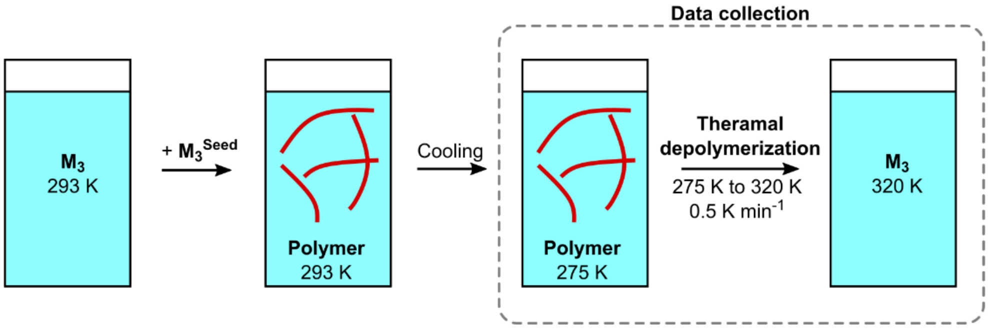

Supplementary Figure 53 | Schematic illustration of the experimental design used to investigate thermodynamic parameters of supramolecular polymerization.

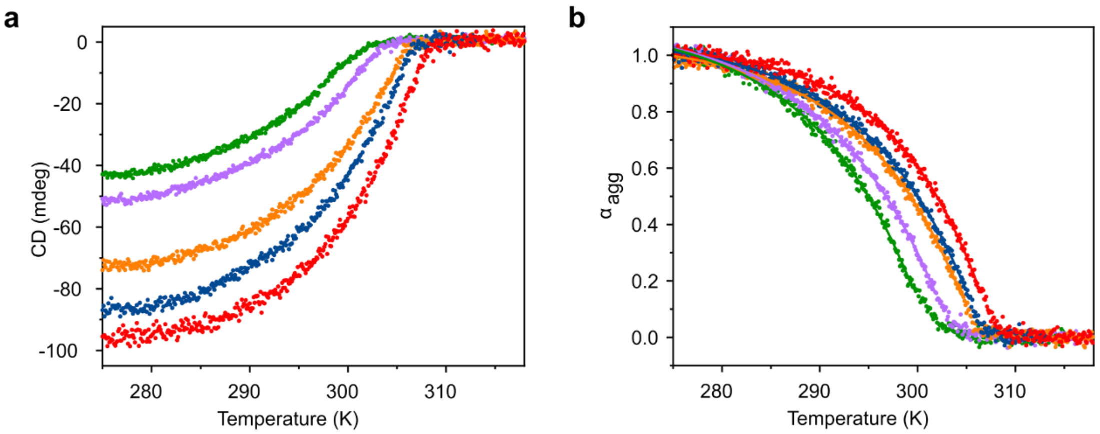

Supplementary Figure 54 | a, Changes of the CD signal (260 nm) of **M$_3^{Polymer}$** during heating at different total concentration of the **M$_3$**. Conditions: cyclohexane/chloroform 84:16 (v/v), 0.5 K min^{-1}. Color code: red, c = 1.6 mM; blue, c = 1.4 mM; yellow, c = 1.2 mM; pink, c = 1.0 mM; green, c = 0.8 mM. **b**, Temperature-dependent degree of aggregation. Solid lines represent fit according to the cooperative model.

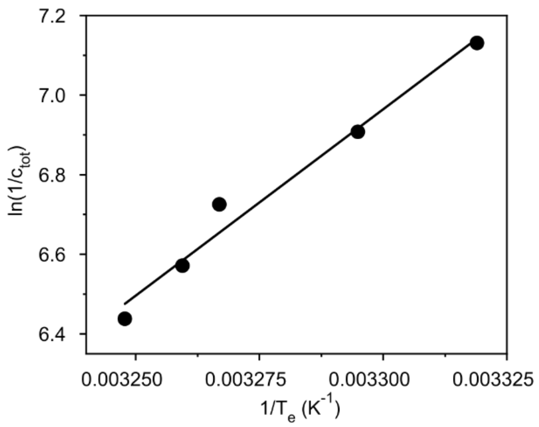

Supplementary Figure 55 | van’t Hoff plot obtained from plotting the natural logarithm of $1/c_{tot}^{-1}$ as a function of $1/T_e^{-1}$.

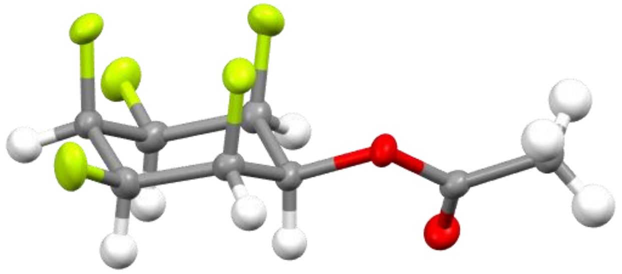

Supplementary Figure 56 | Crystal structure of all-*cis* 2,3,4,5,6-pentafluorocyclohexyl acetate. Thermal ellipsoids are shown at the 50% probability level. Single crystals all-*cis*-2,3,4,5,6-pentafluorocyclohexyl acetate (**Ref$_1$**) were obtained by slow evaporation of its solution in $CHCl_3$/acetone 9:1 (v/v). The cif-file was deposited in the Cambridge Structural Database under identifier CCDC 2010482.

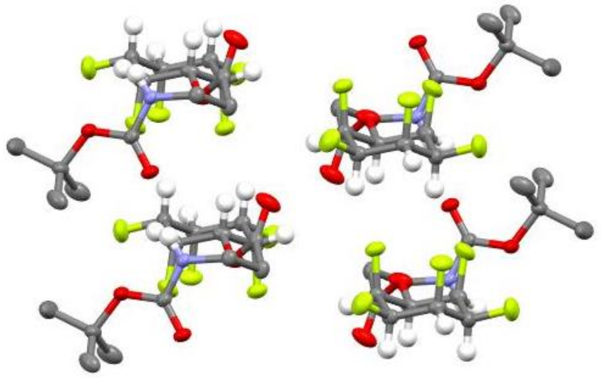

Supplementary Figure 57 | Crystal structure and packing of **2**. Thermal ellipsoids are shown at the 50% probability level. Non-cyclohexane hydrogens have been omitted for clarity. Single crystals of

compound **2** were obtained by slow evaporation of its solution in acetone. The cif-file was deposited in the Cambridge Structural Database under identifier CCDC 2071404.

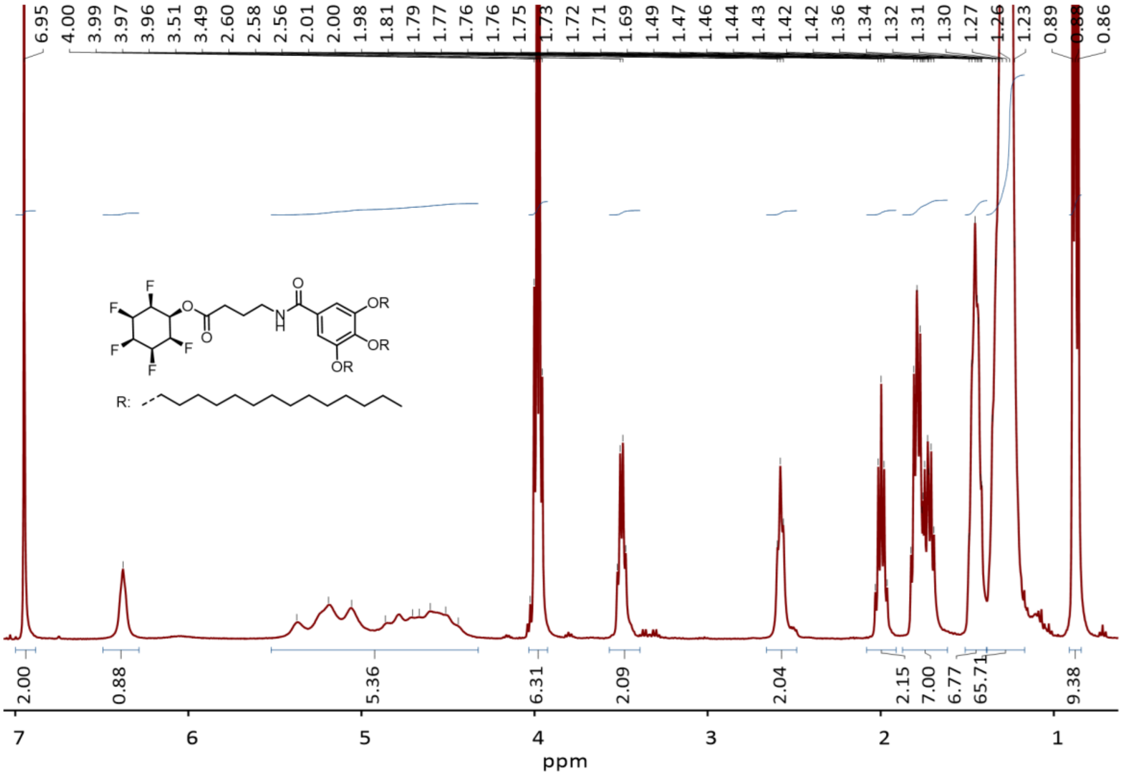

Supplementary Figure 58 | ^{1}H NMR (400 MHz) spectrum of $\mathbf{M_1}$ in $CDCl_3$ at 295 K.

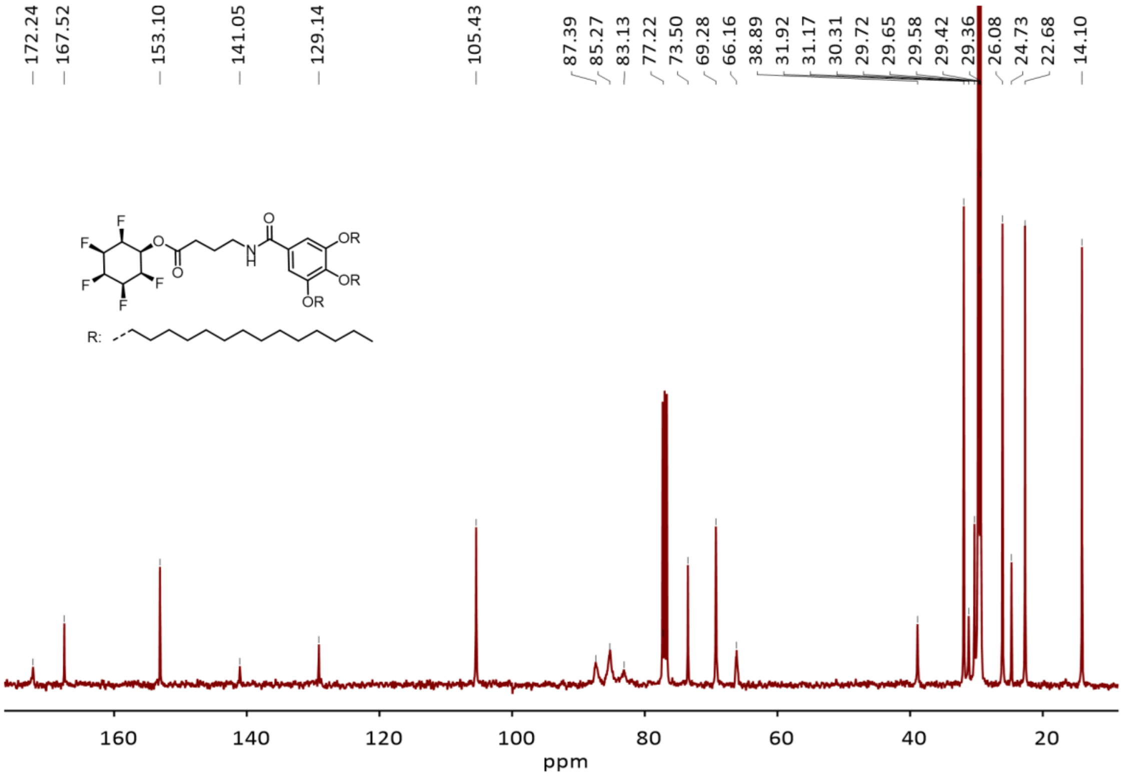

Supplementary Figure 59 | ^{13}C NMR (101 MHz) spectrum of **M_1** in $CDCl_3$ at 295 K.

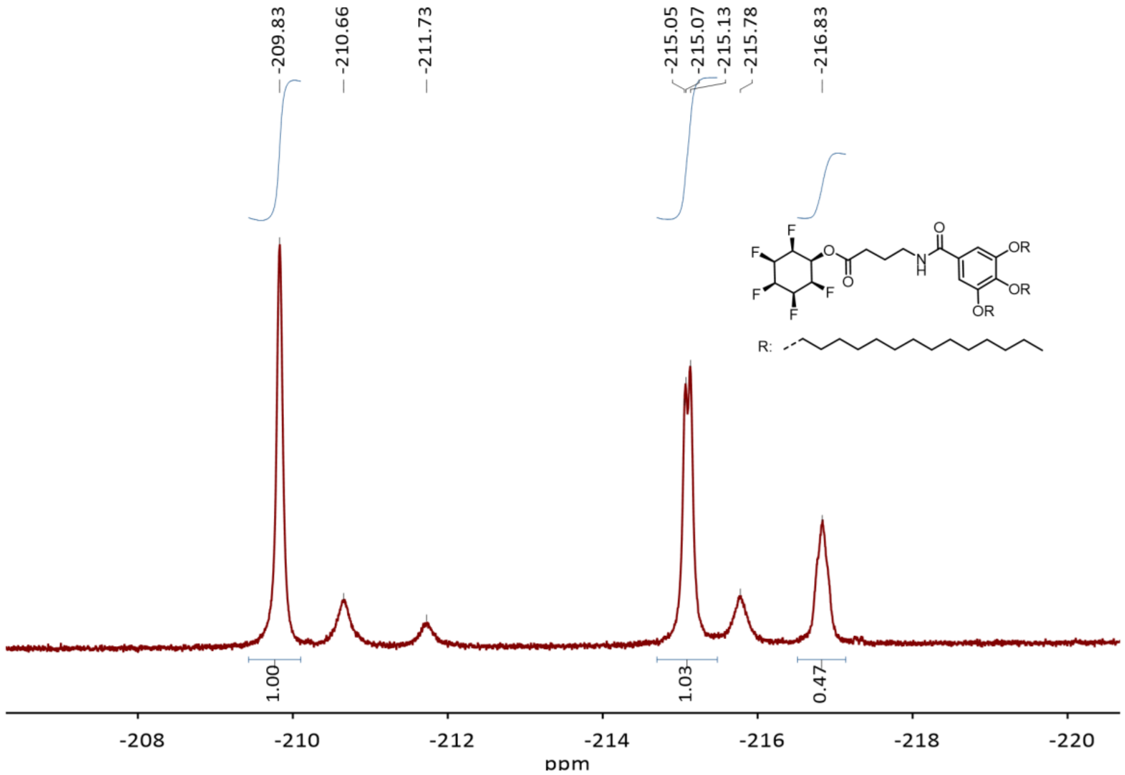

Supplementary Figure 60 | ^{19}F NMR (377 MHz) spectrum of **M_1** in $CDCl_3$ at 295 K.

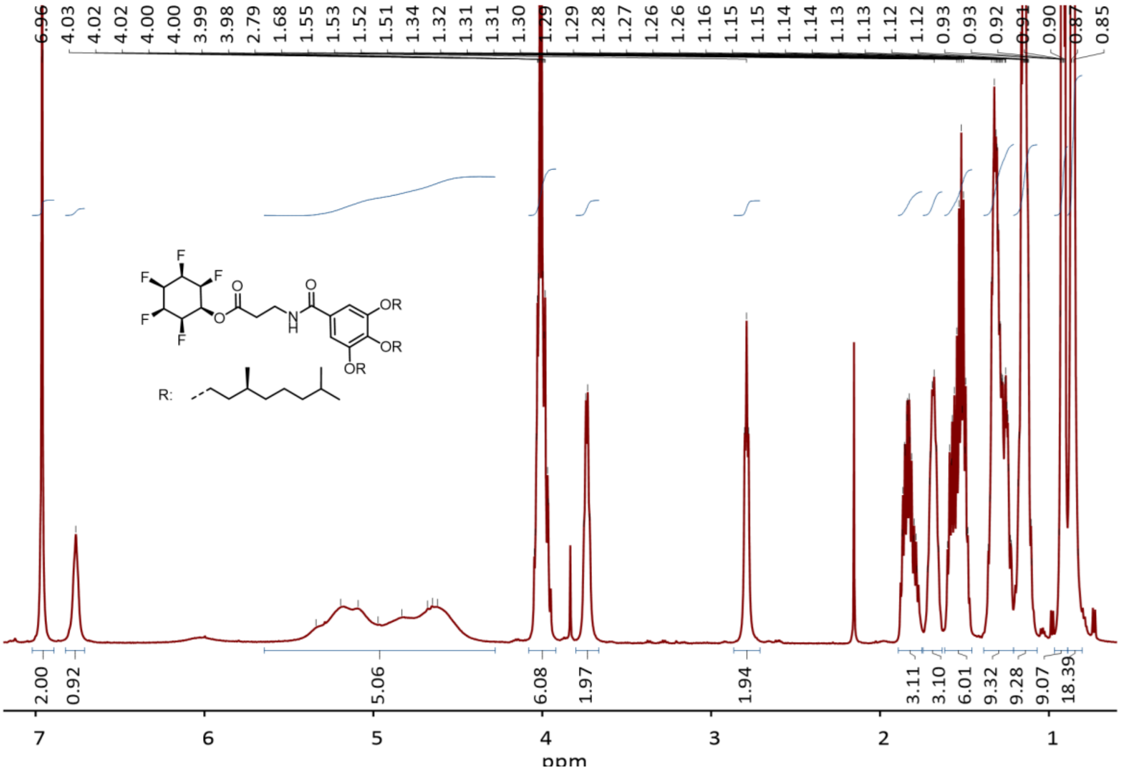

Supplementary Figure 61 | ^{1}H NMR (400 MHz) spectrum of **M_2** in $CDCl_3$ at 295 K.

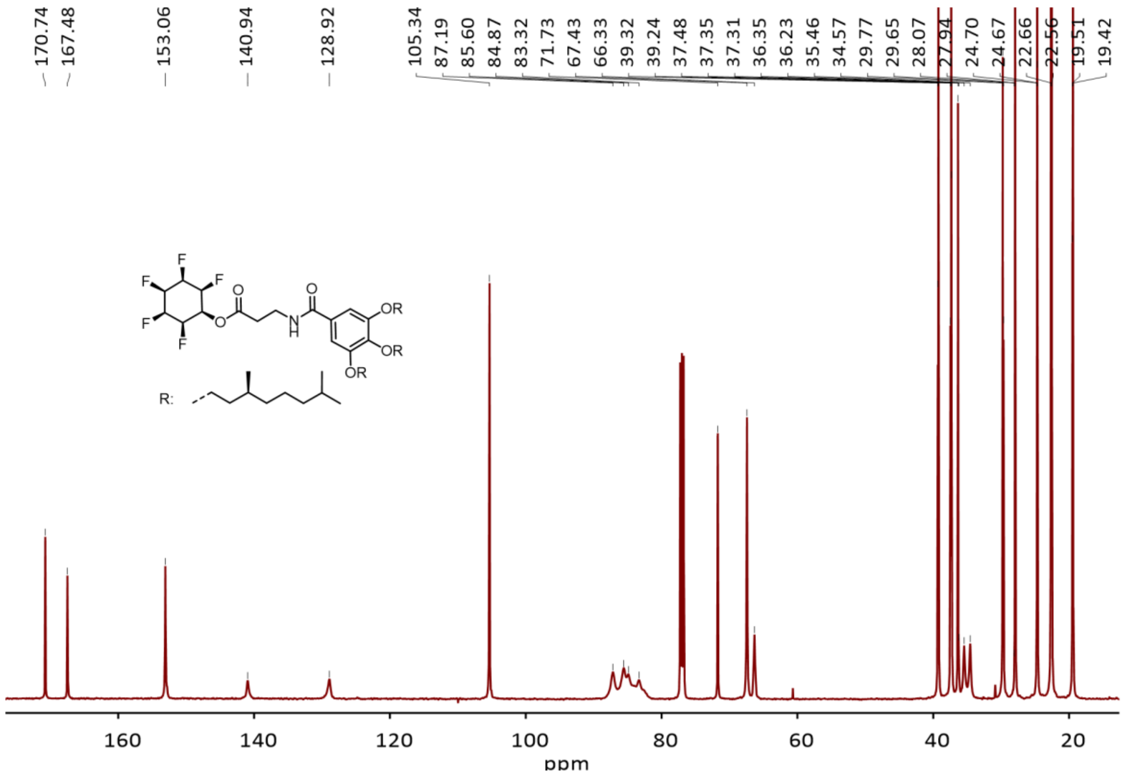

Supplementary Figure 62 | ^{13}C NMR (126 MHz) spectrum of **M_2** in $CDCl_3$ at 295 K.

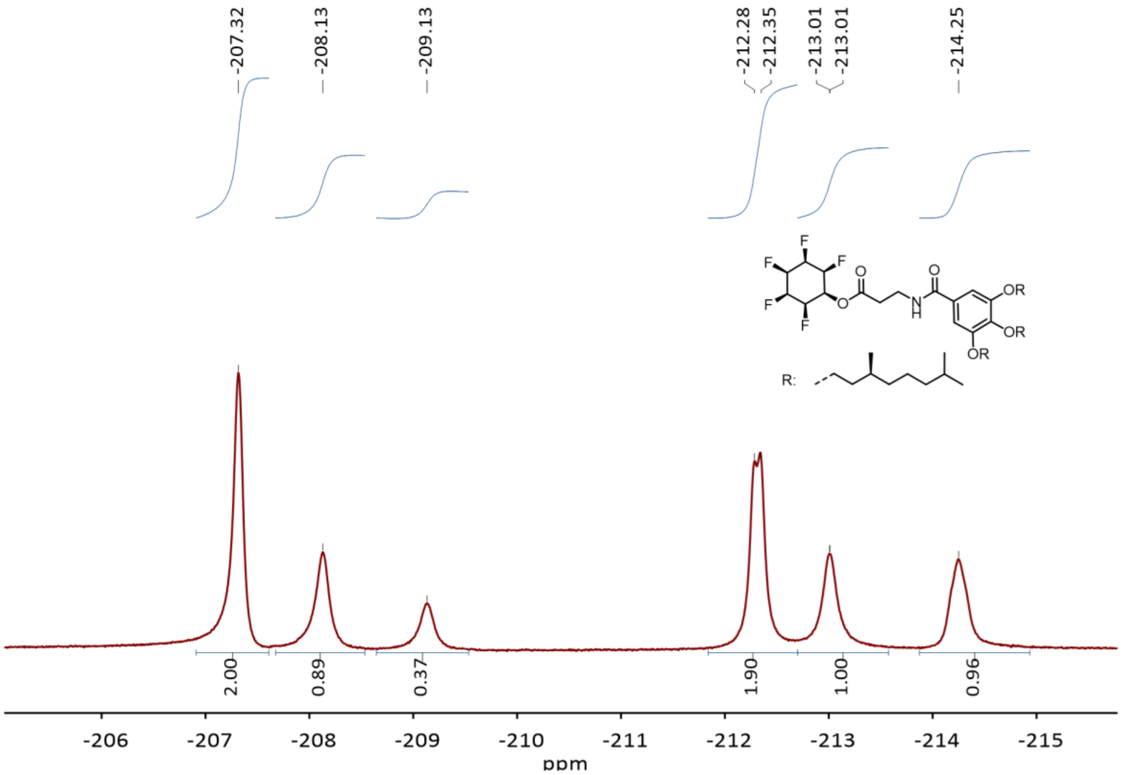

Supplementary Figure 63 | ^{19}F NMR (377 MHz) spectrum of **M_2** in $CDCl_3$ at 295 K.

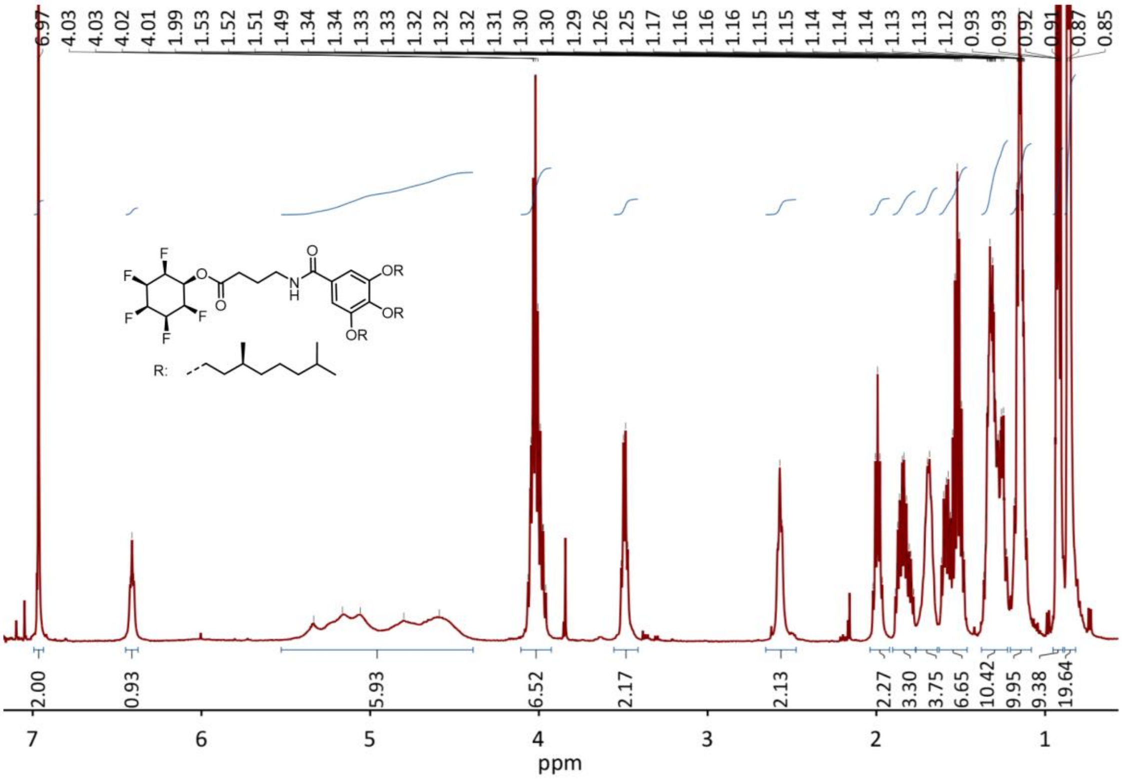

Supplementary Figure 64 | ^{1}H NMR (500 MHz) spectrum of **M$_3$** in $CDCl_3$ at 295 K.

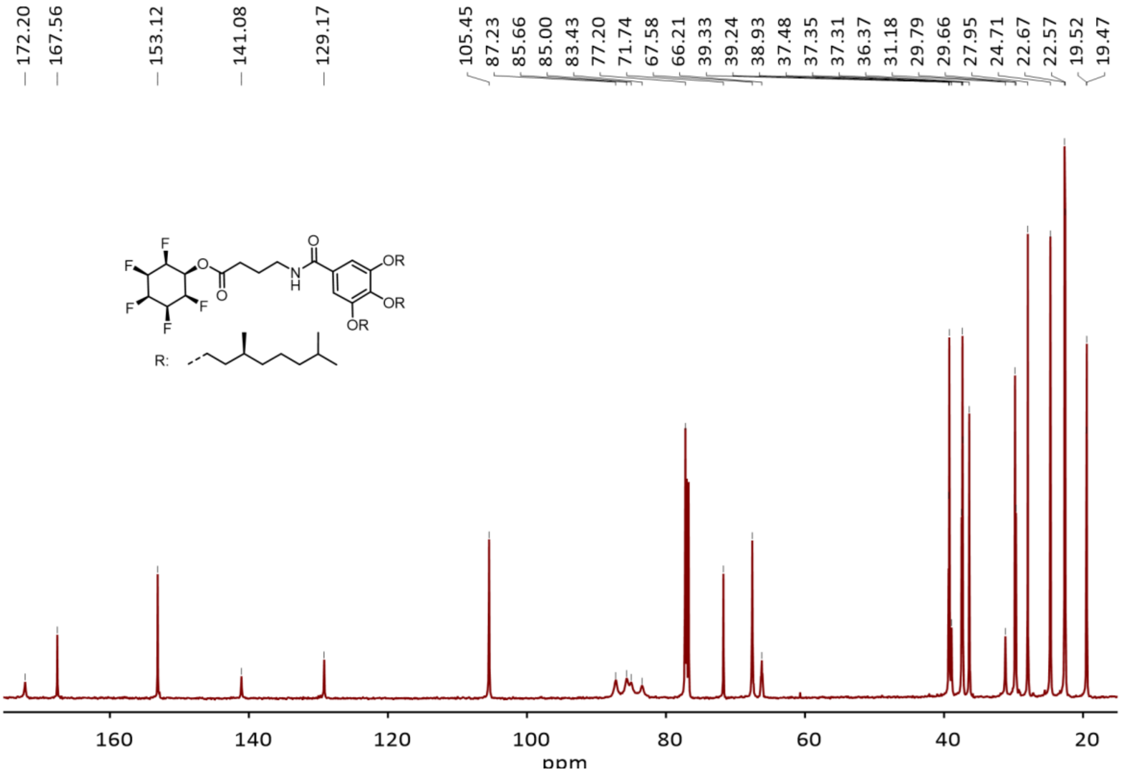

Supplementary Figure 65 |.^{13}C NMR (126 MHz) spectrum of **M$_3$** in $CDCl_3$ at 295 K.

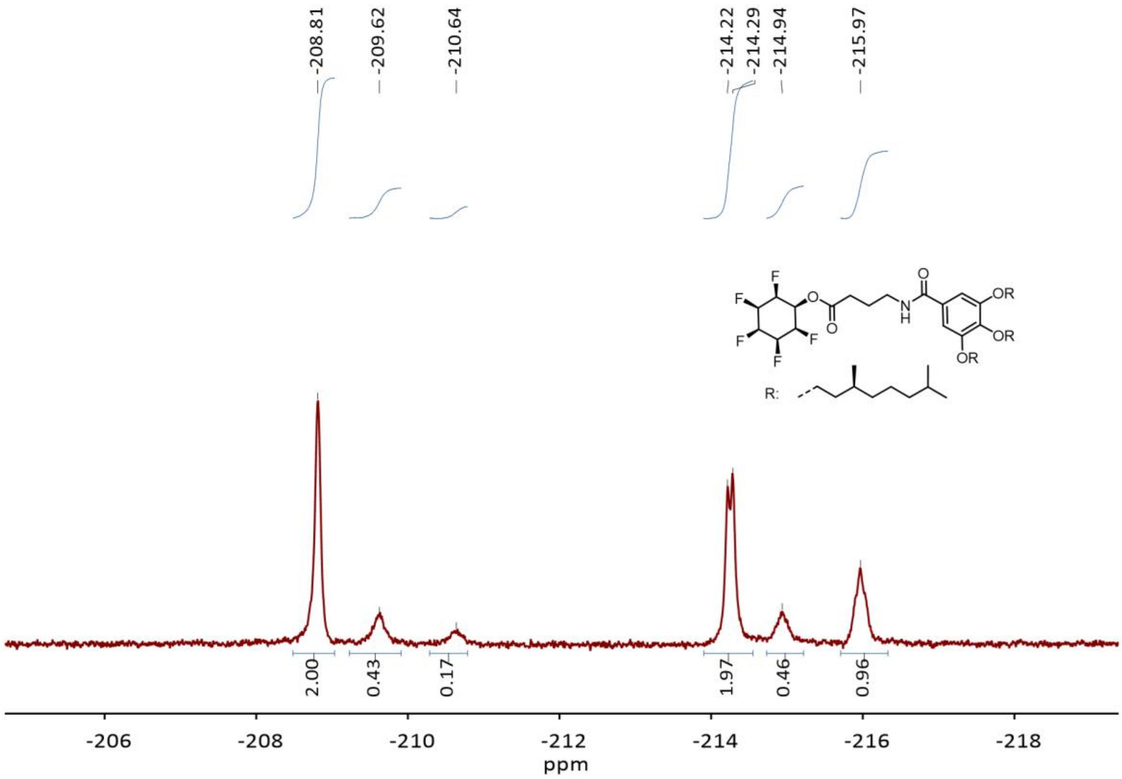

Supplementary Figure 66 | ^{19}F NMR (377 MHz) spectrum of **M_3** in $CDCl_3$ at 295 K.

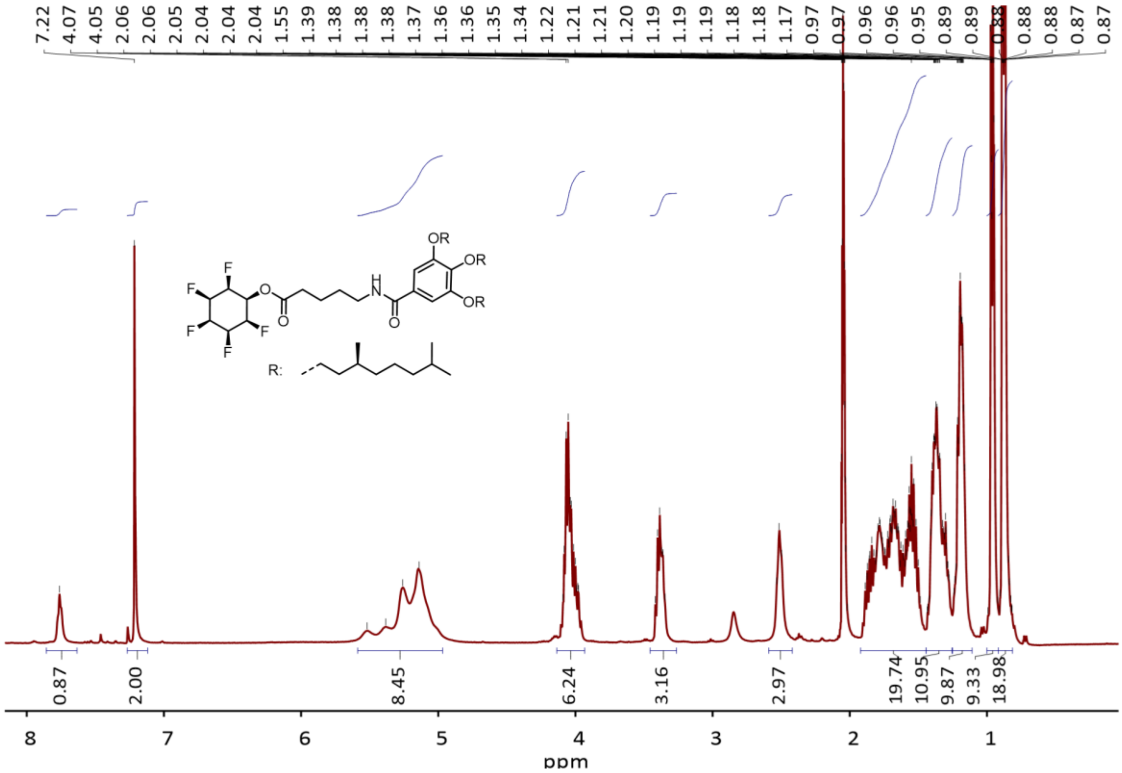

Supplementary Figure 67 | ^{1}H NMR (400 MHz) spectrum of **M_4** in acetone-d_6 at 295 K.

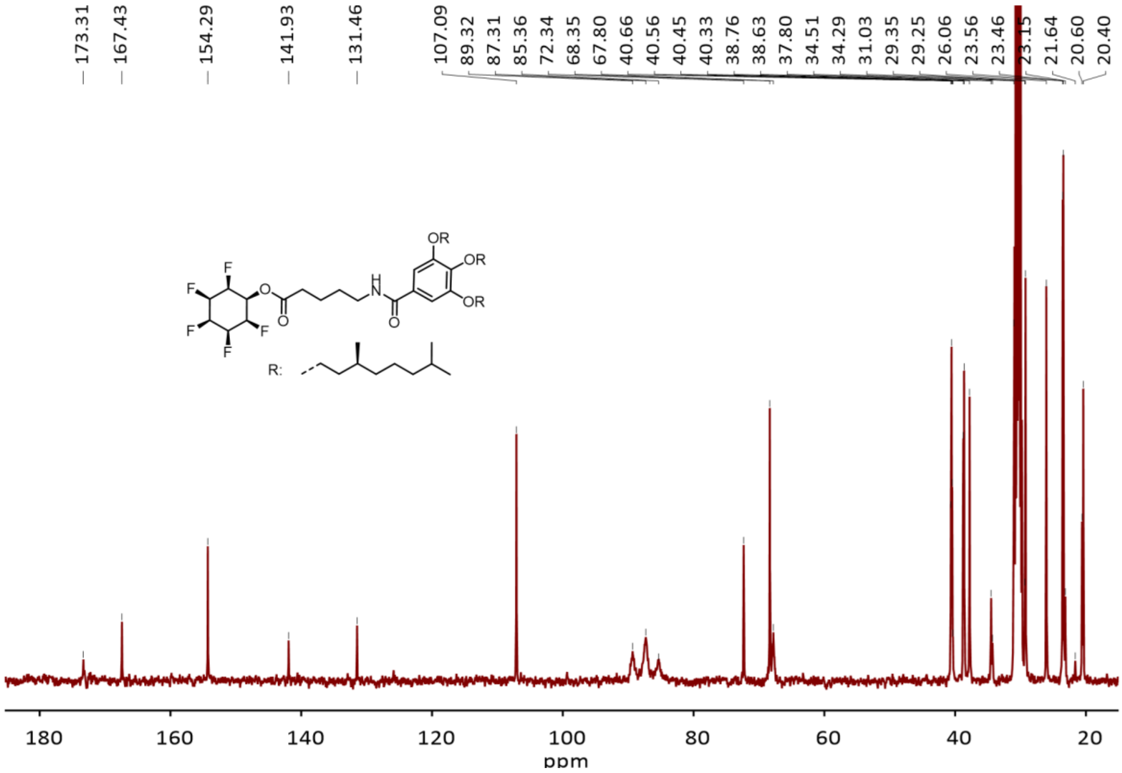

Supplementary Figure 68 | ^{13}C NMR (101 MHz) spectrum of **M**$_{4}$ in acetone-d_6 at 295 K.

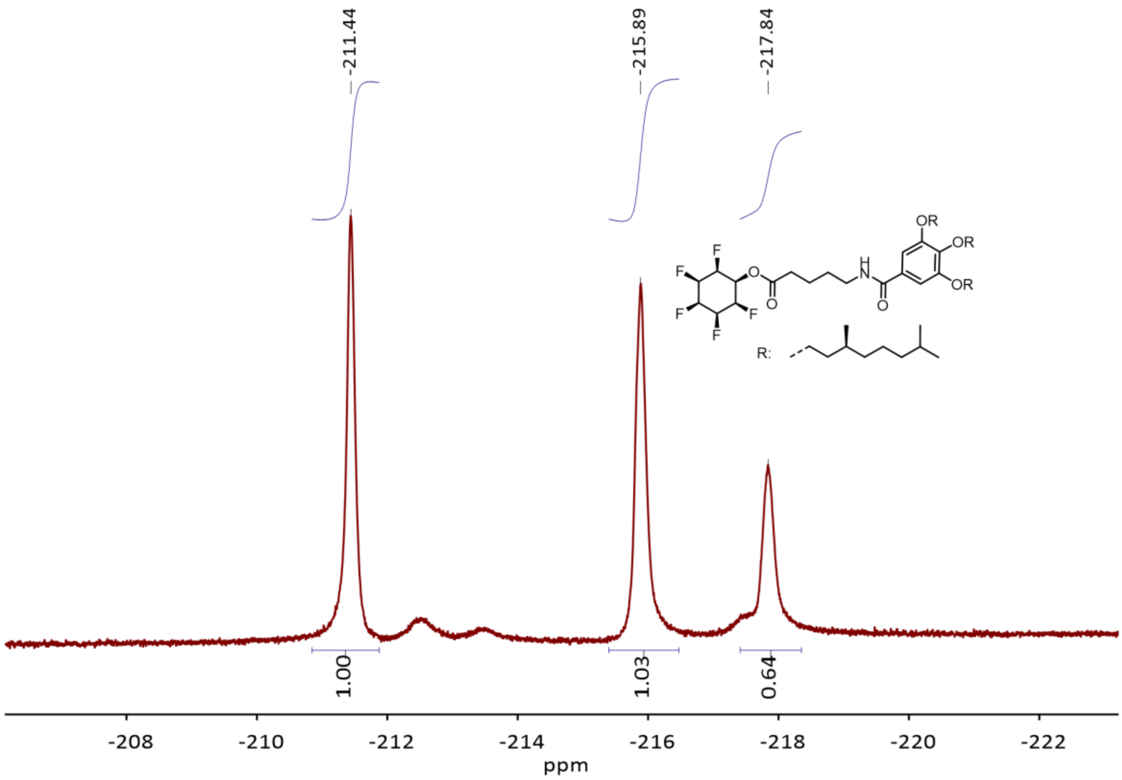

Supplementary Figure 69 | ^{19}F NMR (377 MHz) spectrum of **M**$_{4}$ in acetone-d_6 at 295 K.

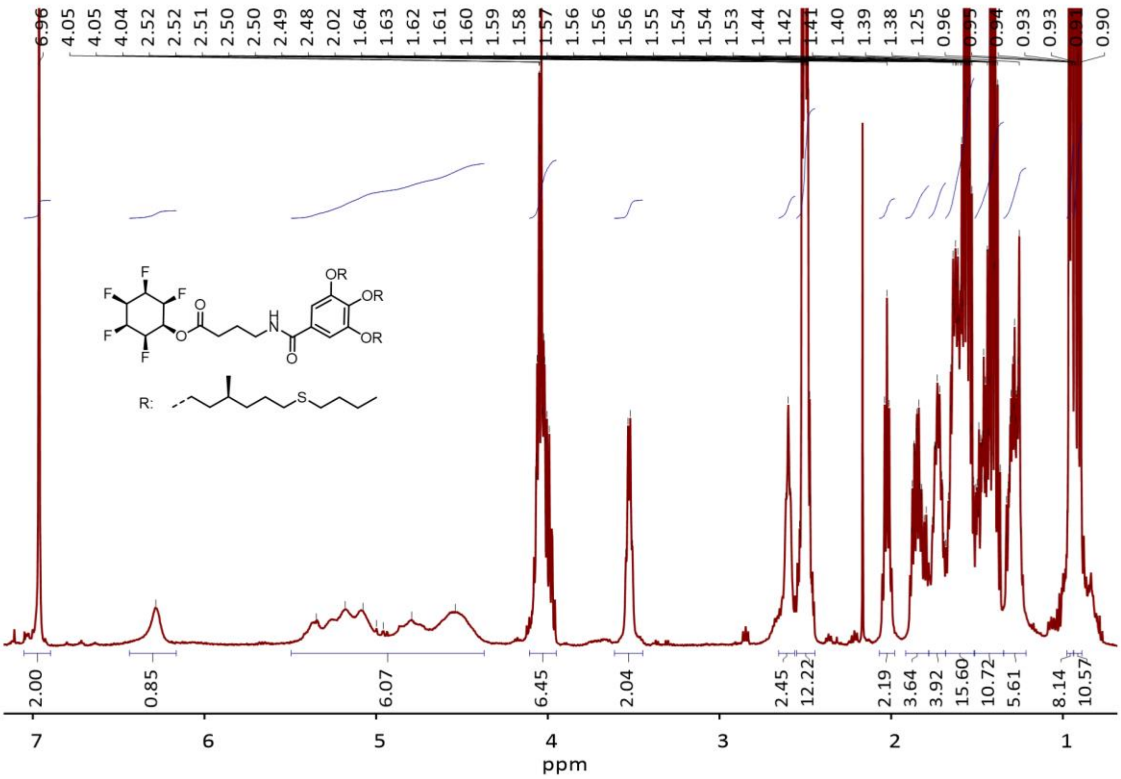

Supplementary Figure 70 | ^{1}H NMR (500 MHz) spectrum of **M5** in $CDCl_3$ at 295 K.

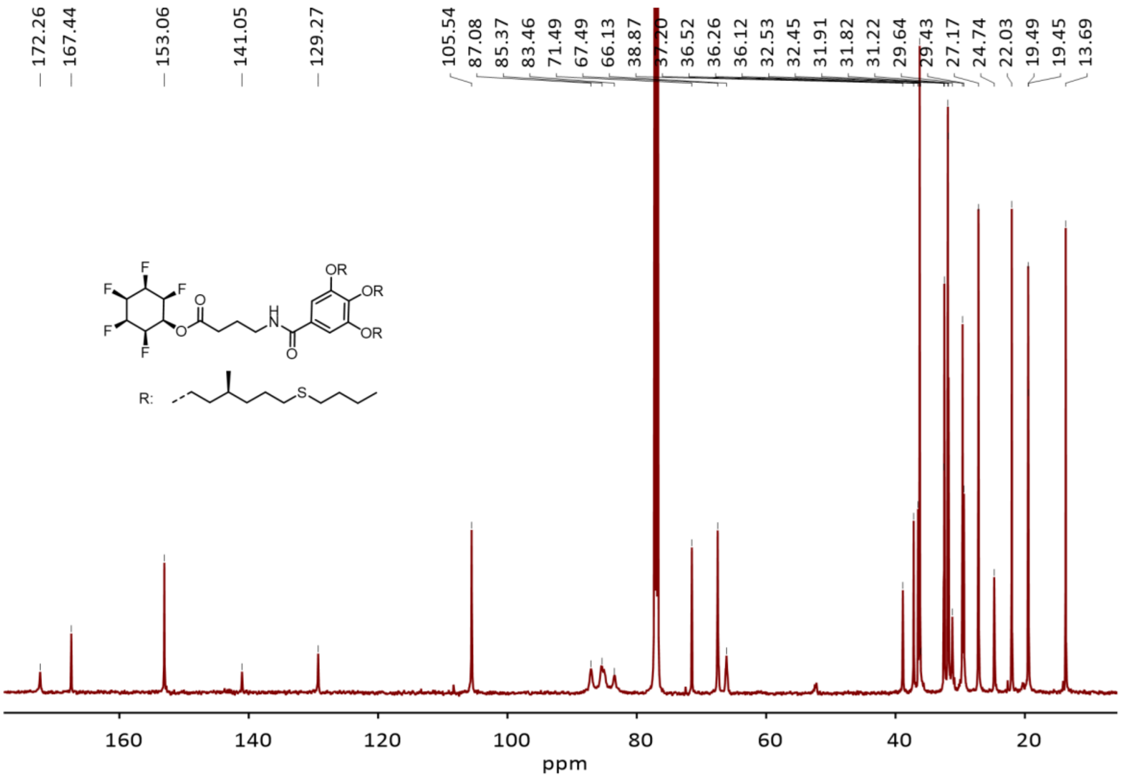

Supplementary Figure 71 | ^{13}C NMR (126 MHz) spectrum of **M5** in $CDCl_3$ at 295 K.

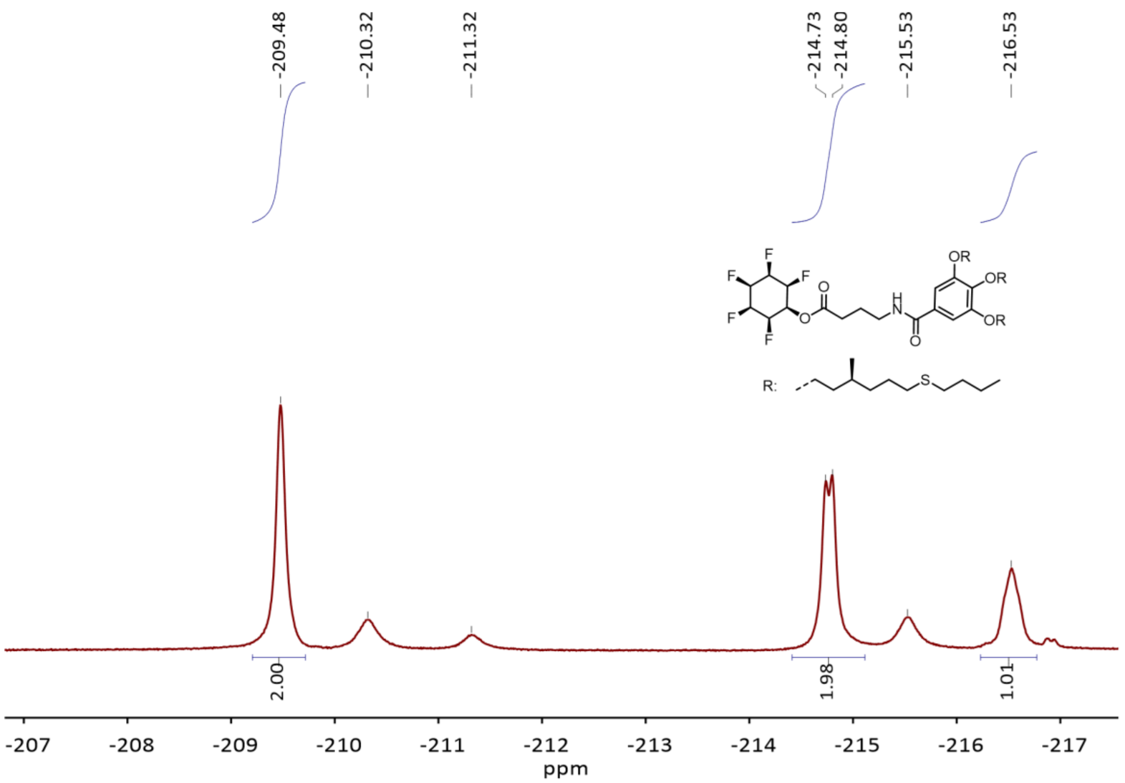

Supplementary Figure 72 | ^{19}F NMR (377 MHz) spectrum of **M$_5$** in $CDCl_3$ at 295 K.

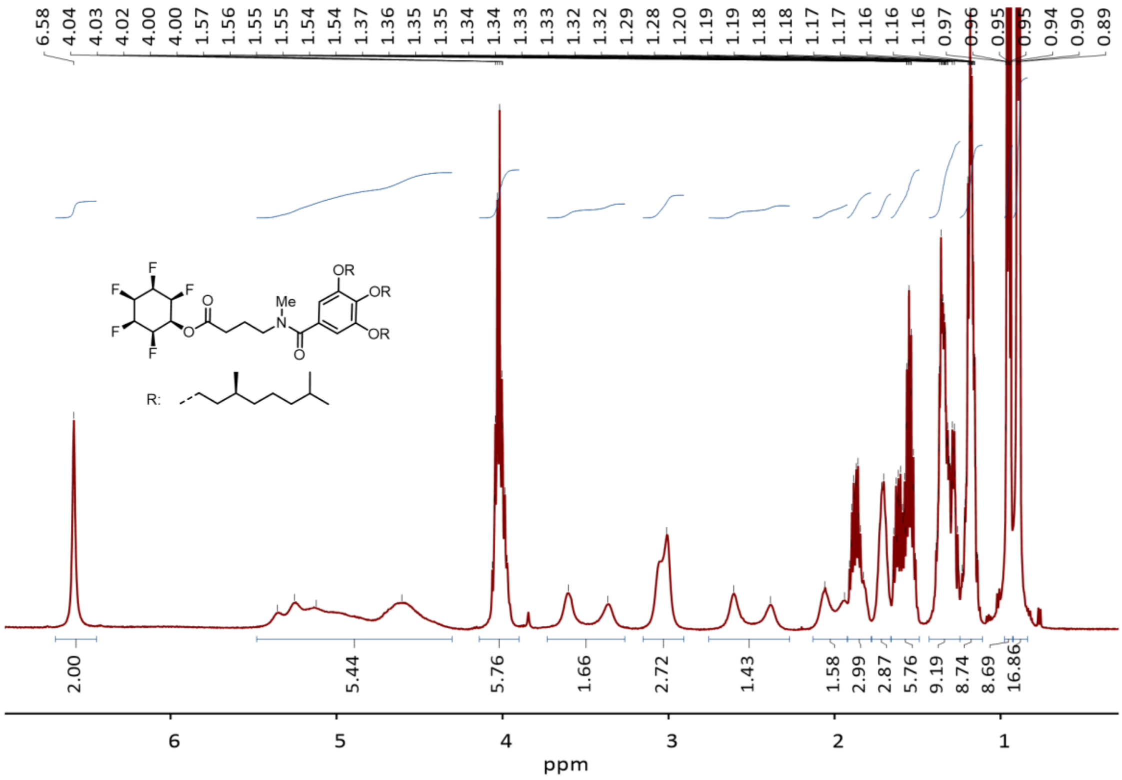

Supplementary Figure 73 | ^{1}H NMR (500 MHz) spectrum of ***Me*M$_3$** in $CDCl_3$ at 295 K.

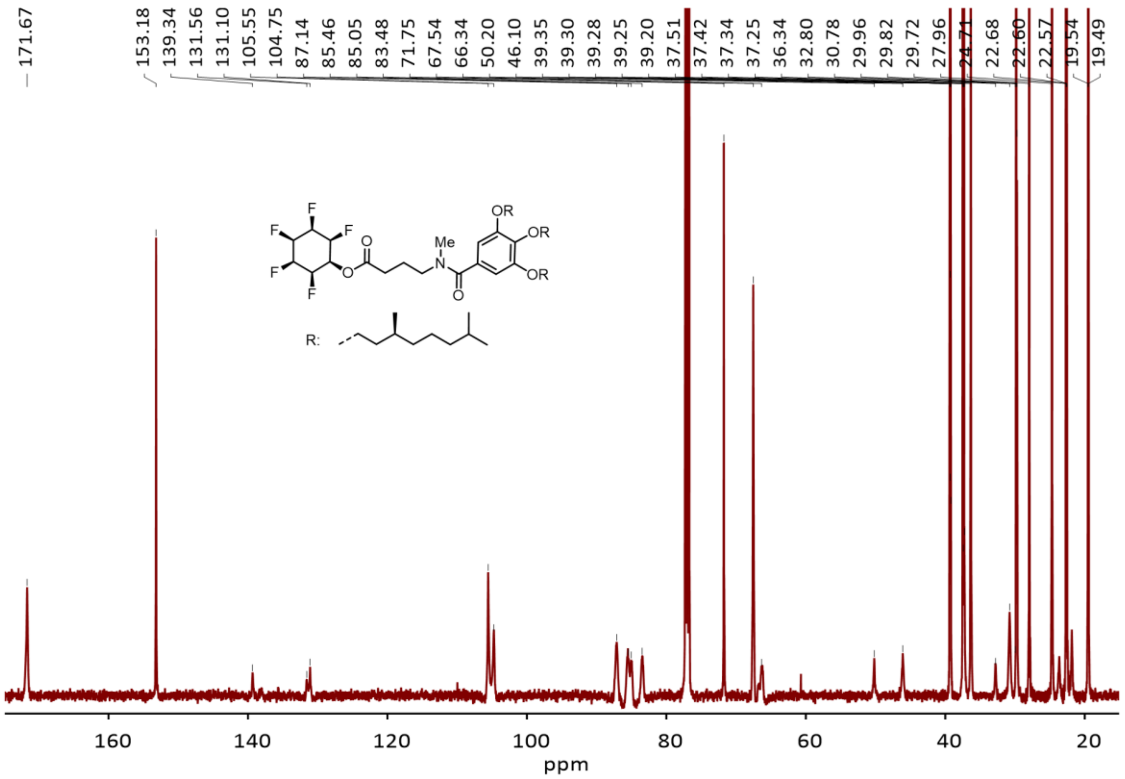

Supplementary Figure 74 |.^{13}C NMR (126 MHz) spectrum of ***Me*M3** in $CDCl_3$ at 295 K.

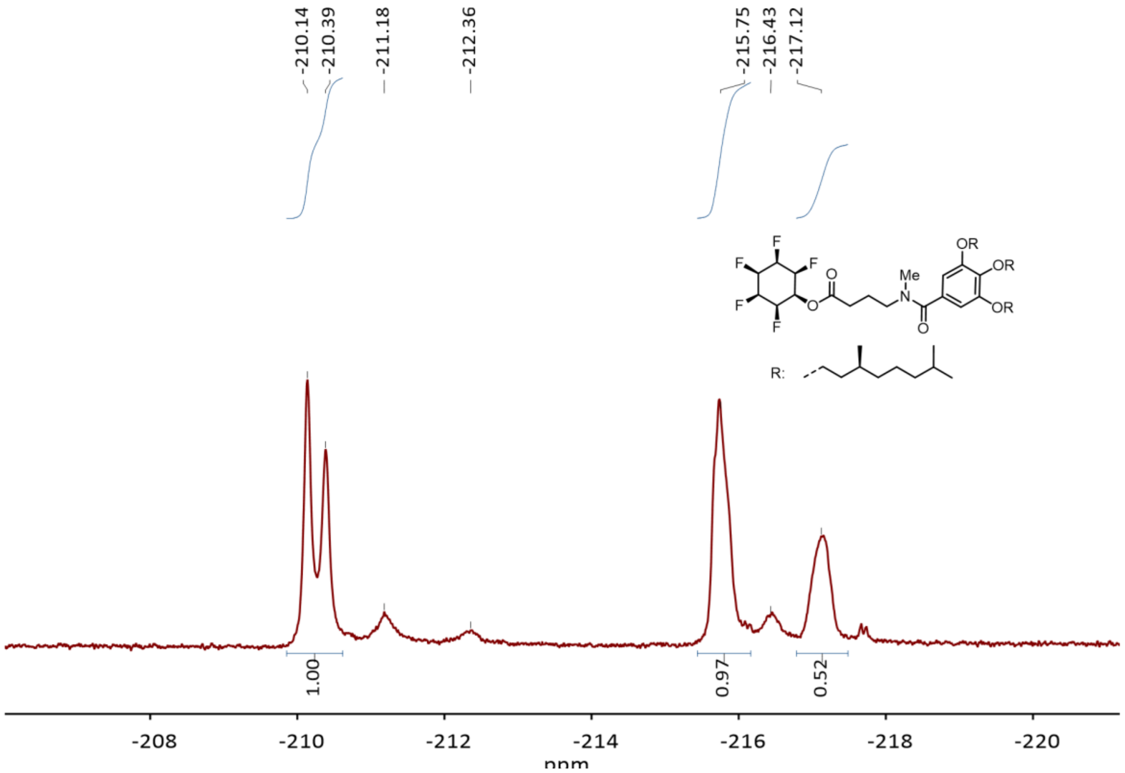

Supplementary Figure 75 |.^{19}F NMR (377 MHz) spectrum of ***Me*M3** in $CDCl_3$ at 295 K.

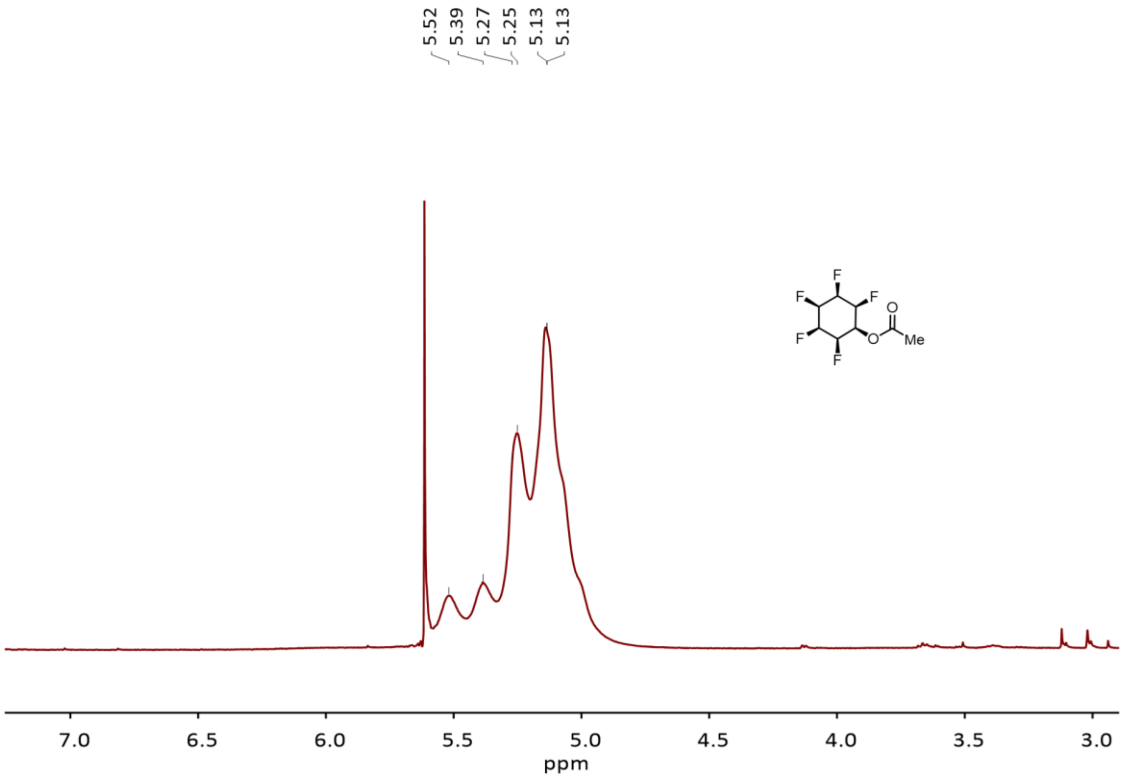

Supplementary Figure 76 | ^{1}H NMR (400 MHz) spectrum of **Ref$_{1}$** in acetone-d_6 at 295 K.

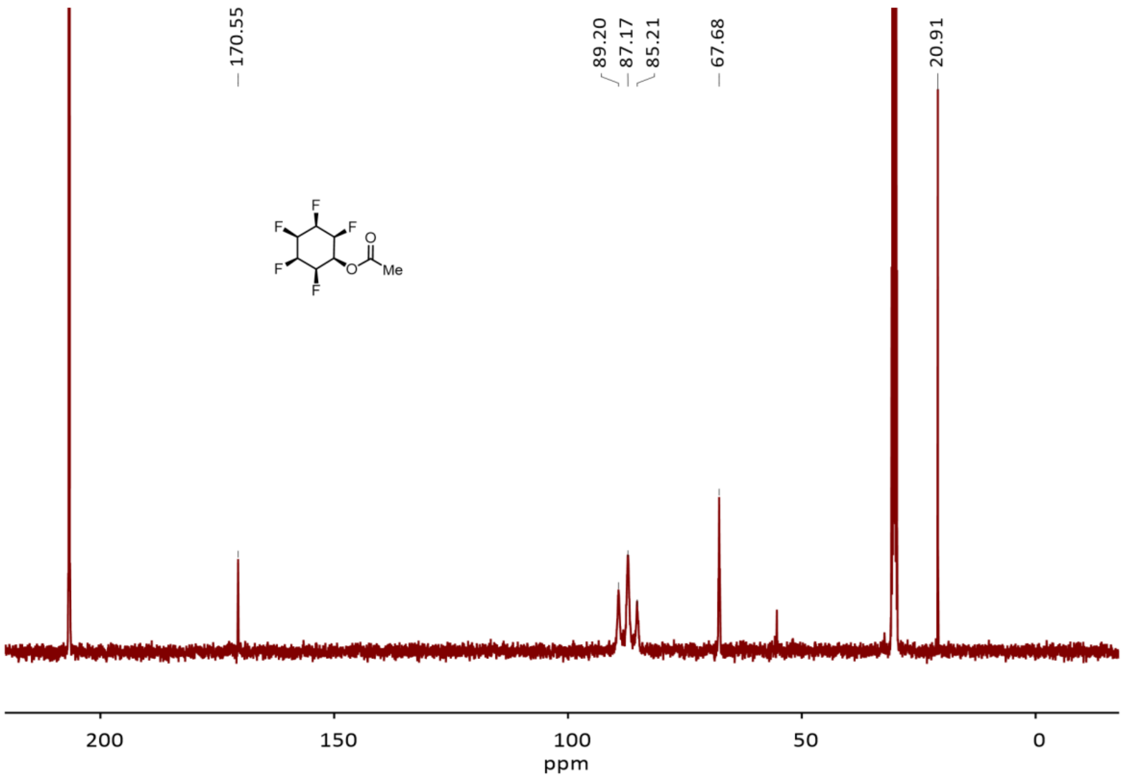

Supplementary Figure 77 | ^{13}C NMR (101 MHz) spectrum of **Ref$_{1}$** in acetone-d_6 at 295 K.

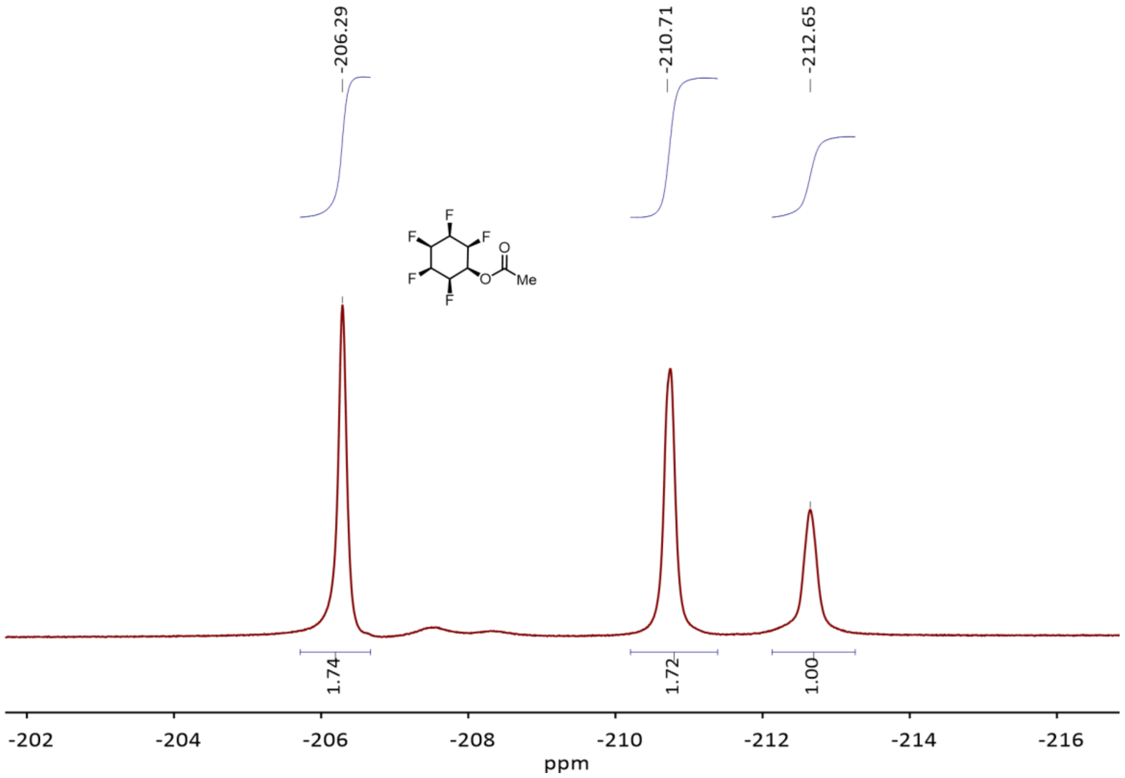

Supplementary Figure 78 | ^{19}F NMR (377 MHz) spectrum of **Ref$_1$** in acetone-d_6 at 295 K.

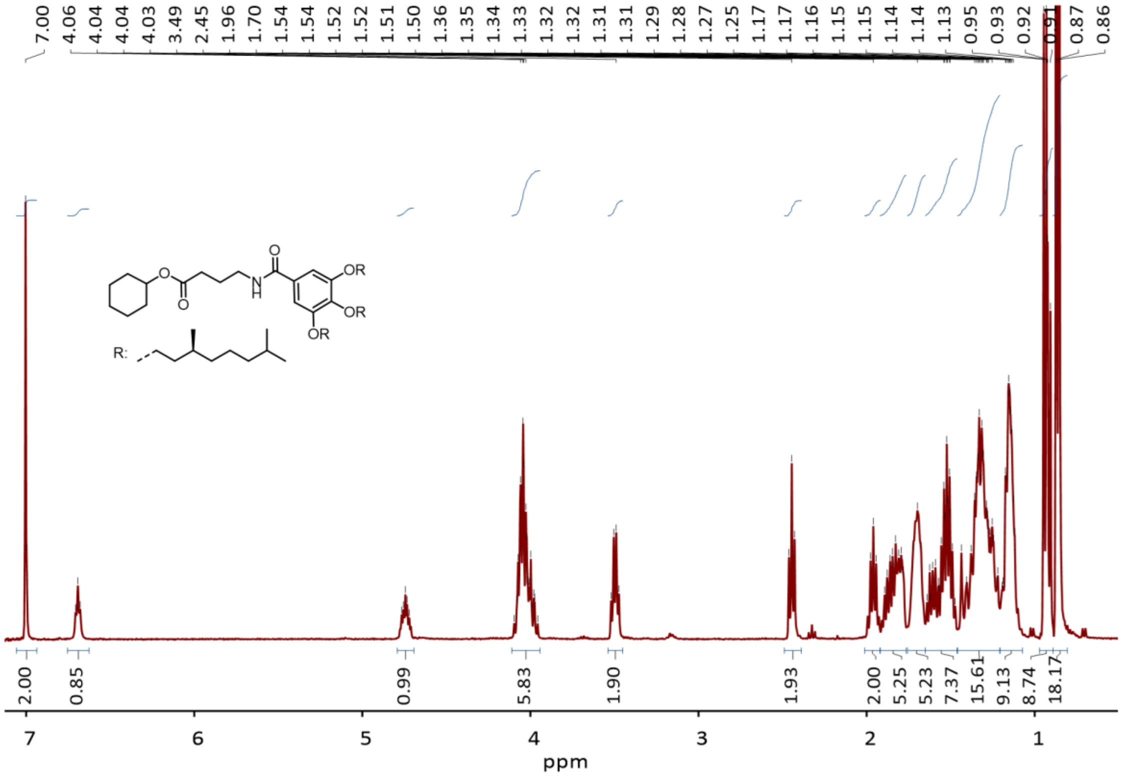

Supplementary Figure 79 | ^{1}H NMR (400 MHz) spectrum of **Ref$_2$** in $CDCl_3$ at 295 K.

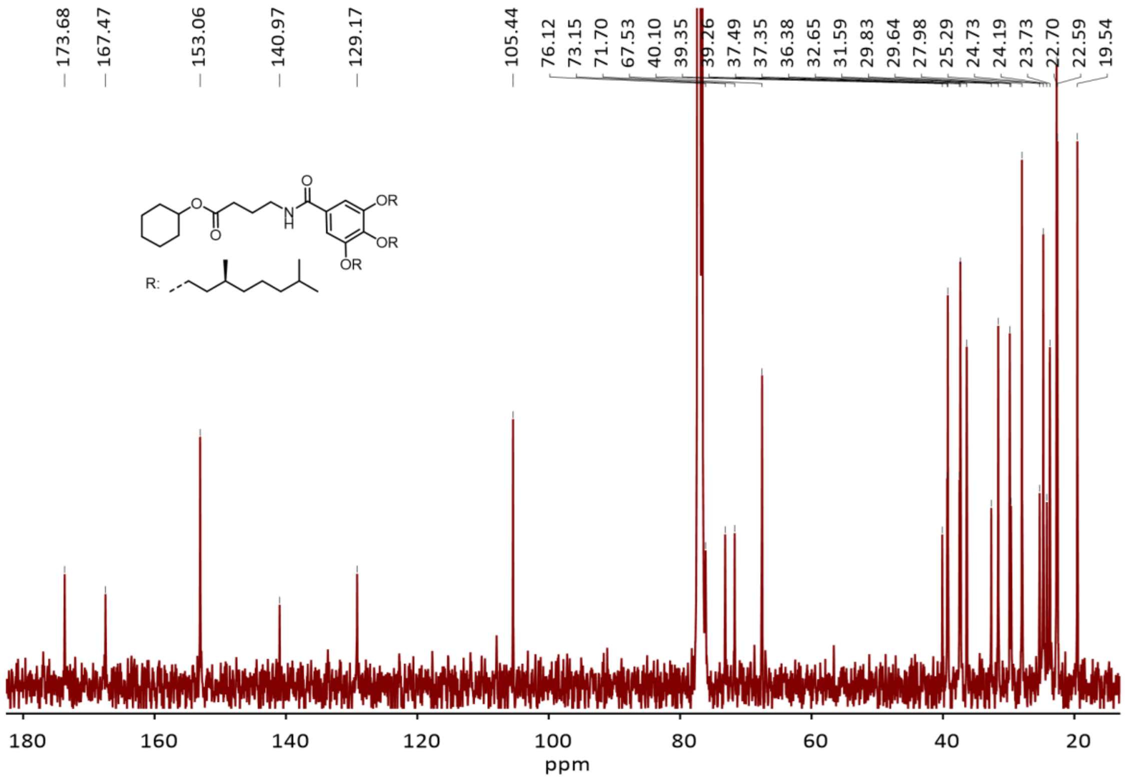

Supplementary Figure 80 | ^{13}C NMR (101 MHz) spectrum of **Ref$_2$** in $CDCl_3$ at 295 K.

Supplementary Tables

Supplementary Table 1 | Weight-averaged length (L_w), number-averaged length (L_n) and PDI (L_w/L_n) obtained by measuring lengths of n separate unbundled fibers obtained with different [$\mathbf{M_3}$]/[$\mathbf{M_3^{Seed}}$] ratios.

[$\mathbf{M_3}$]/[$\mathbf{M_3^{Seed}}$] (v/v)	n	L_w, (nm)	L_n, (nm)	PDI (L_w/L_n)
$\mathbf{M_3^{Seed}}$	216	83	70	1.19
3/1	213	317	282	1.12
5/1	203	499	418	1.19
7/1	148	552	664	1.20
10/1	201	956	809	1.18

Supplementary Table 2 | Thermodynamic parameters of polymerization obtained by fitting the temperature dependent degree of aggregation (α_{agg}) according to the cooperative model.

Conc. (M)	α_{SAT}	h_e (kJ mol^{-1})	T_e (K)
0.8×10^{-3}	1.12	-70.6	301.3
1.0×10^{-3}	1.12	-67.0	303.5
1.2×10^{-3}	1.05	-74.0	306.1
1.4×10^{-3}	1.07	-73.5	306.8
1.6×10^{-3}	1.04	-86.2	307.9

Supplementary Table 3 | Thermodynamic parameters obtained by van`t Hoff analyses.

$\Delta H°$ (kJ mol^{-1})	$\Delta S°$ (J mol^{-1} K^{-1})	ΔG^{293} (kJ mol^{-1})
-77.9	-199	-19.6

Supplementary Table 4 | Crystal data and structure refinement for OSH19054.[23-25]

Identification code	OSH19054
Empirical formula	$C_{10.67}H_{12}F_{6.67}O_{2.67}$
Formula weight	309.54
Temperature/K	150.00(14)
Crystal system	monoclinic
Space group	$P2_1/c$
a/Å	6.5607(2)
b/Å	9.2352(4)
c/Å	14.4625(6)
α/°	90
β/°	92.224(4)
γ/°	90

Volume/Å^3	875.61(6)
Z	3
ρ_{calc}g/cm^3	1.761
μ/mm^{-1}	1.746
F(000)	472.0
Crystal size/mm^3	0.294 × 0.141 × 0.092
Radiation	CuKα (λ = 1.54184)
2Θ range for data collection/°	11.37 to 147.8
Index ranges	-7 ≤ h ≤ 7, -11 ≤ k ≤ 11, -9 ≤ l ≤ 17
Reflections collected	3327
Independent reflections	1709 [R_{int} = 0.0296, R_{sigma} = 0.0303]
Data/restraints/parameters	1709/0/137
Goodness-of-fit on F^2	1.046
Final R indexes [I>=2σ (I)]	R_1 = 0.0368, wR_2 = 0.0970
Final R indexes [all data]	R_1 = 0.0407, wR_2 = 0.1011
Largest diff. peak/hole / e Å^{-3}	0.29/-0.23

Supplementary Table 5 | Fractional atomic coordinates (×10^4) and equivalent isotropic displacement parameters (Å^2×10^3) for OSH19054. U_{eq} is defined as 1/3 of the trace of the orthogonalised U_{IJ} tensor.

Atom	*x*	*y*	*z*	U(eq)
F001	-481.3(14)	2835.2(11)	2422.6(7)	27.9(3)
F002	3396.5(14)	1541.9(10)	2559.1(7)	26.7(2)
F003	1050.3(15)	1954.0(11)	4113.5(7)	30.1(3)
F004	-1247.6(15)	4429.3(13)	3972.4(7)	35.2(3)
F005	2574.4(16)	3675.2(14)	1308.6(7)	37.1(3)
O006	5296.2(17)	2082.4(12)	4202.0(7)	22.5(3)
O007	5889.8(19)	3470.7(13)	5462.7(9)	29.5(3)
C008	6148(2)	2352.1(18)	5057.1(11)	22.3(3)

C009	567(2)	4128.3(17)	2603.0(11)	23.3(4)
C00A	4109(2)	3230.8(17)	3782.7(11)	21.4(3)
C00B	4080(2)	2943.9(18)	2745.6(11)	22.3(3)
C00C	714(2)	4408.1(19)	3632.9(12)	24.5(4)
C00D	1989(2)	3309.2(18)	4173.1(11)	23.9(4)
C00E	7373(3)	1098.3(19)	5404.1(12)	26.4(4)
C00F	2707(2)	4028.4(19)	2240.8(11)	25.2(4)

Supplementary Table 6 | Anisotropic displacement parameters (Å^2×10^3) for OSH19054. The anisotropic displacement factor exponent takes the form: $-2\pi^2[h^2a^{*2}U_{11}+2hka^*b^*U_{12}+\ldots]$.

**Atom**	**U_{11}**	**U_{22}**	**U_{33}**	**U_{23}**	**U_{13}**	**U_{12}**
F001	21.2(5)	27.6(5)	34.2(5)	-1.4(4)	-7.1(4)	-4.4(4)
F002	30.0(5)	22.1(5)	27.7(5)	-4.2(4)	-4.4(4)	2.4(4)
F003	25.6(5)	27.8(5)	37.2(6)	8.7(4)	3.3(4)	-3.0(4)
F004	20.1(5)	44.1(7)	41.7(6)	-3.6(5)	4.4(4)	8.0(4)
F005	35.5(6)	51.6(7)	24.1(5)	9.6(5)	1.0(4)	7.8(5)
O006	22.0(6)	19.7(6)	25.1(6)	-2.0(4)	-7.4(4)	2.8(4)
O007	32.6(7)	24.5(6)	30.9(6)	-6.3(5)	-5.7(5)	0.5(5)
C008	18.4(7)	22.9(8)	25.4(8)	-0.7(6)	-3.0(6)	-3.2(6)
C009	18.4(8)	20.1(8)	31.0(8)	3.7(7)	-4.5(6)	-0.7(6)
C00A	17.6(7)	18.1(7)	28.1(8)	-0.4(6)	-4.5(6)	1.2(6)
C00B	18.2(7)	22.0(8)	26.8(8)	0.5(6)	0.3(6)	-1.3(6)
C00C	16.1(7)	23.9(8)	33.5(9)	-3.3(7)	-0.2(6)	1.8(6)
C00D	21.5(8)	25.4(8)	24.6(8)	-2.0(6)	-0.8(6)	1.7(7)
C00E	26.7(8)	26.4(8)	25.6(8)	-0.1(7)	-6.2(6)	1.4(7)
C00F	22.9(8)	26.0(8)	26.8(8)	6.2(7)	0.2(6)	-0.5(7)

Supplementary Table 7 | Bond lengths for OSH19054.

Atom	Atom	Length/Å	Atom	Atom	Length/Å
F001	C009	1.3974(18)	C008	C00E	1.486(2)
F002	C00B	1.3932(18)	C009	C00C	1.511(2)
F003	C00D	1.3959(19)	C009	C00F	1.521(2)
F004	C00C	1.3954(18)	C00A	C00B	1.522(2)
F005	C00F	1.386(2)	C00A	C00D	1.523(2)
O006	C008	1.3602(19)	C00B	C00F	1.515(2)
O006	C00A	1.4362(19)	C00C	C00D	1.513(2)
O007	C008	1.203(2)			

Supplementary Table 8 | Bond angles for OSH19054.

Atom	Atom	Atom	Angle/°	Atom	Atom	Atom	Angle/°
C008	O006	C00A	116.46(12)	F002	C00B	C00F	109.88(13)
O006	C008	C00E	111.23(14)	C00F	C00B	C00A	110.12(13)
O007	C008	O006	122.68(15)	F004	C00C	C009	108.93(13)
O007	C008	C00E	126.08(15)	F004	C00C	C00D	109.06(13)
F001	C009	C00C	110.02(13)	C009	C00C	C00D	114.04(13)
F001	C009	C00F	109.76(13)	F003	C00D	C00A	109.96(13)
C00C	C009	C00F	108.97(13)	F003	C00D	C00C	109.63(13)
O006	C00A	C00B	105.86(12)	C00C	C00D	C00A	109.56(13)
O006	C00A	C00D	111.51(13)	F005	C00F	C009	108.97(13)
C00B	C00A	C00D	113.39(13)	F005	C00F	C00B	109.07(14)
F002	C00B	C00A	110.13(13)	C00B	C00F	C009	114.44(13)

Supplementary Table 9 | Hydrogen atom coordinates (Å×10^4) and isotropic displacement parameters (Å^2×10^3) for OSH19054.

**Atom**	***x***	***y***	***z***	**U(eq)**
H009	-165.09	4929.39	2294.33	28
H00A	4810.49	4151.25	3907.19	26
H00B	5468.72	3046.35	2527.76	27
H00C	1326.36	5365.6	3734.74	29
H00D	2107.5	3606.85	4823.42	29
H00E	6568.07	520.21	5803.61	40
H00F	8564.47	1442.42	5742.65	40
H00G	7779.56	522.07	4890.39	40
H00H	3343.35	4985.67	2301.6	30

Supplementary Table 10 | Crystal data and structure refinement for SVH21029.[23-25]

Identification code	SVH21029
Empirical formula	$C_{18.67}H_{26.67}F_{6.67}N_{1.33}O_{5.33}$
Formula weight	481.75
Temperature/K	150.00(14)
Crystal system	monoclinic
Space group	$P2_1/c$
a/Å	5.04730(10)
b/Å	16.0606(3)
c/Å	20.3828(3)
α/°	90
β/°	94.174(2)
γ/°	90

Volume/Å^3	1647.90(5)
Z	3
ρ_{calc}g/cm^3	1.456
μ/mm^{-1}	1.250
F(000)	752.0
Crystal size/mm^3	0.744 × 0.121 × 0.097
Radiation	Cu Kα (λ = 1.54184)
2Θ range for data collection/°	7.014 to 145.902
Index ranges	-4 ≤ h ≤ 6, -19 ≤ k ≤ 19, -25 ≤ l ≤ 24
Reflections collected	10365
Independent reflections	3218 [R_{int} = 0.0274, R_{sigma} = 0.0217]
Data/restraints/parameters	3218/0/220
Goodness-of-fit on F^2	1.038
Final R indexes [I>=2σ (I)]	R_1 = 0.0365, wR_2 = 0.0926
Final R indexes [all data]	R_1 = 0.0396, wR_2 = 0.0952
Largest diff. peak/hole / e Å^{-3}	0.25/-0.20

Supplementary Table 11 | Fractional atomic coordinates (×10^4) and equivalent isotropic displacement parameters (Å^2×10^3) for SVH21029. U_{eq} is defined as 1/3 of the trace of the orthogonalised U_{IJ} tensor.

**Atom**	***x***	***y***	***z***	**U(eq)**
F001	5552.1(15)	8843.5(5)	4229.8(4)	34.0(2)
F002	4348.2(17)	7192.2(6)	4574.4(4)	40.0(2)
F003	3969.3(16)	7678.0(6)	3284.4(4)	39.8(2)
F004	2519.5(19)	9304.1(6)	3136.4(4)	44.2(2)
O005	3395.3(19)	8475.3(7)	5386.0(5)	36.2(3)
F006	406(2)	6554.6(6)	3724.6(6)	53.0(3)
O007	7227.1(18)	7257.5(6)	6799.7(5)	34.1(2)

O008	4067.3(19)	6288.9(6)	6492.6(6)	39.7(3)
O009	433(2)	9346.8(8)	5773.7(5)	46.3(3)
N00A	2837(2)	7551.0(7)	6761.9(5)	24.3(2)
C00B	2479(3)	8976.7(8)	5851.0(6)	26.1(3)
C00C	4308(3)	8971.2(8)	6465.3(6)	28.0(3)
C00D	4915(3)	7045.6(8)	6692.1(6)	26.7(3)
C00E	1776(3)	8399.0(9)	4779.7(6)	27.6(3)
C00F	3213(3)	8402.5(8)	6986.0(6)	25.6(3)
C00G	2831(3)	8976.1(8)	4270.4(6)	26.5(3)
C00H	1376(3)	8821.5(8)	3603.3(6)	26.7(3)
C00I	1758(3)	7486.5(9)	4587.1(7)	32.4(3)
C00J	1386(3)	7920.1(9)	3388.7(7)	29.2(3)
C00K	295(3)	7384.5(9)	3914.8(7)	32.3(3)
C00L	5973(3)	5621.1(9)	6361.9(9)	38.8(4)
C00M	7753(3)	5438.7(11)	6976.6(8)	43.5(4)
C00N	7511(4)	5860.7(12)	5780.2(8)	48.1(4)
C	4163(4)	4885.2(13)	6195.7(17)	87.4(10)

Supplementary Table 12 | Anisotropic displacement parameters (Å^2×10^3) for SVH21029. The anisotropic displacement factor exponent takes the form: $-2\pi^2[h^2a^{*2}U_{11}+2hka^*b^*U_{12}+\ldots]$.

**Atom**	**U_{11}**	**U_{22}**	**U_{33}**	**U_{23}**	**U_{13}**	**U_{12}**
F001	21.4(4)	43.5(5)	36.7(4)	-2.7(3)	-0.8(3)	-2.9(3)
F002	37.5(5)	36.7(5)	44.8(5)	1.8(4)	-4.1(4)	14.4(4)
F003	28.5(4)	56.3(6)	34.9(4)	-16.5(4)	5.2(3)	6.4(4)
F004	48.0(5)	52.1(6)	32.1(4)	12.1(4)	0.5(4)	-15.7(4)
O005	29.2(5)	56.5(7)	22.5(5)	-6.6(4)	-1.2(4)	15.3(5)

F006	54.9(6)	26.1(5)	76.3(7)	-11.2(4)	-6.7(5)	-3.2(4)
O007	17.6(5)	34.2(5)	50.1(6)	-4.1(4)	0.7(4)	-3.3(4)
O008	19.0(5)	26.9(5)	72.8(8)	-11.9(5)	0.2(5)	-0.1(4)
O009	48.3(7)	51.2(7)	38.3(6)	-10.2(5)	-5.1(5)	26.3(6)
N00A	19.1(5)	24.5(5)	29.4(6)	-1.3(4)	1.2(4)	-3.2(4)
C00B	30.2(7)	24.0(6)	24.7(6)	2.4(5)	5.7(5)	-0.2(5)
C00C	32.1(7)	26.6(7)	25.0(6)	0.0(5)	1.2(5)	-5.6(5)
C00D	21.7(6)	27.2(7)	30.9(7)	0.0(5)	1.4(5)	-2.3(5)
C00E	22.3(6)	38.6(7)	21.9(6)	-1.9(5)	1.1(5)	7.9(5)
C00F	28.7(7)	26.6(6)	21.5(6)	-1.1(5)	2.8(5)	-1.7(5)
C00G	23.6(6)	26.1(6)	29.3(7)	-4.2(5)	-1.4(5)	3.0(5)
C00H	25.1(6)	30.5(7)	24.3(6)	3.6(5)	0.6(5)	-1.9(5)
C00I	29.8(7)	32.8(7)	35.1(7)	7.1(6)	4.5(6)	3.4(6)
C00J	22.4(6)	37.2(8)	27.6(6)	-8.3(5)	-0.6(5)	2.1(5)
C00K	27.5(7)	24.6(7)	44.3(8)	-4.5(6)	-0.1(6)	-0.8(5)
C00L	21.8(7)	29.4(7)	64.7(10)	-9.7(7)	0.0(6)	4.0(6)
C00M	45.2(9)	39.8(8)	47.3(9)	11.9(7)	14.8(7)	12.1(7)
C00N	47.3(10)	60.0(11)	35.5(8)	-5.8(7)	-6.7(7)	21.2(8)
C	33.5(10)	37.1(10)	191(3)	-45.4(14)	0.6(13)	2.3(8)

Supplementary Table 13 | Bond lengths for SVH21029.

Atom	Atom	Length/Å	Atom	Atom	Length/Å
F001	C00G	1.3984(15)	N00A	C00F	1.4499(17)
F002	C00I	1.3921(16)	C00B	C00C	1.5005(18)
F003	C00J	1.3918(16)	C00C	C00F	1.5334(18)

F004	C00H	1.3852(15)	C00E	C00G	1.5168(19)
O005	C00B	1.3508(16)	C00E	C00I	1.517(2)
O005	C00E	1.4362(15)	C00G	C00H	1.5179(17)
F006	C00K	1.3903(16)	C00H	C00J	1.5125(19)
O007	C00D	1.2200(16)	C00I	C00K	1.517(2)
O008	C00D	1.3416(16)	C00J	C00K	1.510(2)
O008	C00L	1.4777(17)	C00L	C00M	1.516(2)
O009	C00B	1.1922(17)	C00L	C00N	1.514(3)
N00A	C00D	1.3418(17)	C00L	C	1.517(2)

Supplementary Table 14 | Bond angles for SVH21029.

Atom	Atom	Atom	Angle/°	Atom	Atom	Atom	Angle/°
C00B	O005	C00E	116.89(10)	F004	C00H	C00J	109.07(11)
C00D	O008	C00L	120.98(11)	C00J	C00H	C00G	113.83(11)
C00D	N00A	C00F	121.24(11)	F002	C00I	C00E	110.17(12)
O005	C00B	C00C	111.15(11)	F002	C00I	C00K	110.02(12)
O009	C00B	O005	122.82(12)	C00E	C00I	C00K	109.35(11)
O009	C00B	C00C	125.99(12)	F003	C00J	C00H	109.53(11)
C00B	C00C	C00F	110.50(11)	F003	C00J	C00K	110.09(11)
O007	C00D	O008	125.94(12)	C00K	C00J	C00H	109.34(11)
O007	C00D	N00A	123.84(12)	F006	C00K	C00I	109.18(12)
O008	C00D	N00A	110.23(11)	F006	C00K	C00J	108.91(12)
O005	C00E	C00G	109.32(11)	C00J	C00K	C00I	113.66(11)
O005	C00E	C00I	107.30(11)	O008	C00L	C00M	110.22(13)
C00G	C00E	C00I	114.14(11)	O008	C00L	C00N	109.53(13)

N00A	C00F	C00C	112.91(10)	O008	C00L	C	102.49(12)
F001	C00G	C00E	110.03(10)	C00M	C00L	C	110.20(18)
F001	C00G	C00H	109.58(10)	C00N	C00L	C00M	112.92(13)
C00E	C00G	C00H	110.02(11)	C00N	C00L	C	110.98(18)
F004	C00H	C00G	108.99(11)				

Supplementary Table 15 | Hydrogen atom coordinates (Å×10^4) and isotropic displacement parameters (Å^2×10^3) for SVH21029.

**Atom**	***x***	***y***	***z***	**U(eq)**
H00A	1253.05	7366.43	6671.96	29
H00B	4496.99	9532.87	6637.4	34
H00C	6049.77	8776.5	6363.04	34
H00E	-41.61	8567.42	4856.47	33
H00D	4430.7	8406.61	7376.79	31
H00F	1525.26	8622.25	7106.31	31
H00G	2542.01	9554.4	4401	32
H00H	-473.63	8998.63	3624.3	32
H00I	835.79	7166.26	4910.54	39
H00J	264.93	7857.98	2978.49	35
H00K	-1571.1	7534.43	3950.1	39
H00L	9074.81	5867.11	7037.81	65
H00M	8608.66	4910.22	6930.36	65
H00N	6704.14	5423.41	7350.78	65
H00O	6298.22	6045.43	5425.37	72
H00P	8483.78	5386.81	5640.47	72
H00Q	8727.87	6301.82	5905.82	72

HA	3142.73	4763.01	6563.43	131
HB	5215.32	4408.84	6099.13	131
HC	2984.34	5018.15	5819.23	131

Supplementary Discussion

Discussion on NMR experiments

***Me*M$_3$** shows broadening of cyclohexane resonances at ca. 5.5 – 4.5 ppm similar to **M$_3$** (Supplementary Fig. 19). Therefore, the broadening of cyclohexane resonances (5.5 – 4.5 ppm) in **M$_3$** can not be due to the formation of aggregates with intermolecular N-H hydrogen bonds (since no N-H hydrogen bonding is possible for ***Me*M$_3$**). It indicates that for both compounds, broadened proton resonances for cyclohexane moiety are due to ring flipping of cyclohexane, which is generally slower for all-*cis* fluorinated cyclohexanes,[26] and slow equilibrium between multiple folded states in solution as evident from corresponding FESs (Supplementary Fig. 26b and d). To further address this point we performed VT-NMR measurements in chloroform (Supplementary Fig. 20), in which **M$_3$** does not polymerize but folds, as evident from HOESY measurements (Supplementary Fig. 18). In this case similar broadening of cyclohexane resonances (ca. 5.5 – 4.5 ppm) to **M$_3$** in cyclohexane/chloroform (84:16 v/v) is observed. Cyclohexane resonances become sharp only at low temperatures (< 293 K) confirming that in cyclohexane-d_{12}/chloroform-*d* (84:16 v/v) broadening is due to the slow cyclohexane ring flipping and the formation of multiple folded states.

Close values of the diffusion coefficients for **M$_1$** and **Ref$_2$** (reference compound that can neither fold nor form aggregates in solution) (Supplementary Figs. 21 and 22) clearly demonstrate that in chloroform **M$_1$** exists in a molecularly dissolved state therefore broadening of cyclohexane and NH resonances can not be attributed to the formation of oligomeric species.

Discussion on seed preparation

During our initial attempts to prepare **M$_3^{Seed}$** we noticed that sonication of a 0.9 mM solution (cyclohexane/chloroform 84:16 v/v) at 273 K for 20 min did not lead to homogeneous seeds with low polydispersity, and instead long (ca. 1 µm) bundled fibers were obtained as indicated by AFM (Supplementary Fig. 27). Moreover, the obtained seeds did not provide reliable reproducibility and length control during seeded living supramolecular polymerization. Therefore, we decided to use ***Me*M$_3$** as an auxiliary during seed preparation. ***Me*M$_3$** – a semi monotopic monomer that does not polymerize, but could form complexes with **M$_3$**. Complexation reduces the concentration of available **M$_3$**, which can no longer be incorporated into polymer chains. This effect is called "sequestration" and it is one of the ways to control the length of cooperative supramolecular polymers.[27] Shorter polymers can be better homogenized when ultrasound is applied. AFM studies on seeds obtained by sonication of 0.9 mM solutions of **M$_3$** (84:16 cyclohexane/chloroform, 20 min, 273 K) with different amounts of ***Me*M$_3$** added revealed that the addition of ***Me*M$_3$** has a pronounced effect on the size of

seeds. We discovered that sonication of **M3** and ***Me*M3** in 3:1 mol/mol ratio (c_{tot} = 1.2 mM, cyclohexane/chloroform 84:16 v/v, 273 K, 20 min) leads to the formation of small fibers with relatively low PDI. Using equimolar amounts of **M3** and ***Me*M3** (0.9 mM each) resulted in more amorphous aggregates (Supplementary Fig. 27). Thermal depolymerization, studied by CD, also revealed decreasing thermal stability and the degree of aggregation of "seeds" when the equivalence of ***Me*M3** increases (Supplementary Fig. 28) as expected for the formation of **M3-*Me*M3** dimers.[27] We also attempted to carry out seeded LSP using "seeds" obtained without ***Me*M3**, however AFM revealed formation of non-uniform fibers with higher tendency to bundle (Supplementary Fig. 30).

Supplementary References

1. Horcas, I., Fernandez, R., Gomez-Rodriguez, J. M., Colchero, J., Gomez-Herrero, J. & Baro, A. M. WSXM: A software for scanning probe microscopy and a tool for nanotechnology. *Rev. Sci. Instrum.* **78**, 013705 (2007).
2. Rasband, W.S., ImageJ, U. S. National Institutes of Health, Bethesda, Maryland, USA, https://imagej.nih.gov/ij/, 1997-2018.
3. (a) Smulders, M. M. J., Nieuwenhuizen, M. M. L., de Greef, T. F. A., van der Shoot, P., Schenning, A. P. H. J. & Meijer, E. W. How to Distinguish Isodesmic from Cooperative Supramolecular Polymerization. *Chem. Eur. J.*, **16**, 362 – 367, (2010); (b) Jonkheijm, P., van der Schoot, P., Schenning, A. P. H. J. & Meijer, E. W. Probing the Solvent-Assisted Nucleation Pathway in Chemical Self-Assembly. *Science* **313**, 80-83 (2006).
4. Shyshov, O., Siewerth, K. A. & von Delius, M. Evidence for anion-binding of all-cis hexafluorocyclohexane in solution and solid state. *Chem. Commun.* **54**, 4353-4355 (2018).
5. Wang, J., Wolf, R. M., Caldwell, J. W., Kollman, P. A. & Case, D. A. Development and testing of a general AMBER force field. *J. Comput. Chem.* **25**, 1157-1174 (2004).
6. Jakalian, A., Bush, B. L., Jack, D. B. & Bayly, C. I. Fast, efficient generation of high-quality atomic charges. AM1-BCC model: I. Method. *J. Comput. Chem.* **21**, 132-146 (2000); (b) Jakalian, A., Jack, D. B. & Bayly, C. I. Fast, efficient generation of high-quality atomic charges. AM1-BCC model: II. Parametrization and validation, *J. Comput. Chem.* **23**, 1623-1641 (2002).
7. Wang, J., Wang, W., Kollman, P. A. & Case, D. A. Automatic atom type and bond type perception in molecular mechanical calculations, *J. Mol. Graph. Model.* **25**, 247260 (2006)
8. Hess, B., Kutzner, C., van der Spoel, D. & Lindahl, E. GROMACS 4: Algorithms for Highly Efficient, Load-Balanced, and Scalable Molecular Simulation. *J. Chem. Theory Comput.* **4**, 435–447 (2008).
9. Tribello, G. A., Bonomi, M., Branduardi, D., Camilloni, C. & Bussi, G. PLUMED 2: New feathers for an old bird. *Comput. Phys. Commun.* **185**, 604–613 (2014).
10. Jorgensen, W. L., Chandrasekhar, J., Madura, J. D., Impey, R. W. & Klein, M. L. Comparison of Simple Potential Functions for Simulating Liquid Water. *J. Chem. Phys.* **79**, 926–935 (1983).
11. Bussi, G., Donadio, D. & Parrinello, M. Canonical Sampling through Velocity Rescaling. *J. Chem. Phys.* **126**, 014101 (2007).
12. Berendsen, H. J. C., Postma, J. P. M., van Gunsteren, W. F., DiNola, A. & Haak, J. R. Molecular dynamics with coupling to an external bath. *J. Chem. Phys.* **81**, 3684–3690 (1984).
13. Essmann, U., Perera, L., Berkowitz, M. L., Darden, T., Lee, H. & Pedersen, L. G. A smooth particle mesh Ewald method. *J. Chem. Phys.* **103**, 8577–8593 (1995).
14. Hess, B., Bekker, H., Berendsen, H. & Fraaije, J. LINCS: A linear constraint solver for molecular simulations. *J. Comput. Chem.* **18**, 1463–1472 (1998).

15. Laio, A. & Parrinello, M. Escaping Free-Energy Minima. *Proc. Natl. Acad. Sci. USA* **99**, 12562–12566 (2002).
16. Barducci, A., Bussi, G. & Parrinello, M. Well-Tempered Metadynamics: A Smoothly Converging and Tunable Free-Energy Method. *Phys. Rev. Lett.* **100**, 020603 (2008).
17. Bartók, A. P., Kondor, R. & Csányi, G. On representing chemical environments. *Phys. Rev. B* **87**, 184115 (2013).
18. Gasparotto, P., Bochicchio, D., Ceriotti, M. & Pavan, G. M. Identifying and tracking defects in dynamic supramolecular polymers. *J. Phys. Chem. B* **124**, 589–599 (2020).
19. Himanen, L. et al. DScribe: Library of descriptors for machine learning in materials science. *Comp. Phys. Commun.* **247**, 106949 (2020).
20. Pedregosa, F. et al. Scikit-learn: machine learning in Python. *J. Mach. Learn Res.* **12**, 2825–2830 (2011).
21. Gasparotto, P. & Ceriotti, M. Recognizing molecular patterns by machine learning: an agnostic structural definition of the hydrogen bond. *J. Chem. Phys.* **141**, 174110 (2014).
22. Gasparotto, P., Meißner, R. H. & Ceriotti, M. Recognizing local and global structural motifs at the atomic scale. *J. Chem. Theory Comput.* **14**, 486–498 (2018).
23. Dolomanov, V., Bourhis, L. J., Gildea, R. J., Howard, J. A. K. & Puschmann, H. *J. Appl. Cryst.*, **42**, 339-341 (2009).
24. Scheldrick, G. M. *Acta. Cryst*. **A71**, 3-8 (2015).
25. Scheldrick, G. M. *Acta. Cryst*. **C71**, 3-8 (2015).
26. Keddie, N. S., Slawin, A. M. Z., Lebl, T., Philp, D. & O'Hagan, D. All-cis 1,2,3,4,5,6-hexafluorocyclohexane is a facially polarized cyclohexane. *Nat. Chem.* **7**, 483–488 (2015).
27. (a) Vantomme, G. et al. Tuning the Length of Cooperative Supramolecular Polymers under Thermodynamic Control. *J. Am. Chem. Soc.* **141**, 18278-18285 (2019); (b) Weyandt, E. et al. *Org. Mat.* **2**, 129-142 (2020).
